# Light-Driven Proton-Coupled Two-Electron Ligand Reduction Causes the Rearrangement of the Coordination Sphere in Cu(I) 4*H*‑Imidazolato Complexes

**DOI:** 10.1021/jacs.6c00920

**Published:** 2026-05-16

**Authors:** Luise Thomisch, Louis Blechschmidt, Niklas Klosterhalfen, Phil Köhler, Helmar Görls, Jens H. Tran, Julian Plitzko, Benjamin Dietzek-Ivanšić, Carolin Müller, Martin Schulz

**Affiliations:** † Institute of Physical Chemistry, 9378 Friedrich Schiller University Jena, 07743 Jena, Germany; ‡ Department Functional Interfaces, Leibniz Institute of Photonic Technologies, 07745 Jena, Germany; § Institute of Inorganic and Analytical Chemistry, Friedrich Schiller University Jena, 07743 Jena, Germany; ∥ Computer Chemistry Center, Friedrich-Alexander-Universität Erlangen-Nürnberg, 91052 Erlangen, Germany

## Abstract

The direct conversion of solar light into chemical energy, inspired by natural photosynthesis, presents a promising strategy for energy storage and chemical transformation. We investigate this approach using photoactive Cu­(I) complexes with the functional 4*H*-imidazolato ligand for storage of multiple photoredox equivalents. This study demonstrates that these complexes undergo light-driven reduction under a variety of reaction conditions involving different irradiation wavelengths, solvents, and electron/proton donors. We used single-crystal X-ray analysis together with NMR spectroscopy to characterize the isolated photoreduction product. This analysis supports a proton-coupled two-electron-transfer mechanism and reveals a significant structural transformation of the complex. By employing UV–vis spectroscopy alongside DFT calculations, we elucidated the mechanisms underlying the photoreduction process, including protonation of both exocyclic nitrogen atoms at the chelating binding site as a consequence of the two-electron reduction, resulting in the migration of the Cu­(I) bisphosphine fragment from the chelating binding site of the imidazolato ligand to form a trigonal-planar Cu­(I) 1*H*-imidazolato complex. This rearrangement drastically changes the shape of the molecule, exposing an additional binding site on the Cu­(I) center and making the N,N-binding site available for subsequent reactions.

## Graphical abstract

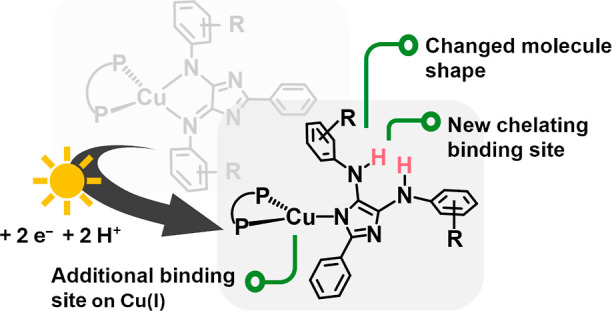

## Introduction

Harnessing solar energy is of significant interest in the development of sustainable energy solutions.^1,2^ Although the conversion of radiation into electric energy and subsequent storage in batteries are mature technologies, additional conversion steps are necessary to utilize the stored energy for chemical transformations.

Direct conversion of solar light into chemical energy, as occurs in natural photosynthesis, is a promising approach that not only facilitates energy directly for chemical transformations but also provides opportunities for energy storage.^3,4^ Mimicking the natural processes by utilizing multifunctional molecules designed to capture and convert abundant solar energy and drive reactions to store this energy in a usable form (solar fuels) is referred to as artificial photosynthesis.^5–7^ The generation of high energy products usually involves multielectron reactions.^8^ Therefore, in addition to efficiently absorbing light and selectively catalyzing substrate transformation, artificial systems should also facilitate the transfer and accumulation of multiple redox equivalents.^9,10^ In natural photosynthesis, light absorption triggers charge separation along the electron transport chain. Ultimately, this process results in the effective storage of energy by reducing NADP^+^ to NADPH. This molecule serves as a key energy carrier and reducing agent in the Calvin cycle, where it plays a vital role in carbon fixation and the synthesis of carbohydrates.^11^ In this example, charge accumulation is coupled with proton transfer, which is a key strategy that can also be applied to artificial systems.

Based on this principle, various molecular systems have been developed that combine light harvesting and charge accumulation, in which the ligand acts as an electron storage site. These systems include photosensitizer-acceptor assemblies,^12–19^ which require sacrificial electron donors, and donor-photosensitizer-acceptor triads^20^ or pentads,^21^ designed to operate independently of sacrificial donors. The photoactive component is typically a ruthenium polypyridyl moiety. However, relatively few examples exist where the photosensitizer incorporates a first-row transition metal center.^22^

We have developed functional Cu­(I) complexes, which combine light harvesting, charge accumulation, and storage of two photoredox equivalents within one molecule.^23,24^ Irradiation of Cu­(I) 4*H*-imidazolato complexes in the presence of an electron/proton donor leads to a two-electron reduction. In a light independent reaction, the reverse oxidation occurs when an electron acceptor is added. As such, the light and dark reactions can be separated in order to make the stored energy available on demand. In the key experiment, the Cu­(I) 4*H*-imidazolato complex **1** (see Figure 1) was quantitatively reduced over the course of 8 h under irradiation with white LED light and in the presence of an excess of the sacrificial donor *N*,*N*-dimethyl-*p*-toluidine (DMT) in acetonitrile. During the two-electron photoreduction, the dark violet, ligand-based polymethine chromophore of the initial state turns into a colorless aromatic chromophore. This can be observed through a decrease in the broad visible absorption band by UV–vis absorption spectroscopy. The resulting reduced species is stable under inert conditions but it readily reoxidizes when an electron acceptor, such as oxygen or methyl viologen, is added.^23^ The function-determining absorption process and the excited-state decay pathways were studied in detail.^25–27^

**Figure 1:**
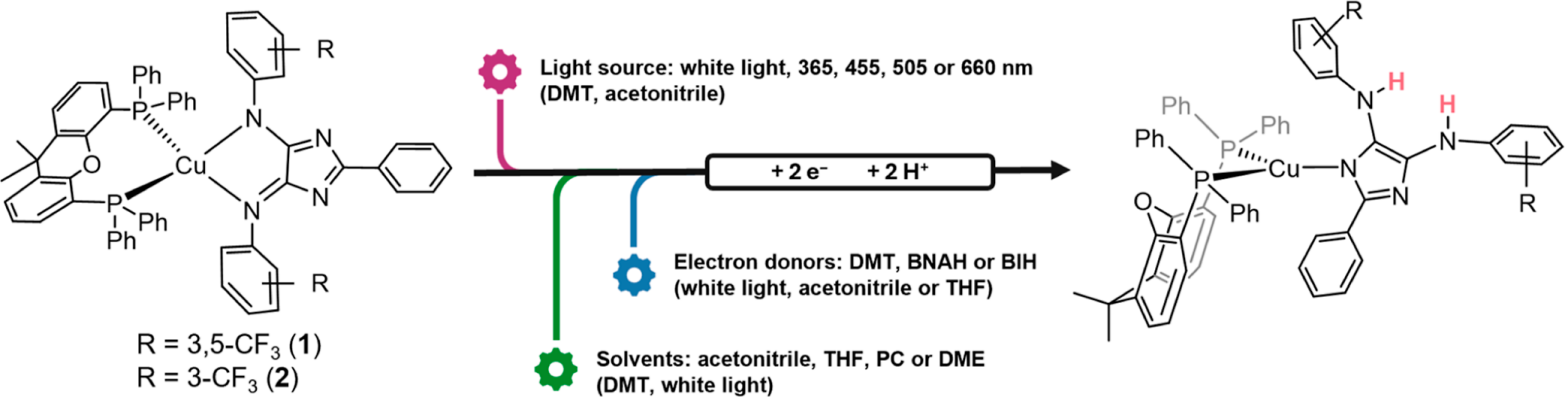
Representation of the investigated reaction conditions of the photoreduction of the 4*H*-imidazolato complexes **1** and **2** and the molecular structure of the photoreduction product.

However, little is known about the intermediate reduction steps or the identity of the doubly reduced species. Although spectroelectrochemical analysis suggested the presence of a one-electron-reduced intermediate, neither the characteristic absorption nor an EPR signal was detected during photoreduction with DMT. EPR spectroelectrochemical studies confirmed the presence of a ligand-based radical intermediate, but the broad, unstructured signal precluded detailed conclusions. NMR spectroscopy could trace the photoreduction, but excess DMT obscured key aromatic signals, preventing further identification of the two-electron reduction product.

Interestingly, the characteristic intermediate absorption signals were also absent in spectroelectrochemistry experiments in the presence of a proton source, suggesting a proton-coupled two-electron-transfer mechanism. On the basis of these observations, the following photoreduction pathways were considered: (i) photoreduction of the initial species to a transient intermediate with a reduction potential more positive than the oxidation potential of the electron donor. This results in a rapid, light-independent second reduction of the intermediate. (ii) Two sequential one-electron photoreduction processes for the initial species and the intermediate one-electron radical species. (iii) Disproportionation of two intermediate radical species to the initial oxidized species, as well as two-electron reduced species. (iv) Concerted (proton-coupled) two-electron photoreduction.

In this study, we summarize our approach to shedding light on the overall mechanism using spectroscopic and computational methods. To address the above-mentioned challenges, we investigated the effects of excitation wavelength, solvent, and sacrificial donor choice on the photoreduction. These experiments improved our understanding of the reaction and enabled structural elucidation of the two-electron reduced species and subsequent ab initio calculations on proton dependence. Our results from single-crystal X-ray diffraction, NMR, and TD-DFT revealed that the 2-fold proton-coupled photoreduction of the 4*H*-imidazolato ligand induces a significant rearrangement in the structure of the molecule. During this process, the Cu­(I) bisphosphine (PP) fragment moves from a chelating N,N-binding pocket to form a trigonal-planar complex with the reduced 1*H*-imidazolato ligand via a Cu­(I)–κN1 bond (Figure 1). This rearrangement reaction drastically changes the shape of the molecule, stabilizes the two-electron reduced state, and opens a binding site for potential reaction partners, e.g., in a reoxidation reaction.

For the present study, we worked with complexes **1** and **2** (Figure 1), which differ in the number of CF_3_ substituents. Our earlier work^23^ was conducted with complex **1**. Consequently, investigations into wavelengths and solvent dependencies were conducted with complex **1**. However, we obtained single crystals of the two-electron reduced species from complex **2**, owing to its lower solubility.^28^ Thus, we conducted X-ray diffraction and NMR experiments, as well as DFT calculations, with complex **2**. Complexes **1** and **2** exhibit comparable photoreactivity, and their UV–vis absorption and electrochemical properties are provided in the Supporting Information (Figures S1–S3).

## Results and Discussion

Typically, photoreduction is induced by LED white light irradiation and results in a decrease of the broad visible absorption band. The decrease is caused by the two-electron reduction of the ligand’s imidazole core and turns the solution from violet to colorless. The photoreduction yield is hence determined from the ratio between the initial and residual absorbance of the polymethine chromophore in the visible region of the spectrum. To test the reversibility of the photoreduction, the photoreduced solution is exposed to air and the recovery of the visible absorption band is followed by UV–vis absorption spectroscopy. The spectral characteristics during photoreduction and reoxidation in the visible to NIR region are used to assess the reaction mechanism, including the occurrence of side reactions or one-electron reduced intermediates.

### Wavelength Dependence of the Photoreactivity of **1** in Acetonitrile

In a previous study, we investigated the wavelength-dependent excited-state decay pathways of Cu­(I) 4*H*–imidazolato complexes upon excitation into the broad visible absorption band at 403, 500, 560, and 580 nm. We found that most of the excited-state population undergoes fast intersystem crossing into a triplet state within picoseconds, irrespective of the nature of the initial Franck–Condon state, i.e., metal-to-ligand charge transfer (MLCT) or intraligand charge transfer (ILCT) states. Only a minor excited-state population remains in the singlet manifold, where the decay processes and relaxation dynamics are strongly dependent on the excitation wavelength.^26^

Based on these findings, we examined the wavelength-dependent photoreduction reactivity of **1** in the presence of 100 equiv. DMT by in situ UV–vis spectroscopy under LED irradiation at 365, 455, 505, 660, and 970 nm (for spectral profiles, see Figure S39). The data are summarized in the Supporting Information (Table S1 and Figures S5–S9) and show that a reversible photoreduction reaction occurs at all wavelengths (except for 970 nm). The reaction is indicated by a decrease in the absorption band in the visible region, which occurs without a shift in the maximum at 506 nm or the formation of new spectral features. The observation indicates a similar reaction mechanism without a detectable formation of intermediates from UV to red light irradiation, provided these wavelengths are absorbed by the complex, regardless of whether the excitation occurs predominantly to the ILCT or the MLCT states.

### Solvent Dependence on the Photoreactivity of **1**

To explore how the solvent affects the photoreduction process, we studied the photoreactivity of **1** with a 100-fold excess of DMT and under irradiation with white LED light in acetonitrile (MeCN), 1,2-dimethoxyethane (DME), tetrahydrofuran (THF), and propylene carbonate (PC). Using UV–vis spectroscopy, we explored the solvent-dependent reaction kinetics, reversibility under oxygen exposure, and spectral characteristics of the photoreduction and subsequent reoxidation processes. We chose the solvents based on their polarity and viscosity^29^ (MeCN ε_R_ = 35.94; η = 0.345 × 10^–3^ Pa·s, DME ε_R_ = 7.20; η = 0.455 × 10^–3^ Pa·s, THF ε_R_ = 7.58; η = 0.575 × 10^–3^ Pa·s, PC ε_R_ = 64.9; η = 2.53 × 10^–3^ Pa·s). The photoreduction of **1** proceeds reversibly in all four solvents and clearly is solvent-dependent (Figures S10–S13 and Table S2). However, the data do not allow a clear correlation with the polarity or viscosity of the solvents. The lack of polarity dependence is an indicator that charged species might not play a dominant role and that charge compensation occurs effectively. Other (possibly unknown) solvent properties such as residual water content, proton conductivity, or H-bond donor/acceptor ability may influence the mechanism, e.g., on mass transport, electron transfer, cage escape, or protonation reactions. However, the availability of different solvents for the photoreduction reaction potentially broadens the scope of this reaction as well as the experimental basis. To confirm the reversibility of **1**’s photoreduction, reoxidation experiments were conducted by exposing the reduced solutions to atmospheric oxygen. The UV–vis spectral changes reflect reoxidation yields between 58% and 93%, with consistent band shapes (Figure S14 and Table S2). Reoxidation with oxygen likely produces equimolar amounts of water, contributing to slight spectral broadening.^27^

### Evaluation of Sacrificial Electron Donors

Our previous spectroelectrochemical investigations uncovered an overall proton-coupled two-electron reduction in the presence of ammonium hexafluorophosphate (NH_4_PF_6_).^23^ This observation is in accord with the photochemical reduction in the presence of DMT, which can act as a one-electron one-proton source.^30^ Photoreduction in the presence of DMT leads to the two-electron reduced Cu­(I) complex, and in situ UV–vis spectroscopy gave no indications for the one-electron reduced intermediate. To gain further insight into the photoreduction process and the potential photochemical accessibility of the protonated and unprotonated reduced species, we studied two different types of electron donors (cf. Figure 2). First, we chose the electron donors ferrocene (Fc) and cobaltocene (Cc) to study the single-electron reduction steps without proton compensation. The unprotonated, singly reduced and doubly reduced Cu­(I) complex species are electrochemically accessible and possess lower reduction potentials than the protonated species. Second, we applied the two-electron one-proton donors benzyl-1,4-dihydronicotinamide (BNAH) and 1,3-dimethyl-2-phenylbenzimidazole (BIH). These compounds possess higher reductive power than DMT and have the ability to donate two electrons or transfer a hydride.^31–33^

**Figure 2:**
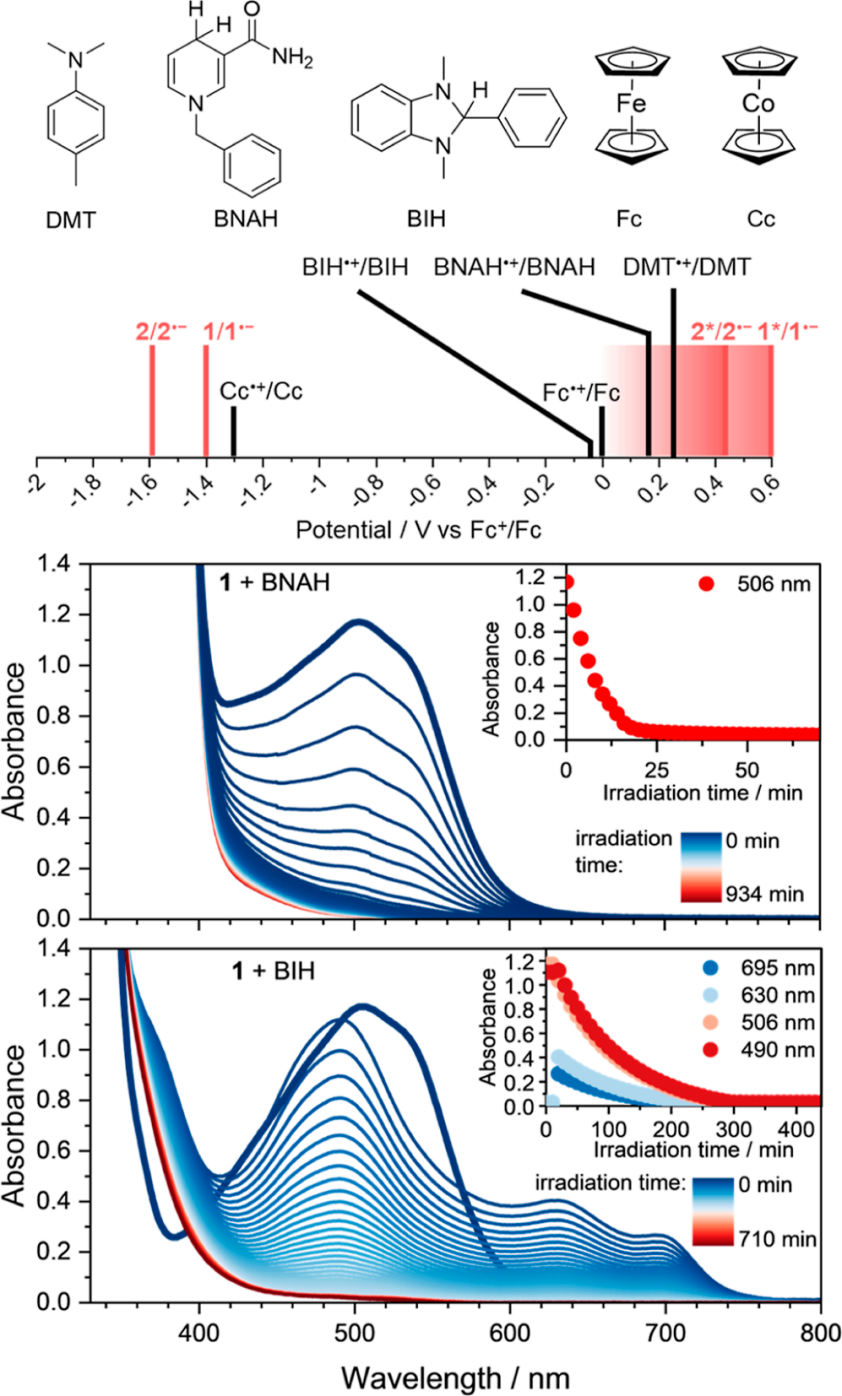
Top: comparison of the used electron donors and their oxidation potentials. For comparison, the graph gives the potentials for the first reduction step of **1** and **2**, as well as their respective excited-state reduction potential range. Excited-state potentials were estimated by the Rehm–Weller approach from the onset of the lowest-energy absorption and might be overestimated (**1** and **2** are not emissive; for further information, see Supporting Information). Middle: photoreaction of **1** (80 μM) with BNAH (100 equiv.) as electron donor in acetonitrile. The absorption spectrum is slightly changed due to the presence of BNAH in solution (broadening of the absorption band between 400 and 500 nm, and the maximum is shifted from 506 to 503 nm). Upon LED white light irradiation, the absorbance at the maximum decreased from 1.172 to 0.014 after a 934 min irradiation time, corresponding to a reduction of **1** to 1% of its initial concentration. Bottom: photoreaction of **1** (80 μM) with BIH (100 equiv.) as electron donor in acetonitrile. Upon LED white light irradiation, the absorbance at the maximum decreased from 1.170 to 0.015 after a 710 min irradiation time, corresponding to a reduction of **1** to 1% of its initial concentration.

Ferrocene is not able to reduce **1** in the ground state (*E*(**1**/**1** ^•–^) = −1.4 V; *E*(**1** ^•–^/**1** ^2–^) = −1.9 V vs Fc^+^/Fc), but with an excited-state energy of **1** of *E*_00_ ≈ 2.0 eV, estimated from the onset of the visible absorption band red edge, the photoreduction should be possible (note that **1** is not emissive). However, reacting **1** with Fc in a 100-fold molar excess under white light irradiation did neither produce the single nor the doubly reduced species. After 64 h of irradiation, the visible absorption band showed a small decrease of less than 3% and minimal spectral broadening (Figure S15). Also, in the presence of charge compensating electrolyte (TBAPF_6_, 0.06 M) or equimolar amounts of the proton donor NH_4_PF_6_ (80 μM, at 297 and 313 K), no reaction was observed (Figures S16 and S17). Thus, Fc failed as a suitable electron donor despite its thermodynamic potential to photoreduce **1**. The absence of a photoreaction even in the presence of ammonium as a proton source suggests that effective proton transfer is an important step to drive the reaction. Probably, the diffusive encounter with NH_4_PF_6_ is too slow to compete with electron back transfer to the donor (note that with higher concentration of NH_4_PF_6_, decomplexation occurs).

The photoreaction of **1** with a 100-fold excess of the much stronger reductant cobaltocene (*E*(Cc^•+^/Cc) = −1.3 V vs Fc^+^/Fc)^34^ initially showed a decrease in the visible absorption band and the appearance of a small NIR band (ca. 665 nm; Figure S18). However, the observed spectral changes could neither be assigned to the formation of the two-electron reduced species nor to the one-electron reduced species observed spectroelectrochemically and indicate the generation of other radical species of **1** and/or undesirable side products. This is also reflected by the increasing absorption above the initial value after an irradiation time of 19.5 h. Thus, Cc also failed to act as a suitable electron donor and, consequently, no further experiments involving Cc in the presence of proton donors were carried out at this point.

The two-electron one-proton donor compounds BNAH and BIH (*E*(BNAH^•+^/BNAH) = 0.17 V^35^ and *E*(BIH^•+^/BIH) = −0.07 V;^33^ vs Fc^+^/Fc) are stronger reductants than DMT (*E*(DMT^•+^/DMT) = 0.25 V^36^ vs Fc^+^/Fc).^37^ Irradiation of **1** with LED white light in the presence of a 100-fold excess of BNAH furnished a rapid photoreduction to a colorless product (Figure 2, middle). A reoxidation experiment with air confirmed the reversibility of the photoreduction (Figure S20). The spectral changes during photoreduction and reoxidation are comparable to those observed with DMT; however, the photoreduction proceeds much faster and reaches a photoreduction yield of 96% after 46 min and even 99% after ca. 16 h. Notably, the initial spectrum of the reaction solution shows an absorption feature around 450 nm, which cannot be explained by absorption of BNAH, as it does not absorb above 430 nm^38^ but may be due to the aggregation of both molecules in solution. Similar observations were also made for **2**. Irradiation of **2** with white LED light in the presence of a 100-fold excess of BNAH yielded a 98% photoreduction after 76 min (Figure S22).

Irradiation of complex **1** with white LED light in the presence of a 100-fold excess of BIH led to the rapid decrease of the visible absorption band, yet with a hitherto unobserved time-dependent spectral behavior (Figure 2, bottom). Immediately after the first irradiation interval, absorption bands are observed at 630 and 694 nm. At the same time, the absorption band of **1** decreased and the maximum shifted from 506 to 490 nm. Additionally, a shoulder appeared at ∼380 nm. During the ongoing irradiation, the bands at 630, 694, and 490 nm decreased. At long irradiation times (and low absorbance), the latter band became more structured. Eventually, a colorless solution was obtained, similar to that obtained with BNAH and DMT. The final spectrum and the reversibility test with atmospheric oxygen (Figure S19) suggest that photoreduction took place. The residual absorbance indicates a photoreduction yield of around 99% after 710 min. The absorption bands at 630 and 694 nm are indicative of the intermediate radical species, which so far has been observed only spectroelectrochemically.^23^

Irradiation of complex **2** with white LED light in the presence of a 100-fold excess of BIH leads to similar observations (Figure S21): within the first 5 min of irradiation, absorption bands appeared at 639 nm, 704, and 473 nm. With ongoing irradiation, these new features decreased, and a structured band appeared in the region between 400 and 600 nm, with sub-band maxima at 488 and 520 nm and shoulders at 460 and 560 nm. The absorption decreased further with ongoing irradiation, indicating the formation of a colorless reaction product. The reaction was reversible upon exposition to air. Like in the photoreduction of **1** with BIH, the first appearing bands during photoreduction are characteristic for the one-electron reduced radical intermediate (cf. Figure S4). This is supported by our TD-DFT simulations for **2** ^•–^ predicting dominant MLCT and ILCT absorption features of the singly reduced species between 400 and 600 nm (vide infra and Figure S45). Interpretation of the latter appearing structured band is more difficult. The wavelength range corresponds to the absorption of **2**. It has previously been observed that the appearance of absorption features of structurally similar Cu­(I) 4*H*-imidazolato complexes, consisting of broad MLCT and π–π* transitions (the latter being responsible for the substructure), is affected by the solvent polarizability and hydrogen bond donor/acceptor ability.^27^ The change in the chemical environment (possibly due to reaction intermediates and products) during the photoreduction could explain the different appearance of this absorption band. The overall temporal development at the different wavelengths suggests a complex mechanism, but its evaluation is beyond the scope of this paper. However, based on the breadth of spectroscopic, spectroelectrochemical, and computational data presented below, we conclude that the two-electron reduced product is the same regardless of whether BIH, DMT, or BNAH is used as the donor.

### Resolution of the Two-Electron Reduced Product via Single-Crystal X-ray Diffraction

Identification of the two-electron reduced product is important not only for the understanding of the mechanism but also for its application as a reductant in follow-up reactions. Here, the discovery of the chemical structure of the final reduction product ^exo^H_2_ **2** was key information for subsequent investigations. The high stability of the photoreduction product in an inert gas atmosphere makes it possible to isolate it from the reaction solution and investigate its structure by conventional methods such as 1D and 2D NMR spectroscopy or single-crystal X-ray diffraction. From the solvent-dependent photoreduction experiments, it is clear that low-polarity solvents are equally suited as, e.g., MeCN. Together with the lower solubility of **2** and with BIH as a powerful electron/proton donor, we eventually found suitable isolation conditions: complex **2** with 10 equiv. of BIH was subjected to photoreduction in THF. Subsequent layering of the reaction solution with cyclohexane led to the formation of pale yellow crystals suitable for single-crystal X-ray structure determination.

The two-electron reduction product of **2** crystallized in the triclinic space group *P*1̅ as neutral, doubly protonated complex ^exo^H_2_ **2** (Figure 3). The unit cell contains a hydrogen-bonded dimer along the inversion center with N–H···N hydrogen bonds between one protonated exocyclic N atom (N4) and the free endocyclic N atom (N2) of the adjacent molecule. Additionally, the second protonated exocyclic N atom (N3) forms an N–H···O hydrogen bond with one THF solvent molecule (see Figure S26). Donor–acceptor distances (N3···O2 2.883(2) Å, N4···N2′ 3.076(2) Å) and donor–H–acceptor angles below 170° are in accordance with weak H-bond interactions (N3–H3–O 161.0(16)°; N4–H4–N2′ 159.0(10)°). Noteworthy, the hydrogen atoms attached to N3 and N4 were explicitly found in the difference Fourier map and the refinement supports the absence of any cations in the crystal lattice, so that ^exo^H_2_ **2** was unambiguously identified as a neutral molecule (cf. Figure 3 and Supporting Information). This finding is further supported by the comparable solubility of **2** and ^exo^H_2_ **2** in THF or acetonitrile.

**Figure 3:**
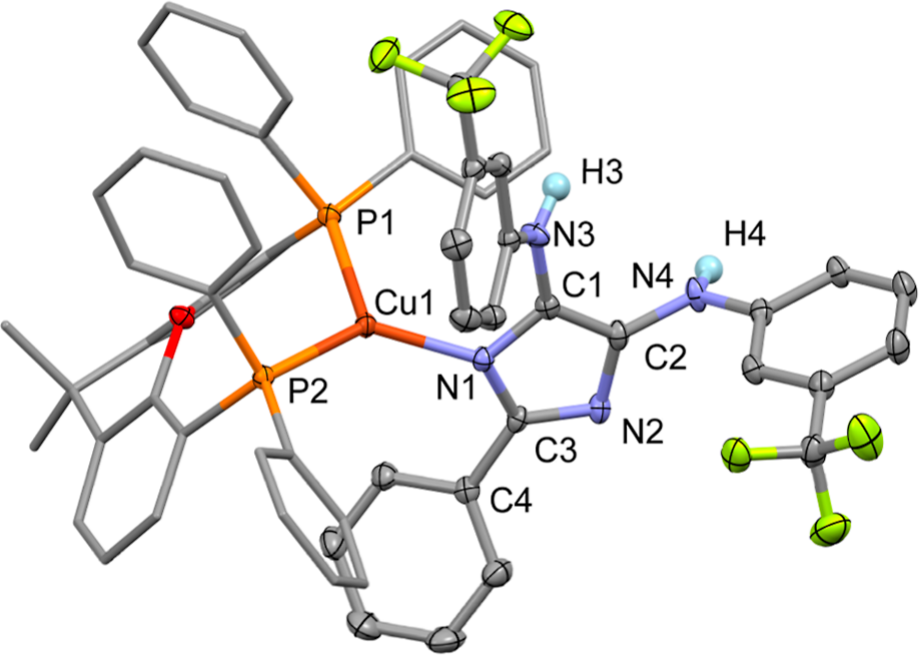
Molecular structure of ^exo^H_2_ **2** determined by single-crystal X-ray diffraction. Ellipsoids are either drawn at the 50% probability level or were omitted for the xantphos ligand. H atoms attached to C atoms and solvent molecules have been omitted for clarity.

One surprising feature of the structure of ^exo^H_2_ **2** is that the coordination sphere of the copper ion differs significantly from the original tetrahedral coordination of **2**. ^exo^H_2_ **2** shows a trigonal-planar coordination with a Cu­(I)–κN1 bond connecting the 1*H*-imidazolato ligand and coordination by the bidentate xantphos ligand. The internal angle sum of 360° (P1–Cu1–N1 114.22(5)°, N1–Cu1–P2 130.18(5)°, P1–Cu1–P2 115.60(2)°) confirms a trigonal-planar coordination. The P1–Cu–P2 plane is tilted by 74.40° from the imidazolate plane. The Cu–N and Cu–P bond lengths (Cu–N1 1.957(1) Å; Cu–P1 2.2845(5) Å; Cu–P2 2.2353(5) Å) and angles deviate only slightly from the tetrahedral environment of **2**, in which Cu is coordinated by the exocyclic N atoms as well as xantphos P atoms (P–Cu–P 116.64(2)°; Cu–P 2.2417(5) Å; 2.2868(5) Å; Cu–N 2.0984(15) Å; 2.1120(15) Å).^28^ The imidazole unit shows more drastic differences compared to the parent complex. In ^exo^H_2_ **2**, the bond length between C1 and C2 (1.376(2) Å) in the imidazole unit is about 0.12 Å shorter than in the 4*H*-imidazolato ligand of **2**.^28^ Additionally, the other imidazole ring bond lengths are between single and double bonds (C1–N1 1.368(2) Å, N1–C3 1.359(2) Å, C3–N2 1.344(2) Å, N2–C2 1.381(2) Å), while C3–C4 has single bond character (1.471(3) Å).^39^ The bond lengths between exocyclic N atoms and *ipso*-C atoms of the aryl rings are about 0.03 Å shorter in comparison to **2** (N3–C11 1.385(2) Å, N4–C17 1.379(2) Å), whereas lengths between imidazole C atoms and exocyclic N atoms are elongated by ca. 0.1 Å (C1–N3 1.400(2) Å, C2–N4 1.413(2) Å).^28^ The structural characteristics are typical for an aromatic 1*H*-imidazole and confirm the two-electron reduction of the 4*H*-imidazolato ligand. The additional charge is compensated by 2-fold protonation of the exocyclic N atoms.

Although the tetrahedral coordination is often preferred for the d^10^ configuration of Cu­(I) complexes, there are numerous three-coordinated Cu­(I) complexes known in the literature.^40^ Due to the absence of ligand field stabilization in the d^10^ configuration, the steric constraints govern the formation of the trigonal over the tetrahedral coordination. In most cases, the trigonal complexes bear halide or carbene ligands, but also other ligands are known to enable the trigonal coordination of Cu­(I).^40^ However, trigonal complexes of the [Cu­(PP)­(N^–^)]-type have rarely been described in the literature and the N^–^-ligand typically is an amide.^41,42^ The κN3–Cu­(I) 1*H*-imidazole binding motif has been extensively studied as this motif can be found in various Cu-based metalloproteins and their model complexes, such as hemocyanines.^43–46^ The coordination of N1-deprotonated imidazolates to Cu­(I) has been studied in coordination polymers where the imidazolate acts as a bridging ligand.^47–50^ Interestingly, mononuclear Cu­(I) imidazolato complexes in this coordination mode are very rare. To our knowledge, the only known Cu­(I) complex of the [Cu­(PP)­(N^–^)]-type where N^–^ is a benzimidazolate was reported by Li et al. in 2017.^51^ Here, the PP ligand is xantphos and Cu–N and Cu–P bond lengths, as well as bite angles, are comparable to those of ^exo^H_2_ **2**.

Attempts to crystallize the two-electron reduction product of **1** failed due to its higher solubility. However, crystallization of **1** was achieved from an acetone/water mixture (see the Supporting Information for details). Structural characteristics of **1** are consistent with those of similar Cu­(I) 4*H*-imidazolato complexes. A comparison of the characteristics of **1** and the published structure of **2** can be found in the Supporting Information (Table S5).

### Studying the Two-Electron Reduced Product in Solution via NMR Spectroscopy

To determine the structure of the reduced complex in solution, one- and two-dimensional NMR experiments were conducted. To obtain NMR data, either the isolated crystals were washed with cyclohexane, dried in vacuum, and dissolved in THF-*d*_8_ or the in situ photoreduction of **2** with BIH was conducted in THF-*d*_8_. Comparison of the ^1^H NMR spectra of the crystallized product with spectra taken from the in situ photoreaction confirmed that identical species were formed (Figure S33). The in situ photoreaction NMR data show additional signals from BIH and the BIH oxidation product, which partially overlap with the product signals (Figures S27 and S28).

Assignment of the NMR signals of the reduced complex was achieved by ^1^H,^1^H–COSY, HSQC, HMBC, and EXSY experiments. The NMR signals of the xantphos ligand are easily identified via methyl groups, showing two broad singlets at 1.95 and 1.49 ppm with an integral ratio of 1:1. The presence of cross signals in the EXSY as well as in the HMBC spectrum (corresponding ^13^C-signals at 32.1 and 25.2 ppm) reveals that the two signals arise from the same xantphos ligand and are interconverted. This observation is attributed to a hindered boat inversion of the xanthene unit, leading to separate signals for the axial and equatorial methyl group on the NMR time scale^52^ (Figure S35). While only one set of aromatic signals is present for the protons of the xanthene moiety (7.70 ppm, 7.11 ppm, 6.41 ppm; cf. X in Figure 4), there are two chemically nonequivalent phenyl spin systems (X-Ph and X-Ph′; Figure 4), indicating an asymmetric structure of the complex.^52^ The remaining three aromatic spin systems, observed in the ^1^H,^1^H–COSY spectrum (Figure S34), are assigned to the 2-phenyl ring (2-Ph) and the two *N*-aryl rings (N–Ar) of the imidazole ligand (Figure 4). Two broad signals at 6.32 and 6.83 ppm do not exhibit HSQC cross peaks and are assigned to the N–H protons. HMBC correlations of these protons with the NAr6/NAr′6 carbon signals confirm that protonation occurs at the exocyclic nitrogen atoms. Altogether, these NMR results are consistent with an asymmetric structure of the complex in solution comparable to that in the crystalline state, with different chemical environments of the *N*-aryl rings. This conclusion is further supported by the observation of two ^19^F NMR signals (−63.01 ppm and −63.03 ppm) and one ^31^P NMR signal (−17.63 ppm) (Figure S32). All ^1^H signals and most ^13^C signals could be assigned to the complex (note that ^13^C signals for NAr′5, NAr′3, NAr3, Im4 and Im5 and CF_3_ groups are not assigned due to overlapping signals or low signal intensity). We have no indication for the presence of species with different coordination geometries, tautomers, or free ligands. To gain further insight into the dynamic processes of the reduced complex in solution, we carried out ^1^H-EXSY and variable temperature ^1^H NMR experiments. The EXSY-spectrum (Figure 5) shows several intense exchange signals between X-Ph/X-Ph′, N–Ar/NAr′, and N–H protons. These observations can be rationalized by the dynamic exchange between the coordinating endocyclic N atoms, i.e., the C_2_ rotation of the imidazole ligand. This process is only possible by breaking the Cu–N bond and dissociating the imidazole ligand, enabling dynamic intermolecular ligand exchange (Figure 5). Breaking of the Cu–N bond would also ease the steric hindrance imposed by the imidazolato ligand and facilitate the X-Ph/X-Ph′ exchange. However, other dynamic processes, such as the boat inversion of the xanthene unit, may also cause the X-Ph/X-Ph′ exchange. In addition to the prominent EXSY signals, the spectrum also exhibits some detectable NOE signals, which mostly reflect the signals observed in the ^1^H,^1^H–COSY NMR spectrum. The NOE cross signals between N–H protons and the N–Ar2/N–Ar′2 protons confirm the proposed N–H signal assignment (Figure S36). It should be mentioned that NOE signals between 2-Ph and the xantphos ligand were not detected, probably owing to the rotational exchange of the imidazole ligand.

**Figure 4:**
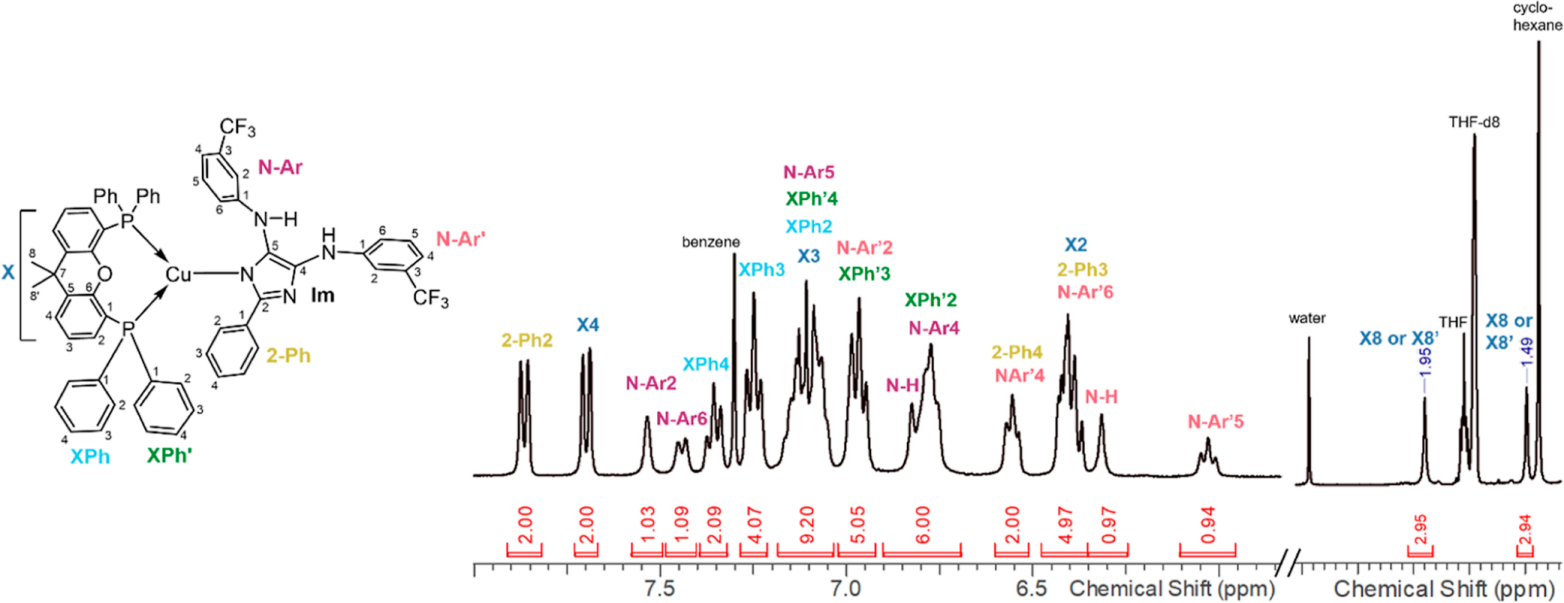
Left: structure of the reduced complex with atom labeling for NMR spectroscopic assignment and labeling of the spin systems, showing the inequivalence of the methyl groups, phenyl rings of the xantphos ligand, and N–Ar rings of the imidazole ligand. Right: ^1^H NMR spectrum of ^exo^H_2_ **2** (400 MHz, THF-*d*_8_, 297 K) with assignment to respective spin systems and atoms.

**Figure 5:**
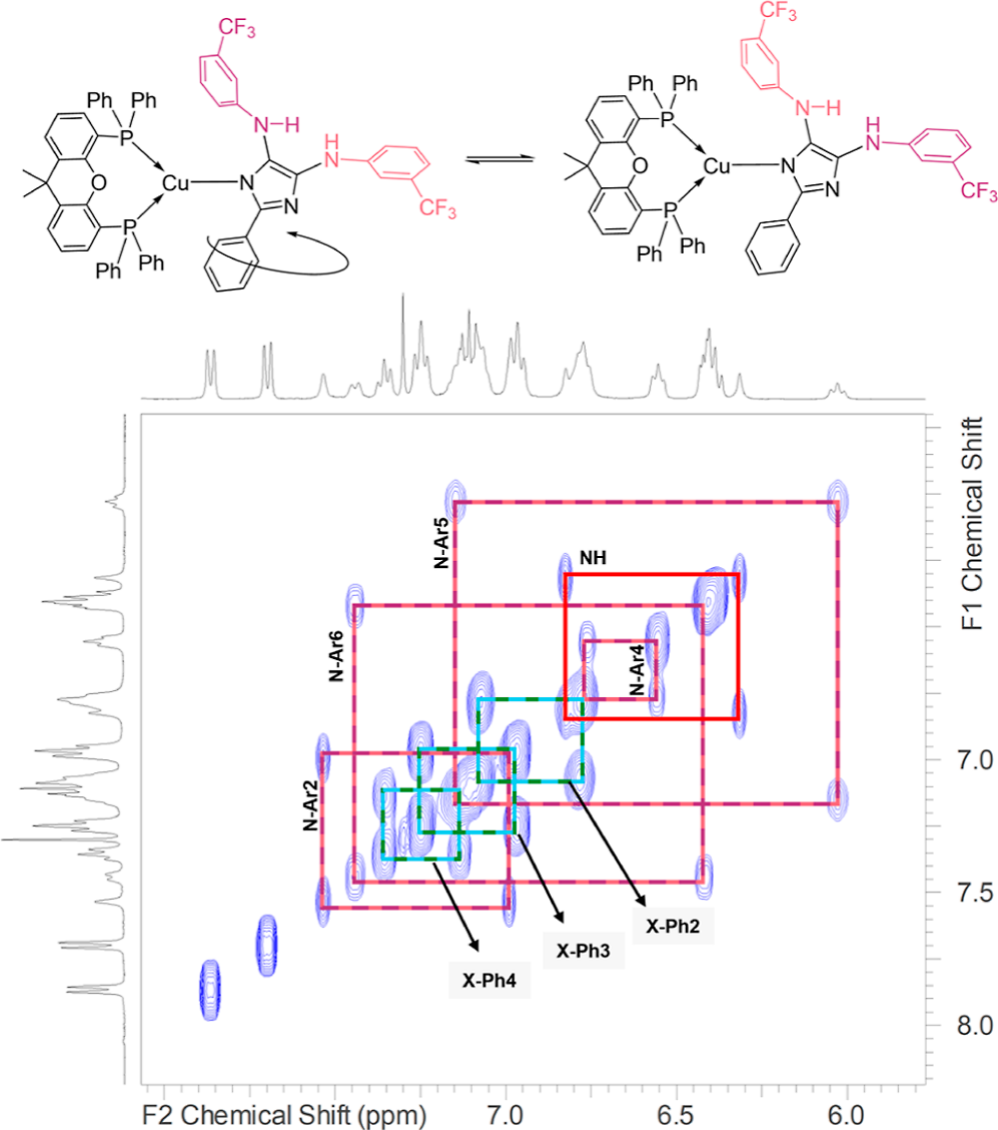
Top: dissociation of the 1*H*-imidazolato ligand and exchange of the N binding site cause the observed exchange of the N–Ar moieties in the EXSY spectrum. Bottom: aromatic region of the ^1^H,^1^H-EXSY-NMR spectrum (THF-*d*_8_, 400 MHz, 297 K) of the reduced complex. The exchange cross peaks are assigned to the respective H atoms of the N-aryl moieties (N–Ar) and xantphos-phenyl systems (X-Ph).

Variable temperature ^1^H NMR spectra (Figure 6) were recorded in the temperature range from 253 to 323 K. Even at elevated temperatures of 323 K, the signals for the two xantphos methyl groups (X8 and X8′) were observed and indicate that the hindered boat inversion persists in this temperature range. Except for the xanthene moiety, all signals are broadened at elevated temperatures, presumably due to accelerated exchange processes, though without reaching coalescence (except for the XPh2 signal; cf. Figure 4). Upon lowering the temperatures below r.t., most signals in the aromatic region only undergo minor shifts and broadening. However, strong temperature-dependent shifts are observed for the N–H signals, suggesting the presence of hydrogen bonds, probably with the solvent THF, residual water, or a neighboring complex (as found in the solid state). ^1^H-DOSY NMR results gave no indications for the presence of dimers in solution, by virtue of the molar weight estimated from the diffusion coefficient (external calibration method with normalized diffusion coefficients).^53^ From the determined diffusion coefficient of 5.88·× 10^–10^ m^2^ s^–1^, a molar weight of 1112 g·mol^–1^ was estimated and indicates a monomeric species (*M* = 1103.57 g·mol^–1^; for details, see Supporting Information). VT ^19^F NMR spectroscopy shows temperature-dependent broadening and shifts of the two singlet signals. However, no alteration in the integral values is observed (Figure S37). A temperature-dependent change of the ^19^F signal intensity would have been expected, if each signal belongs to a different reactant in a chemical equilibrium, e.g., between different coordination environments. The invariance of the ^19^F integral values supports the assignment of the ^19^F signals to asymmetric sites of the same imidazole ligand.

**Figure 6:**
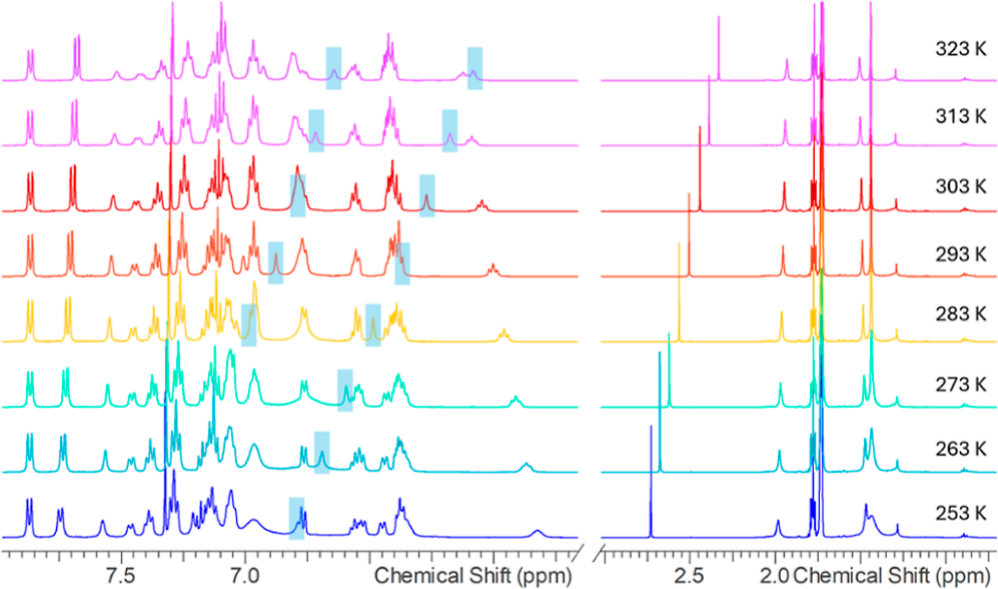
Temperature-dependent ^1^H NMR spectra (THF-*d*_8_, 500 MHz) of the reduced complex ^exo^H_2_ **2**. The positions of the N–H signals are highlighted in blue unless they fully overlap with other signals in the low-temperature spectra.

The NMR spectroscopy and X-ray diffraction results agree very well, and we hence conclude that the structure shown in Figure 3 also exists in solution. VT and DOSY NMR experiments gave no indication for the presence of hydrogen-bonded dimers in solution or exchange between different coordination geometries. Thus, in the investigated temperature range, the trigonal-planar coordinated structure is stable in solution, and hydrogen bonding does not seem to be the main driving force for the coordinative rearrangement.

X-ray diffraction and NMR results revealed that protonation is required for the formation of the final product and takes place at both of the exocyclic nitrogen atoms. This triggers the question on the origin of protons, likely connected with the oxidation mechanisms of the electron donors. DMT acts as a one-electron, one-proton source and leaves a radical intermediate, which dimerizes, preventing charge recombination.^30^ Therefore, two molecules of DMT are necessary to drive the photoreduction of **1** and **2**. The one-electron oxidation of BNAH leads to the very acidic intermediate BNAH^•+^. After deprotonation, BNA^•^ is a strong reductant and can donate a second electron (to form BNA^+^) but not the second proton. However, BNA^•^ may dimerize to form BNA_2_ and therefore BNAH can act as a one-electron, one-proton source, like DMT.^31,54^ Additionally, BNA_2_ can act as an electron donor as well.^55^ The one-electron oxidation of BIH leads to the acidic intermediate BIH^•+^. Following deprotonation, BI^•^ acts as a strong reductant (*E*(BI^+^/BI^•^) = −2.06 V vs Fc^+^/Fc),^33^ which does not dimerize but instead donates a second electron to form the BI^+^ cation.^56^ Therefore, it is considered a two-electron, one-proton donor (hydride donor). Formation of the photoreduction product ^exo^H_2_ **2** requires a second proton, which BIH cannot provide. Importantly, following the photoreduction with ^1^H NMR spectroscopy gave no indications for BI^+^ formation, missing the typical multiplets in the range between 8.05 and 7.69 ppm (see Figure S28). Instead, the observed BIH oxidation product shows ^1^H NMR signals at 7.29, 7.15, 7.06, 6.97, 6.57, and 6.29 ppm in the aromatic region, as well as one prominent signal at 171.53 ppm in the ^13^C NMR spectrum, indicating the presence of a carbonyl group (see Figures S28 and S30). These signals indicate the formation of *N*-methyl-*N*-[2-(methylamino)­phenyl]­benzamide (BIox) as the BIH oxidation product (see Figure S29). Bai et al. reported that BI^+^ reacts rapidly with OH^–^ to form BIox.^57^ As a test, BI^+^ was dissolved in THF-*d*_8_ and mixed with one drop of an aqueous NaOH solution. The NMR signals of BIox and those in the NMR spectrum after photoreduction match exactly (Figure S28) and prove that BIox is the final oxidation product of BIH, and that OH^–^ is present in the photoreduction solution. The OH^–^ anion likely originates from residual water and suggests a scenario in which two electrons and one proton are transferred from BIH to form BI^+^ and H**2** ^–^. The very basic H**2** ^–^ (vide infra) takes one proton from residual water, and the remaining OH^–^ reacts with BI^+^ to produce BIox.

With the applied precautions and experimental processes, three reasons are considered for the origin of residual water: (i) adsorbed water on the glass surfaces of NMR tubes, vials, and cuvettes, (ii) residual water content of the reactants, and (iii) residual water in the solvents. Even under optimal drying conditions for MeCN and THF,^58^ the water concentration would be sufficient to protonate the complex in the UV–vis irradiation experiments (see Supporting Information for details). From the experimental NMR data (see Figures 4 and S28), it is evident that the water concentration was high enough to protonate the reduced **2**.

The above observations and the lack of photoreactivity with metallocene donors, even in the presence of NH_4_PF_6_, suggest that protonation is intimately connected with the first reduction step and requires an electron donor and a proton donor reactant.

### DFT Analysis of Reactants, Intermediates, and Products

To rationalize the photoreaction cascade, we conducted theoretical investigations on both the one- and two-electron reduced complexes of **2**. To this end, we computed local minima of redox and protonation isomers of **2** and their respective UV–vis absorption properties by means of (TD-)­DFT simulations.

The simulated absorption spectrum of **2**, obtained as Boltzmann-weighted sum of the spectra of four different conformers (see Figures S41 and S42), reveals a visible absorption band characterized by two dominant features, S_1_ (538 nm, *f* = 0.172) and S_3_ (468 nm, *f* = 0.176). The respective transitions to these states are of MLCT (S_1_) and ILCT/MLCT (S_3_) characters (see charge density differences (CDDs) in Figure S42), which is in agreement with previous findings for Cu­(I) 4*H*-imidazolato complexes.^26,27^

For the doubly reduced doubly protonated complex, DFT calculations identified a complex with trigonal-planar coordination as the most stable structure (^exo^H_2_ **2**; see Figure S51). In this configuration, copper is coordinated by the endocyclic N atom, while protonation occurs at the two exocyclic N atoms. TD-DFT predicts for this species UV–vis absorption features that are mainly located below 350 nm, i.e., S_1_ at 331 nm (*f* = 0.179), which is in line with the experimental “bleaching” observation. According to the simulations, the dominant character of the UV–vis absorption features between 330 and 315 nm is ligand-to-ligand charge transfer transitions, where charge density is shifted from the 4*H*-imidazole to the xantphos ligand (see CDDs in Figure S52b). In contrast, protonation isomers featuring tetrahedral copper coordination are less stable, with relative energies increased by approximately 45–88 kJ·mol^–1^ with respect to ^exo^H_2_ **2**, depending on the specific protonation pattern: either mixed protonation at the imidazolate ring and exocyclic nitrogen atoms, or dual protonation at the imidazolate ring (Figure S51). TD-DFT calculations for these isomers predict absorption bands centered around 400 nm, which do not match the experimental spectrum (see Figure S52a), further supporting the assignment of ^exo^H_2_ **2** as the dominant species.

The doubly reduced singly protonated complex H**2** ^–^ is a potential reaction intermediate although not yet observed experimentally. For the calculated H**2** ^–^, coordination of copper to the endocyclic nitrogen in a trigonal-planar fashion is disfavored by 18–62 kJ mol^–1^, depending on the protonation side. Even the binding of copper in the chelating N–N pocket and monoprotonation of the imidazole is disfavored by about 20 kJ mol^–1^ (see Figure S49). The minimum structure of H**2** ^–^ is only obtained with copper in the chelating pocket and protonation of one exocyclic nitrogen atom. Likely, the strong basicity of the exocyclic nitrogen atoms leads to the rapid protonation and formation of ^exo^H_2_ **2**. Table S8 gives an overview of the calculated p*K*_a_ values. The p*K*_a_ of 22 for the protonation of H**2** ^–^ to give ^exo^H_2_ **2** further supports the sole existence of ^exo^H_2_ **2**. Like the doubly reduced and doubly protonated complexes with tetrahedral coordination geometry at the copper center, H**2** ^–^ shows in both, the trigonal-planar and tetrahedral coordination modes, prominent absorption features at about 408 nm (S_9_ and S_10_, respectively; see Figure S50).

For the singly reduced intermediate, both the unprotonated species (**2** ^•–^) and the singly protonated species (H**2** ^•^) were considered. The respective computational results indicate a preference for tetrahedral copper coordination in both cases, whereas the respective geometries of **2** ^•–^ and H**2** ^•^ are stabilized by 55 kJ mol^–1^ and 27–50 kJ mol^–1^ (depending on the protonation site) with respect to the trigonal-planar geometries (Figures S43 and S46).

Among the H**2** ^•^ tautomers, protonation at the endocyclic imidazolate nitrogen atoms is preferred by ca. 14 kJ mol^–1^ compared to the exocyclic sites (Figure S46). These energetic trends are reflected in the calculated p*K*_a_ values (Table S8): protonated trigonal-planar species exhibit acidic to slightly acidic character (e.g., p*K*_a_ ∼ 2.5), while tetrahedrally coordinated forms are more basic (p*K*_a_ up to ∼11).

However, a comparison of the experimental with the simulated absorption difference data for **2** ^•–^ and H**2** ^•^, both in the tetrahedral and trigonal-planar coordination modes, shows only partial agreement (cf. Figures S45 and S48). The calculated spectra suggest that experimental data are the sum of several individual components. Interestingly, the spectral profile of the radical(s) observed in spectroelectrochemistry experiments is the same as that obtained from photoreduction experiments, despite the different reaction environments. It seems likely that the presence of residual water in the spectroelectrochemistry experiments (vide supra) results in the formation of the same species observed in the photoreduction experiment. At this stage, it is not possible to draw further conclusions on the existence or absence of individual one-electron reduced species and further experiments in this direction are underway.

### Justification of a Light-Independent Second Electron Transfer

Based on the experimental data, we exclude a concerted two-electron transfer to the final product. Although the intermediate was only observed with BIH as donor, it seems plausible to exclude this pathway also for the other donors. Instead, we assume that when BNAH or DMT are used as donors, the intermediate is depleted rapidly, resulting in a small quasi-stationary concentration that prevents its experimental observation. Currently available data are insufficient to draw further conclusions since formation and consumption kinetics of the intermediate may be governed not only by redox potentials of the donor and p*K*_a_ values of the involved species but also by back-electron transfer or cage-escape kinetics. Additionally, hydrogen-bonding or π-interactions with the donor may play a role.^59,60^

A two-step one-electron reduction model is corroborated by the calculated reduction potentials (cf. Table S9; note that calculations were performed only for species with identical atomic composition). First, DFT predicts a one-electron reduction potential of *E*(**2**/**2** ^•–^) = 0.4 V. Furthermore, in agreement with experimental electrochemical data,^28^ the calculations indicate that the second reduction occurs at approximately the same potential as the first when the one-electron reduced intermediate is protonated (calc. *E*(H**2** ^•^/H**2** ^–^) ∼ 0.4 V).

These findings suggest that the second reduction step is light-independent, and this conclusion is supported by experimental evidence as depicted in Figure 7. In these experiments, the intermediate was produced by irradiating **2** in the presence of a lower concentration of BIH (3.5 or 1 equiv., respectively) to slow down the reaction and to enable tracking of small changes in the respective absorption features. The experiments were conducted using a 455 nm LED to enable the simultaneous irradiation and detection of the intermediate’s characteristic bands at 639 and 702 nm. After 5 min of irradiation, the typical features of the radical intermediate appear in the visible and near-infrared (NIR) regions of the spectrum. After irradiation is switched off, depletion of the radical intermediate does occur without an increase in the initial absorbance of **2** (Figure 7, upper panel). Therefore, back electron transfer from the sacrificial donor or disproportion reactions of the radical intermediate are unlikely to be the main reason for depletion. During the reaction, the spectral features of the intermediate continue to decrease until the spectrum eventually resembles that of **2** at a lower concentration. This observation suggests that a light-independent reaction of the intermediate results in the formation of the final colorless reduction product.

**Figure 7:**
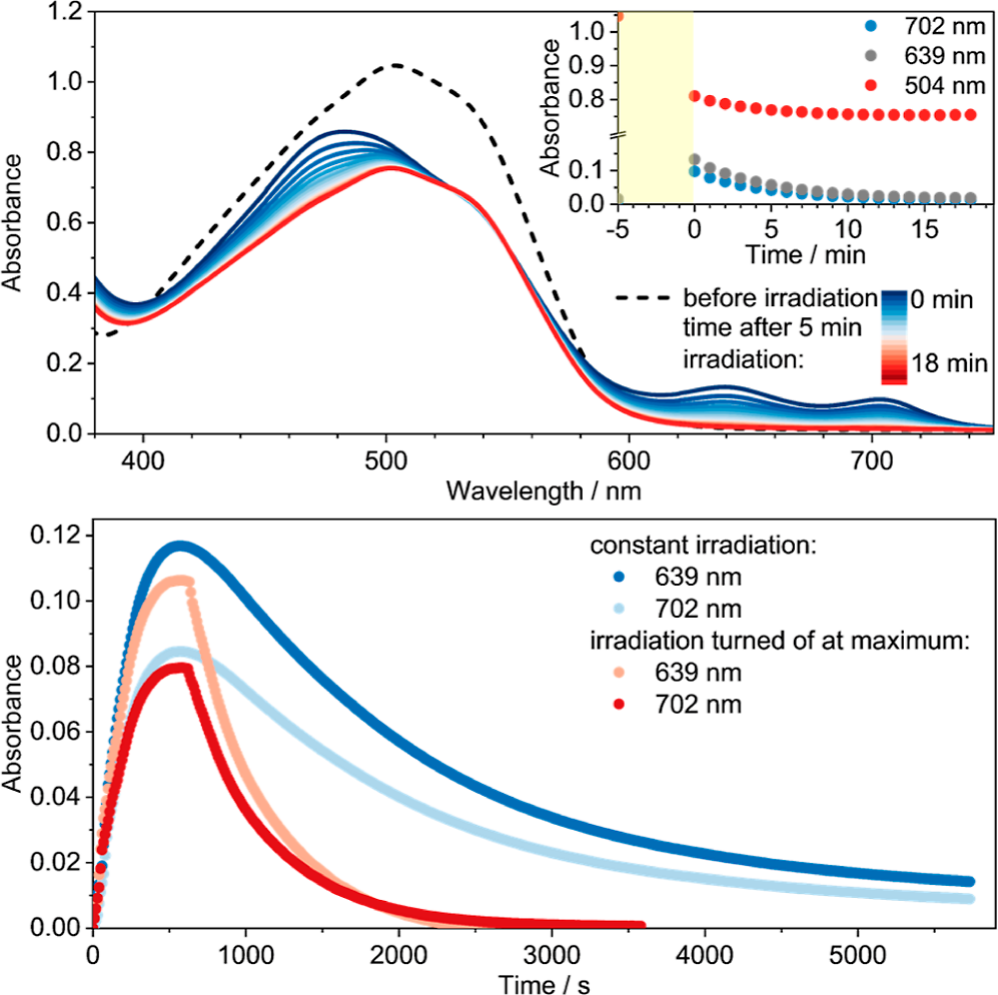
Upper panel: spectral changes after a 5 min irradiation time of **2** (60 μM) with BIH (3.5 equiv.) in acetonitrile. After a 5 min irradiation, the LED light source (455 nm) was switched off, and absorption spectra were recorded every 60 s. The spectral features of the radical intermediate decreased, and after 18 min, the absorbance at the maximum decreased from 1.047 to 0.755, corresponding to a reduction of **2** to 72.11% of its initial concentration. The decreased absorbance indicates that the radical intermediate is transformed into the colorless reduction product in a light-independent reaction. Lower panel: comparison of the absorption-time-profile of the radical intermediate bands in the photoreduction of **2** (60 μM) with BIH (1 equiv.) in acetonitrile at 639 and 702 nm. The absorption decreases much faster after the light source was switched off because no new intermediate is formed, and the intermediate is depleted in a light-independent reaction.

Figure 7 (lower panel) illustrates the absorption changes at 639 and 702 nm of the one-electron reduced intermediate over time and shows that the depletion reaction is faster in the dark. In one experiment, continuous irradiation was applied, whereas in the second experiment, it was stopped once the intermediate concentration reached its peak. In the first experiment, irradiation continuously formed a new one-electron reduced intermediate, resulting in a slower concentration decrease, whereas in the second experiment, the subsequent light-independent second reaction step rapidly depleted the intermediate species.

We also tracked the changes across the entire spectrum for the case of constant irradiation (Figure S23). The observations are similar to those of the white light experiments (vide supra), but photobleaching is incomplete. Once the intermediate features have been completely depleted, the spectrum resembles that of **2** and the bleaching process stops at a 31% conversion. Interestingly, a small increase in the absorption of **2** can be observed at longer irradiation times, possibly caused by redox reactions in the complex reaction mixture, but it was not investigated any further.

To study diffusion-dependent photoreactions, which may potentially occur simultaneously with the light-independent processes, we conducted transient absorption (TA) spectroscopy experiments of the one-electron reduced intermediate (see Supporting Information for details). The transient absorption profile of a solution of **2** in MeCN was recorded with and without the addition of BIH at a pump wavelength of 532 nm (see Figure 8). Without the addition of electron donor, the excited-state decay of **2** is consistent with that of previously investigated Cu­(I) 4*H*-imidazolato complexes.^26^ If BIH is present in the solution, the excitation of **2** and the subsequent photoreduction lead to the formation of the one-electron reduced intermediate **2** ^•–^. This results in both species being present in the laser spot, reflected by superimposed TA signatures of both excited species (see Figures 8 and S40). The ratio of both species was dependent on the amount of added BIH equivalents in the respective measurement. When 30 equiv. of BIH was added, the fraction of 2* is small, and thus the TA signature is predominantly composed of signals from the excited radical intermediate **2** ^•–^*. Using a global fitting algorithm and knowledge of the excited-state decay of **2**, we were able to extract the excited-state decay kinetics of the intermediate species. Its excited-state lifetime was determined to be 13 ps (see Supporting Information for details). This rules out diffusion-controlled reactions of the intermediate species in the excited state. Although these experiments enabled us to study the excited-state decay of the radical intermediate, they did not allow us to extract the involved electron transfer time between BIH and **2**. However, experiments to achieve this goal are underway.

**Figure 8:**
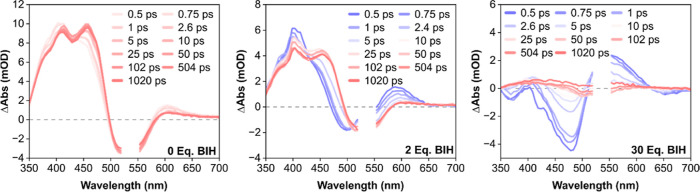
Transient absorption spectra of **2** in acetonitrile under inert conditions and at room temperature in the presence of different amounts of BIH: 0 equiv. (left), 2 equiv. (middle), and 30 equiv. (right). In all cases, excitation was performed at 532 nm and the wavelength region 525–555 nm was omitted due to strong pump scattering.

On the basis of all available experimental and theoretical results, a possible reaction pathway involving possible key intermediates is shown in Figure 9. After the first light activated electron transfer from BIH to **2,** the unknown radical intermediate(s) are formed. The calculations suggest that, following the initial reduction step, the endocyclic N atom with the highest p*K*_a_ value of 11.0 is protonated, resulting in the formation of ^endo^H**2** ^•^. The second reduction step occurs as a light-independent reaction where the highly reductive BI^•^ possibly acts as an electron donor. Immediately after the second reduction, a proton shift to the exocyclic N atom takes place, forming ^exo^H**2** ^–^, which is stabilized by 18 kJ/mol compared to ^endo^H**2** ^–^.

**Figure 9:**
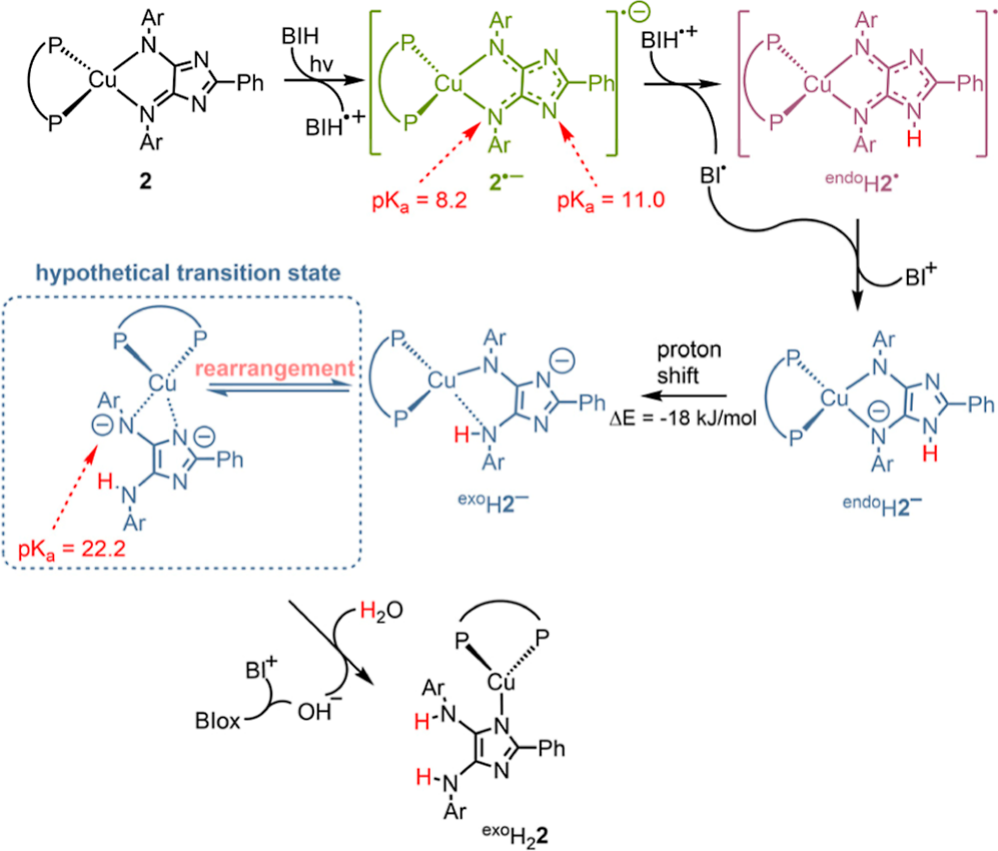
Proposed reaction path of the photoreduction of **2** with BIH as a sacrificial electron donor. Experimentally observed structures (black) are distinguished from calculated or hypothetical intermediates (colored). The p*K*_a_ values and energy differences are selected results from the calculations. The p*K*_a_ values are indicated on the respective conjugated bases.

According to our DFT results for ^exo^H**2** ^–^, the tetrahedral coordination is preferred over the trigonal geometry, but a rearrangement occurs to form ^exo^H_2_ **2**. Steric demand of the ligands hardly seems to be the main driving force, as tetrahedral coordination is the most stable geometry in the oxidized as well as the one-electron reduced state. Therefore, the new electronic situation arising from protonation of the exocyclic N atom is considered as the main driving force for the coordination rearrangement. It is reasonable that protonation of the first exocyclic N atom weakens the respective Cu–N bond and enables the Cu­(I) center to leave the chelating binding pocket (possibly via rotation around the exocyclic ArN–C bond). The highly basic second exocyclic N atom (p*K*_a_ value of 22) becomes protonated (e.g., by residual water to form OH^–^ and eventually BIox from BI^+^) leading to the cleavage of the second exocyclic Cu–N bond and formation of the trigonal geometry of ^exo^H_2_ **2,** which is stabilized by 40 kJ mol^–1^ compared to the tetrahedral geometry. The compensation of both excess charges leads to a neutral species, which seems to be very important for the stability of ^exo^H_2_ **2**.

Rearrangement of a copper coordination sphere is typically driven by the change of the metal redox state. For instance, oxidation of Cu­(I) to Cu­(II) causes a change from a tetrahedral to a square-planar or square-pyramidal coordination and is driven by ligand field stabilization.^59^ During the above-discussed rearrangement, the Cu­(I) redox state remains unchanged and due to its d^10^ electron configuration, ligand field stabilization is not in effect. Instead, the observed rearrangement is caused by the 2-fold reduction of the imidazolato ligand and the drastically increased basicity of the exocyclic nitrogen atoms. While protonation of one exocyclic N atom likely weakens the Cu–N bond, protonation of the second exocyclic N atom is necessary to stabilize the rearrangement product in a trigonal-planar geometry. Steric constraints do not seem to play a role, but the Cu­(I) cation compensates the excess charge of the 1*H*-imidazolate ring. As a consequence of the rearrangement, the shape of the molecule is drastically changed and the chelating N,N-pocket is available as a potential site for follow-up reactions, e.g., the binding of nucleophiles, such as CO_2_ for proton-coupled reduction.^60,61^ Likewise, the tricoordinate Cu­(I) center is potentially able to bind a fourth ligand and become involved in a follow-up reaction.^62,63^ Noteworthy, spontaneous hydrogen evolution is not observed from these complexes, despite the availability of two electrons and two protons, probably because both protons are of protic rather than hydridic character. These insights are an important step to shape future developments for the proton-coupled (two-electron) reduction of substrates, driven by the absorbed radiation. Understanding the stabilization mechanism of the two-electron reduced state now allows to tweak the molecular structure—less stabilized yet with higher reductive power—toward solar to chemical energy conversion. Besides these implications for artificial photosynthesis, redox reactions as trigger for molecular rearrangement processes of metal complexes^64,65^ are underexplored, and to the best of our knowledge, proton-coupled two-electron photoreduction has not been described before in this context. Rearrangement reactions or transformations induced by external triggers are intensively investigated, for instance, in biomimetic chemistry or host–guest chemistry.^66–68^

## Conclusion

This study sheds light on the mechanism and structural consequences of the photoinduced, proton-coupled, two-electron reduction of Cu­(I) 4*H*-imidazolato complexes. Through a combination of UV–vis spectroscopy, single-crystal X-ray diffraction, NMR spectroscopy, and DFT calculations, we demonstrate that photoreduction of the complexes occurs via sequential single-electron transfer steps involving a transient radical intermediate. Protonation is closely linked to electron transfer and accompanies a light-dependent initial and a light-independent subsequent reduction step. The overall process results in the formation of a neutral, doubly protonated product. This product is chemically stable under inert conditions and can be reversibly oxidized upon exposure to air or other electron acceptors. Structural analysis revealed that this two-electron photoreduction is accompanied by a significant rearrangement of the Cu­(I) coordination environment. Initially, the Cu­(I) complex is tetrahedral but rearranges into a trigonal-planar geometry coordinated by one endocyclic nitrogen atom of the reduced imidazolato ligand. Computational data confirm the stabilization resulting from the rearrangement; the trigonal-planar geometry of the reduced species is energetically favored by approximately 40 kJ·mol^–1^ over tetrahedral alternatives.

These findings represent an unprecedented example of a proton-coupled, two-electron photoreduction that drives a metal-coordination rearrangement. The observations provide new insight into light-driven, multielectron storage for future solar-to-chemical energy conversion systems, form the basis to study subsequent electron transfer to possible substrates, and might inspire the investigation of (photo)­redox-triggered rearrangement reactions.

## Supplementary Material



## Notes

Synthesis, photoreactions, single-crystal XRD data, NMR data, electrochemistry data, transient absorption data, computational details, and additional data (PDF)

## ⊥

Ulm University, Institute of Inorganic Chemistry I, 89081 Ulm, Germany

## #

Leibniz Institute of Surface Engineering (IOM), 04318 Leipzig, Germany.

## ¶

Innovent Technology Development, Department Surface Technology, 07745 Jena Germany.

## References

[1] Nocera D. G. (2009). Living
healthy on a dying planet. Chem. Soc. Rev..

[2] Dalle K. E., Warnan J., Leung J. J., Reuillard B., Karmel I. S., Reisner E. (2019). Electro- and Solar-Driven
Fuel Synthesis
with First Row Transition Metal Complexes. Chem.
Rev..

[3] Meyer T. J. (1989). Chemical
approaches to artificial photosynthesis. Acc.
Chem. Res..

[4] Lewis N. S. (2016). Research
opportunities to advance solar energy utilization. Science.

[5] Machín A., Cotto M., Ducongé J., Márquez F. (2023). Artificial
Photosynthesis: Current Advancements and Future Prospects. Biomimetics.

[6] Gust D., Moore T. A., Moore A. L. (2009). Solar fuels
via artificial photosynthesis. Acc. Chem. Res..

[7] Berardi S., Drouet S., Francàs L., Gimbert-Suriñach C., Guttentag M., Richmond C., Stoll T., Llobet A. (2014). Molecular
artificial photosynthesis. Chem. Soc. Rev..

[8] Appel A. M., Bercaw J. E., Bocarsly A. B., Dobbek H., DuBois D. L., Dupuis M., Ferry J. G., Fujita E., Hille R., Kenis P. J. A., Kerfeld C. A., Morris R. H., Peden C. H. F., Portis A. R., Ragsdale S. W., Rauchfuss T. B., Reek J. N. H., Seefeldt L. C., Thauer R. K., Waldrop G. L. (2013). Frontiers,
opportunities, and challenges in biochemical and chemical catalysis
of CO_2_ fixation. Chem. Rev..

[9] Hammarström L. (2015). Accumulative
charge separation for solar fuels production: coupling light-induced
single electron transfer to multielectron catalysis. Acc. Chem. Res..

[10] Bürgin T. H., Wenger O. S. (2021). Recent Advances and Perspectives in Photodriven Charge
Accumulation in Molecular Compounds: A Mini Review. Energy Fuels.

[11] El-Khouly M. E., El-Mohsnawy E., Fukuzumi S. (2017). Solar energy conversion: From natural
to artificial photosynthesis. J. Photochem.
Photobiol., C.

[12] Aslan J. M., Boston D. J., MacDonnell F. M. (2015). Photodriven
Multi-electron Storage
in Disubstituted Ru­(II) Dppz Analogues. Chemistry.

[13] Kobayashi K., Ohtsu H., Nozaki K., Kitagawa S., Tanaka K. (2016). Photochemical
Properties and Reactivity of a Ru Compound Containing an NAD/NADH-Functionalized
1,10-Phenanthroline Ligand. Inorg. Chem..

[14] Konduri R., de Tacconi N. R., Rajeshwar K., MacDonnell F. M. (2004). Multielectron
photoreduction of a bridged ruthenium dimer, [(phen)_2_Ru­(tatpp)­Ru­(phen)_2_] [PF_6_]_4_: aqueous reactivity and chemical
and spectroelectrochemical identification of the photoproducts. J. Am. Chem. Soc..

[15] Konduri R., Ye H., MacDonnell F. M., Serroni S., Campagna S., Rajeshwar K. (2002). Ruthenium Photocatalysts Capable of Reversibly Storing
up to Four Electrons in a Single Acceptor Ligand: A Step Closer to
Artificial Photosynthesis. Angew. Chem., Int.
Ed..

[16] Lefebvre J.-F., Schindler J., Traber P., Zhang Y., Kupfer S., Gräfe S., Baussanne I., Demeunynck M., Mouesca J.-M., Gambarelli S., Artero V., Dietzek B., Chavarot-Kerlidou M. (2018). An artificial
photosynthetic system for photoaccumulation
of two electrons on a fused dipyridophenazine (dppz)-pyridoquinolinone
ligand. Chem. Sci..

[17] Müller C., Schwab A., Randell N. M., Kupfer S., Dietzek-Ivanšić B., Chavarot-Kerlidou M. (2022). A Combined Spectroscopic and Theoretical Study on a
Ruthenium Complex Featuring a π-Extended dppz Ligand for Light-Driven
Accumulation of Multiple Reducing Equivalents. Chemistry.

[18] Ohtsu H., Tanaka K. (2012). An organic hydride transfer reaction of a ruthenium
NAD model complex leading to carbon dioxide reduction. Angew. Chem., Int. Ed..

[19] Tacconi N. R. d., Lezna R. O., Konduri R., Ongeri F., Rajeshwar K., MacDonnell F. M. (2005). Influence of pH on the photochemical and electrochemical
reduction of the dinuclear ruthenium complex, [(phen)_2_Ru­(tatpp)­Ru­(phen)_2_]­Cl_4_, in water: proton-coupled sequential and concerted
multi-electron reduction. Chemistry.

[20] Pannwitz A., Wenger O. S. (2017). Photoinduced Electron
Transfer Coupled to Donor Deprotonation
and Acceptor Protonation in a Molecular Triad Mimicking Photosystem
II. J. Am. Chem. Soc..

[21] Kuss-Petermann M., Orazietti M., Neuburger M., Hamm P., Wenger O. S. (2017). Intramolecular
Light-Driven Accumulation of Reduction Equivalents by Proton-Coupled
Electron Transfer. J. Am. Chem. Soc..

[22] Xie Z.-L., Gupta N., Niklas J., Poluektov O. G., Lynch V. M., Glusac K. D., Mulfort K. L. (2023). Photochemical
charge
accumulation in a heteroleptic copper­(I)-anthraquinone molecular dyad
via proton-coupled electron transfer. Chem.
Sci..

[23] Schulz M., Hagmeyer N., Wehmeyer F., Lowe G., Rosenkranz M., Seidler B., Popov A., Streb C., Vos J. G., Dietzek B. (2020). Photoinduced Charge
Accumulation and Prolonged Multielectron
Storage for the Separation of Light and Dark Reaction. J. Am. Chem. Soc..

[24] Schulz M., Dröge F., Herrmann-Westendorf F., Schindler J., Görls H., Presselt M. (2016). Neutral, heteroleptic copper­(I)-4H-imidazolate
complexes: synthesis and characterization of their structural, spectral
and redox properties. Dalton Trans..

[25] Schulz M., Reichardt C., Müller C., Schneider K. R. A., Holste J., Dietzek B. (2017). Excited State
Properties of Heteroleptic
Cu­(I) 4H-Imidazolate Complexes. Inorg. Chem..

[26] Seidler B., Sittig M., Zens C., Tran J. H., Müller C., Zhang Y., Schneider K. R. A., Görls H., Schubert A., Gräfe S., Schulz M., Dietzek B. (2021). Modulating
the Excited-State Decay Pathways of Cu­(I) 4H-Imidazolate Complexes
by Excitation Wavelength and Ligand Backbone. J. Phys. Chem. B.

[27] Müller C., Schulz M., Obst M., Zedler L., Gräfe S., Kupfer S., Dietzek B. (2020). Role of MLCT States
in the Franck-Condon
Region of Neutral, Heteroleptic Cu­(I)-4H-imidazolate Complexes: A
Spectroscopic and Theoretical Study. J. Phys.
Chem. A.

[28] Seidler B., Tran J. H., Thomisch L., Vashistha N., Görls H., Liebing P., Schulz M., Dietzek-Ivanšić B. (2023). Neutral, Heteroleptic
[Cu­(I)­(PPh_3_)_2_(4H-imidazolato)] Complexes: Ligand
Exchange Reactivity, Redox Properties, Excited-State Dynamics. Chemistry.

[29] Reichardt, C. Solvents and Solvents and Solvent Effects in Organic Chemistry: Third, Updated and Enlarged Ed.; Wiley-VCH Verlag GmbH & Co. KGaA, 2004.

[30] Rees N. V., Klymenko O. V., Compton R. G., Oyama M. (2002). The electro-oxidation
of N,N-dimethyl-p-toluidine in acetonitrile: a microdisk voltammetry
study. J. Electroanal. Chem..

[31] Pellegrin Y., Odobel F. (2017). Sacrificial electron
donor reagents for solar fuel
production. C. R. Chim..

[32] Lowe G. A. (2023). Enabling
artificial photosynthesis systems with molecular recycling: A review
of photo- and electrochemical methods for regenerating organic sacrificial
electron donors. Beilstein J. Org. Chem..

[33] Zhu X.-Q., Zhang M.-T., Yu A., Wang C.-H., Cheng J.-P. (2008). Hydride,
hydrogen atom, proton, and electron transfer driving forces of various
five-membered heterocyclic organic hydrides and their reaction intermediates
in acetonitrile. J. Am. Chem. Soc..

[34] Connelly N. G., Geiger W. E. (1996). Chemical Redox Agents
for Organometallic Chemistry. Chem. Rev..

[35] Fukuzumi S., Koumitsu S., Hironaka K., Tanaka T. (1987). Energetic comparison
between photoinduced electron-transfer reactions from NADH model compounds
to organic and inorganic oxidants and hydride-transfer reactions from
NADH model compounds to p-benzoquinone derivatives. J. Am. Chem. Soc..

[36] Ballardini R., Varani G., Indelli M. T., Scandola F., Balzani V. (1978). Free energy
correlation of rate constants for electron transfer quenching of excited
transition metal complexes. J. Am. Chem. Soc..

[37] The values from the respective references (vs SCE) were referenced to the Fc^+^/Fc redox couple by subtracting 0.4 V (cf. ref 34); in former publications we used the 0.35 V vs Fc^+^/Fc as DMT redox potential.

[38] Schreier M. R., Pfund B., Steffen D. M., Wenger O. S. (2023). Photocatalytic Regeneration
of a Nicotinamide Adenine Nucleotide Mimic with Water-Soluble Iridium­(III)
Complexes. Inorg. Chem..

[39] Allen F. H., Kennard O., Watson D. G., Brammer L., Orpen A. G., Taylor R. (1987). Tables of bond lengths
determined by X-ray and neutron
diffraction. Part 1. Bond lengths in organic compounds. J. Chem. Soc., Perkin Trans..

[40] Beaudelot J., Oger S., Peruško S., Phan T.-A., Teunens T., Moucheron C., Evano G. (2022). Photoactive Copper Complexes: Properties
and Applications. Chem. Rev..

[41] Lee K., Lai P.-N., Parveen R., Donahue C. M., Wymore M. M., Massman B. A., Vlaisavljevich B., Teets T. S., Daly S. R. (2020). Modifying
the luminescent properties of a Cu­(I) diphosphine complex using ligand-centered
reactions in single crystals. Chem. Commun..

[42] Lotito K. J., Peters J. C. (2010). Efficient luminescence
from easily prepared three-coordinate
copper­(I) arylamidophosphines. Chem. Commun..

[43] Pretzler M., Rompel A. (2018). What causes the different
functionality in type-III-copper
enzymes? A state of the art perspective. Inorg.
Chim. Acta.

[44] Solomon E. I., Sundaram U. M., Machonkin T. E. (1996). Multicopper
Oxidases and Oxygenases. Chem. Rev..

[45] Volbeda A., Hol W. G. (1989). Crystal structure
of hexameric haemocyanin from Panulirus
interruptus refined at 3.2 A resolution. J.
Mol. Biol..

[46] Kitagawa S., Munakata M. (1986). Nuclear Magnetic Resonance
Studies of Copper­(I) Complexes
of Imidazoles. I. Their Preparation, Characterization, Equilibria,
and Reaction with Carbon Monoxide. Bull. Chem.
Soc. Jpn..

[47] Zhang X., Li S., He Y.-J., Han T., Wang X.-L., Chen B., Zou K.-Y., Li Z.-X. (2015). Four Metal Complexes Based on Bulky
Imidazole Ligands: Solvothermal Syntheses, Crystal Structures, and
Fluorescence Properties. Z. Anorg. Allg. Chem..

[48] Huang X.-C., Zhang J.-P., Chen X.-M. (2004). A new route
to supramolecular isomers
via molecular templating: nanosized molecular polygons of copper­(I)
2-methylimidazolates. J. Am. Chem. Soc..

[49] Huang X.-C., Zhang J.-P., Lin Y.-Y., Chen X.-M. (2005). Triple-stranded
helices and zigzag chains of copper­(I) 2-ethylimidazolate: solvent
polarity-induced supramolecular isomerism. Chem.
Commun..

[50] Huang X.-C., Zhang J.-P., Chen X.-M. (2006). One-Dimensional
Supramolecular Isomerism
of Copper­(I) and Silver­(I) Imidazolates Based on the Ligand Orientations. Cryst. Growth Des..

[51] Huang M.-M., Guo Y.-M., Shi Y., Zhao L., Niu Y.-W., Shi Y., Li X.-L. (2017). Luminescent agostic Cu­(I) complexes containing both
trigonal planar and tetrahedral coordination modes. Inorg. Chim. Acta.

[52] Keller S., Pertegás A., Longo G., Martínez L., Cerdá J., Junquera-Hernández J. M., Prescimone A., Constable E. C., Housecroft C. E., Ortí E., Bolink H. J. (2016). Shine bright or live long: substituent effects in [Cu­(N̂N)­(P̂P)]^+^-based light-emitting electrochemical cells where N̂N
is a 6-substituted 2,2′-bipyridine. J.
Mater. Chem. C.

[53] Neufeld R., Stalke D. (2015). Accurate molecular
weight determination of small molecules
via DOSY-NMR by using external calibration curves with normalized
diffusion coefficients. Chem. Sci..

[54] Ishitani O., Yanagida S., Takamuku S., Pac C. (1987). Redox-Photosensitized
reactions. 13. Ru­(bpy)_3_
^2+^-Photosensitized reactions
of an NADH model, 1-benzyl-1,4-dihydronicotinamide, with aromatic
carbonyl compounds and comparison with thermal reactions. J. Org. Chem..

[55] Patz M., Kuwahara Y., Suenobu T., Fukuzumi S. (1997). Oxidation Mechanism
of NAD Dimer Model Compounds. Chem. Lett..

[56] Tamaki Y., Koike K., Morimoto T., Ishitani O. (2013). Substantial improvement
in the efficiency and durability of a photocatalyst for carbon dioxide
reduction using a benzoimidazole derivative as an electron donor. J. Catal..

[57] Bai Y., Li C., Sun W., Zhao G., Shi Z. (2008). Synthesis of N-methyl-N-[2-(methylamino)­phenyl]­carboxamide
derivatives. Huaxue Shiji.

[58] Williams D. B. G., Lawton M. (2010). Drying of organic solvents:
quantitative evaluation
of the efficiency of several desiccants. J.
Org. Chem..

[59] Hossain A., Bhattacharyya A., Reiser O. (2019). Copper’s rapid ascent in visible-light
photoredox catalysis. Science.

[60] Luo H., Li B., Ma J.-G., Cheng P. (2024). Molecular enhancement
of Cu-based
catalysts for CO_2_ electroreduction. Chem. Commun..

[61] Wang Z., Chen K.-K., Hao S., Wei Z., Wu K. (2025). Efficient
Photocatalytic CO_2_ Reduction Enabled by Biomimetic Proton-Coupled
Electron Transfer. CCS Chem..

[62] Mandal T., Katta N., Paps H., Reiser O. (2023). Merging Cu­(I) and Cu­(II)
Photocatalysis: Development of a Versatile Oxohalogenation Protocol
for the Sequential Cu­(II)/Cu­(I)-Catalyzed Oxoallylation of Vinylarenes. ACS Org. Inorg. Au.

[63] Song L., Cai L., van der Eycken E. V., Gong L. (2025). Recent advances in
copper-catalyzed multicomponent reactions with photoinduction. Coord. Chem. Rev..

[64] Goeb S., Sallé M. (2021). Electron-rich
Coordination Receptors Based on Tetrathiafulvalene
Derivatives: Controlling the Host-Guest Binding. Acc. Chem. Res..

[65] Zeid J. B., Dekhtiarenko M., Guechaichia R., Canevet D., Allain M., Freuze I., Sallé M., Goeb S. (2025). Redox-Induced Structural
Rearrangement in an M_8_L_2_ Self-Assembly. Chemistry.

[66] Badillo-Gómez J. I., Suarez-Antuña I., Mazurenko I., Biaso F., Pécaut J., Lojou E., Delangle P., Hostachy S. (2025). Biomimetic Pseudopeptides
to Decipher the Interplay between Cu and Methionine-Rich Domains in
Proteins. Chemistry.

[67] Benchimol E., Nguyen B.-N. T., Ronson T. K., Nitschke J. R. (2022). Transformation
networks
of metal-organic cages controlled by chemical stimuli. Chem. Soc. Rev..

[68] Spallacci C., Chino M., Rosato A., Maglio O., Huang P., D’Amario L., Lombardi A., Andreini C., Cheah M. H. (2025). A bioinformatics
approach to design minimal biomimetic metal-binding peptides. Commun. Chem..

