# Supplementary material for: Light-Driven Proton-Coupled Two-Electron Ligand Reduction Causes the Rearrangement of the Coordination Sphere in Cu(I) 4H‑Imidazolato Complexes
Source: J Am Chem Soc. 2026 May 16;148(21):21633–45. doi: 10.1021/jacs.6c00920 (PMC13244457; doi:10.1021/jacs.6c00920)
Supplement: Supplementary file 1 [file ja6c00920_si_001.pdf]

Supporting Information

Lightdriven proton-coupled two-electron ligand reduction causes the rearrangement of the coordination sphere in Cu(I) 4*H*-imidazolato complexes

Luise Thomisch, Louis Blechschmidt, Niklas Klosterhalfen, Phil Köhler, Helmar Görls, Jens H. Tran, Julian Plitzko, Benjamin Dietzek-Ivanšić, Carolin Müller*, Martin Schulz*

Content

1 General experimental conditions 4

1.1 Chemicals and solvents 4

1.2 Notes on water content in used chemicals and solvents 4

1.3 Synthesis 4

1.4 Electrochemistry 5

1.5 UV-Vis spectroelectrochemistry 5

1.6 UV-Vis spectroscopy 6

1.7 UV-Vis irradiation experiments 6

1.8 NMR spectroscopy 6

1.9 Fs-TA spectroscopy 7

2 Absorption properties of **1** and **2** 7

3 Electrochemical properties of **1** and **2** 8

4 Estimation of the excited state redox potential 9

5 Spectroelectrochemical properties of **1** and **2** 9

6 Photoreduction experiments 11

6.1 Photoreduction and reoxidation of **1** using DMT (100 eq.) as electron donor and irradiation with different wavelength 11

6.2 Photoreduction of **1** in different solvents using DMT (100 eq.) as electron donor under white light irradiation 14

6.3 Photoreduction of **1** using different electron donors in acetonitrile 16

6.4 Photoreduction of **2** using different electron donors in acetonitrile 20

7 Single-crystal X-Ray structural analysis of **1** and $^{exo}H_2$**2** 24

8 NMR spectroscopic investigations 27

8.1 NMR spectroscopic following of the photoreduction of **2** with BIH 27

8.2 NMR spectroscopic characterization of $^{exo}H_2$**2** 29

9 Spectral characteristics of LEDs used in the irradiation experiments 35

10 Fs-transient absorption spectroscopy of **2** and radical intermediate 35
11 DFT calculations 38
11.1 Computational details 38
11.2 Neutral complex 38
11.3 Singly-reduced complex 39
11.4 Singly-reduced, singly-protonated complex 41
11.5 Doubly-reduced, singly-protonated complex 43
11.6 Doubly-reduced, doubly-protonated complex 44
11.7 Calculated pK_a values and redox potentials 45
References 46

1 General experimental conditions

1.1 Chemicals and solvents

For experiments under nitrogen atmosphere acetonitrile, 1,2-dimethoxyethane (DME) and tetrahydrofuran (THF) were purified by distillation over calcium hydride under nitrogen atmosphere. THF-d8 was purified by distillation over sodium under nitrogen atmosphere. Propylene carbonate (PC) was purified by distillation under nitrogen atmosphere and stored over mol sieve 3 Å. Prior to each experiment, the respective solvent was degassed by at least three freeze-pump-thaw cycles. For experiments under atmospheric conditions, solvents were used as received and were of UV/IR-grade.

All chemicals were obtained from commercial sources and used as received, if not otherwise stated. DMT was distilled and degassed by freeze-pump-thaw technique, prior to use. Ferrocene and cobaltocene were purified by sublimation prior to use. Ammoniumhexafluorophosphate (NH_4PF_6) was dried under vacuo and stored in the glovebox under nitrogen atmosphere. Chemicals used in electrochemical experiments were of electrochemical grade.

1.2 Notes on water content in used chemicals and solvents

While the reactants were used as received (if not mentioned otherwise), the used solvents were dried using the above-mentioned procedures and stored over molsieve (3 Å) in the glovebox. Typically, this drying process, leaves a small amount of water in the solution.[1] Under optimal drying conditions the residual water concentration is 0.203 mM in THF and 0.078 mM in acetonitrile.[1] Even under these optimal conditions, the water concentration would be sufficient to protonate the complex in the UV-Vis irradiation experiments (0.08 mM, see chapter 6). Considering that the drying process was not controlled and water is also adsorbed on the glass surfaces of the used glassware, the water concentration might be even higher. THF-*d8* was distilled over Na/benzophenone and stored in the glovebox without molsieve. Under these conditions the water concentration is higher than under optimal conditions (2.123 mM).[1]

1.3 Synthesis

1.3.1 Cu(I)-4*H*-imidazolato complexes 1 and 2 were reported before.[2,3]

1 was crystallised from a water /acetone mixture.

1.3.2 1,3-dimethyl-2-phenyl-1*H*-benzo[d]imidazole-3-ium hexafluorophosphate (BI^+)

For the experiments BI^+ was synthesized with modifications of a reported procedure.[4]

2-phenyl-1*H*-benzo[d]imidazole: Ortho-nitroaniline (38 mmol, 1 eq.) and benzaldehyde (38 mmol, 1 eq.) were dissolved in EtOH (150 ml) and stirred at 70 °C for 1 hour. A 1 M sodium dithionite solution (120 ml) was then added to the reaction mixture and the reaction solution was stirred overnight at 70 °C. A 5 M ammonia solution (40 ml) was added to the hot solution. The resulting precipitate was filtered, washed with cold water (3 x 30 ml) and dried in a desiccator over concentrated sulphuric acid for one week. The product was isolated as white crystals (56%). The NMR signals correspond to those reported in the literature.[4]

^{1}H–NMR (300 MHz, DMSO-d6, 297 K) δ/ppm = 7.18-7.23 (m, 2H) 7.47 -7.61 (m, 5H) 8.16-8.20 (m, 2H) 12.54-13.21 (b, 1H).

1,3-dimethyl-2-phenyl-1*H*-benzo[d]imidazole-3-ium hexafluorophosphate: Under inert atmosphere, sodium hydride (16 mmol, 2 eq.) was dissolved in dried THF (200 ml). While stirring, 2-phenyl-1*H*-benzo[d]imidazole (8 mmol, 1 eq.) dissolved in THF (250 ml) was added dropwise to the reaction solution under cooling with ice and afterwards stirred for 30 min. Dimethylsulphate (4.2 ml, 46 mmol, 6 eq.) was added and the reaction mixture was refluxed at 70 °C overnight. The solution was then brought to room temperature and 5 M ammonia solution (70 ml) were added and stirred for 30 min. After removal of the solvent, the residue was dissolved in EtOH (60 ml) and a saturated solution of NH_4PF_6 (60 ml) was added and then stored overnight at -20 °C. The colorless precipitate was filtered and washed with water (3 x 20 ml). The product was isolated as colorless crystals (95%).

^{1}H–NMR (300 MHz, DMSO-d6, 297 K) δ/ppm = 3.89 (s, 6H) 7.74-7.92 (m, 7H) 8.10-8.17 (m, 2H).

1.4 Electrochemistry

A three-electrode electrochemical cell containing a 3 mm diameter glassy carbon disk working electrode, a platinum wire counter electrode, and an Ag/AgCl reference electrode (ET-072, eDAQ) was used for all electrochemical measurements. Ferrocene was added to each sample solution before the last cyclic voltammogram was recorded, to reference the potentials against the ferrocene/ferrocenium redox couple (Fc^+/Fc). Potentials were applied using a Zennium Pro Potentiostat (Zahner-Elektrik, Germany) and the corresponding Thales software. Prior to each experiment, the glassy carbon electrode was polished using an aqueous suspension of 0.3 µm and 0.05 µm alumina on felt polishing pads. The electrode was then rinsed thoroughly with deionized water and dried. The electrochemical cell was purged with acetonitrile, saturated with nitrogen, for at least 30 min to remove residual air. To confirm a clean electrode surface, the cell was filled with electrolyte solution (Tetrabutylammonium hexafluorophosphate ($TBAPF_6$), 0.1 mol/L) to record a background scan. Afterwards the cell was filled with 1.5 mL of sample solution. A flow of acetonitrile saturated argon over the electrolyte solution was maintained during all measurements. The measurements were conducted at room temperature.

1.5 UV-Vis spectroelectrochemistry

UV-Vis-spectroelectrochemical measurements were conducted under nitrogen atmosphere in a glovebox. The cell was a 0.2 mm spectroelectrochemistry cuvette, equipped with a platinum honeycomb working electrode with incorporated platinum counter electrode (Pine Research Instrumentation) and a coated silver wire as Ag/AgCl pseudo reference electrode. Potentials were applied with an Autolab PGSTAT204 potentiostat (Methrom) and the corresponding software Nova 2.1 (Metrohm, Germany). Prior to use the working and counter electrode were electrochemically cleaned by performing voltametric cycles in sulfuric acid (0.5 M) and rinsing the electrodes with deionized water. Before the measurement the cell was filled with electrolyte solution (0.1 mol/L $TBAPF_6$/acetonitrile) and a cyclovoltammetry scan was performed to confirm clean electrode surface. After removal of the electrolyte solution, 0.5 mL of sample solution was filled in the cell. Absorption changes during the cyclic voltammetry experiments were collected with a multichannel diode array spectrometer (Avantes) and an Avalight-DH-S-BAL light source (deuterium and halogen lamp, Avantes), fiber-coupled into the cuvette holder.

1.6 UV-Vis spectroscopy

If not stated otherwise UV-Vis-spectra were recorded at room temperature on V-780 spectrometer (JASCO) or a Specord S600 spectrometer (Analytik Jena AG) in 10 mm quartz cuvettes. For background correction the solvent spectrum was subtracted.

1.7 UV-Vis irradiation experiments

For irradiation experiments samples were prepared under nitrogen atmosphere in a glovebox or with standard Schlenk technique with anhydrous and degassed solvents. Samples were directly prepared in 10 mm quartz cuvette with a vacuum tight teflon stopper, which was wrapped in aluminium foil to prevent premature exposure to light.

Two setups for irradiation experiments were used. In the first setup the sample was irradiated outside the spectrometer with a white light LED with an irradiance of 20 mW cm^{-2} (white light LED 1, ChiliTec GmbH, see Figure S39 for spectral characteristic). In the second setup the sample was irradiated inside the spectrometer in a custom-build sample holder with mounted LEDs from Thorlabs (white light LED 2, MNWHL4 with an irradiance of 11 mW cm^{-2}, other wavelengths see respective experiments, see Figure S39 for spectral characteristics). The irradiated area of the cuvette in this setup was 2.13 cm^2.

Reoxidation: As typical test for reversibility of the photoreduction, the reaction solution was reoxidised by opening the cuvette and allowing the solution to react with oxygen from the air.

Absorption time profile measurements: Two samples were prepared for irradiation of **2** (64 µM and 56 µM) with BIH (1.05 eq. and 1.33 eq.). Irradiation was performed with the automatic irradiation setup inside the spectrometer and in a two-wavelength recording mode. The cuvette was thermostated to 22°C. In the first experiment, the LED light source (455 nm, 5.17 mW cm^{-2}) continuously irradiated the sample. In the second measurement, the irradiation source was switched off when the intermediate reached maximum concentration.

1.8 NMR spectroscopy

^{1}H, ^{13}C{^{1}H}, ^{31}P and ^{19}F NMR were recorded on the following Bruker BioSpin spectrometers: Avance NEO 500 (500 MHz, ^{1}H; 125.7 MHz, ^{13}C) with a cryo-cooled BBFO Prodigy probe, an Avance NEO 300 (300 MHz, ^{1}H; 75. MHz, ^{13}C)with a BBFO iProbe and an Avance III (400.13 MHz, ^{1}H, 76.31 MHz, ^{13}C) with a BBO probe. Chemical shifts are reported in parts per million relative to tetramethylsilane (^{1}H, ^{13}C), 85% phosphoric acid (^{31}P) and trifluoromethane (^{19}F) as external standards. Spectra were referenced with the residual solvent signals. For NMR experiments under inert conditions, an NMR tube with a young valve (air-tight NMR tube) was used. Solutions were prepared and filled into the air-tight NMR tubes in a glovebox under nitrogen atmosphere.

^{1}H DOSY NMR: The ^{1}H DOSY NMR spectra were acquired on a Bruker Avance NEO 500 MHz spectrometer equipped with a broadband cryo-cooled probe (Prodigy). The double stimulated echo sequence with bipolar gradient pulses and three spoil gradients with convection compensation from Bruker (dstebpgp3s) was used.[5,6] The diffusion time was Δ = 0.035 s and the duration of the magnetic field pulsed gradients $\delta/2$ was 2000 µs. The delay for gradient

recovery was 0.2 ms and the eddy current delay set to 5 ms. For each experiment, a series of 32 spectra on 16 K data points were collected. The pulse gradients (*g*) were incremented from 5 to 95% of the maximum gradient strength in a linear ramp. After Fourier transformation and baseline correction, the diffusion dimension was processed with the Topspin 4.4 software.

1.9 Fs-TA spectroscopy

Experiments were performed in a similar setup as described elsewhere.[7–9]

The sample solutions were prepared under inert conditions using anhydrous and degassed acetonitrile. Solutions of **2** (130 µM) with different ratios of BIH (0 eq., 2 eq. and 30 eq.) were transferred to airtight 1 mm quartz cuvettes (optical density of **2** 0.25). The power of the pump pulse directly in front of the sample was 300 µW. The pump wavelength was 532 nm. During these measurements the cuvette was fixed in a sample holder connected to a computer-controlled stage setup which moved the cuvette in a rectangular pattern in the x, y-plane. The speed of the cuvette movement was 70 µm/s. To ensure reproducibility and to exclude the possibility of sample degradation, UV-Vis spectra were recorded before and after the TA experiments.

2 Absorption properties of 1 and 2

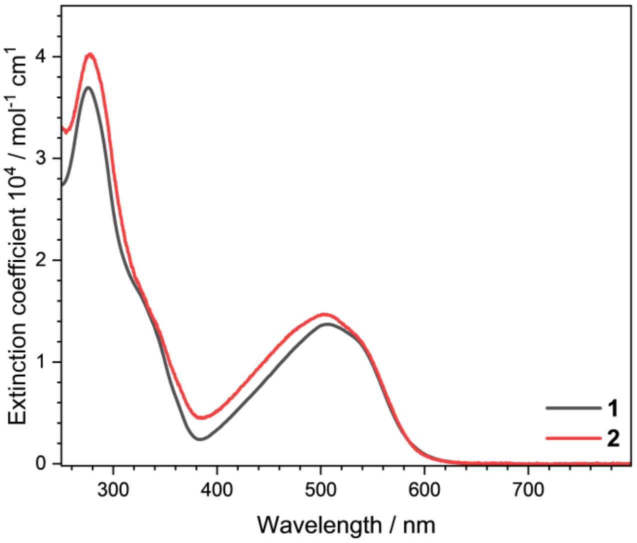

Figure S1. Absorption spectra of **1** and **2**, recorded in acetonitrile.[2,3]

3 Electrochemical properties of 1 and 2

The electrochemical properties of **1** and **2** were investigated by cyclic voltammetry and have been reported previously.[2,3] A comparison of the cyclovoltammetric data is shown in Figure S2. It has been reported before that the electronic effect of substitution pattern of the imidazolato ligand influences the electrochemical reduction of homoleptic Cu(I) 4*H*-imidazolato complexes.[10–12] In the case of **1** the electron withdrawing effect of two CF_3 substituents causes the shift of ca. 160 mV of the redox potentials to more positive values in comparison to **2**. Additionally, the chemical reversibility behavior of **1** indicates a higher stabilization of the negative charges for $\mathbf{1}^{2-}$ by the four CF_3 groups.

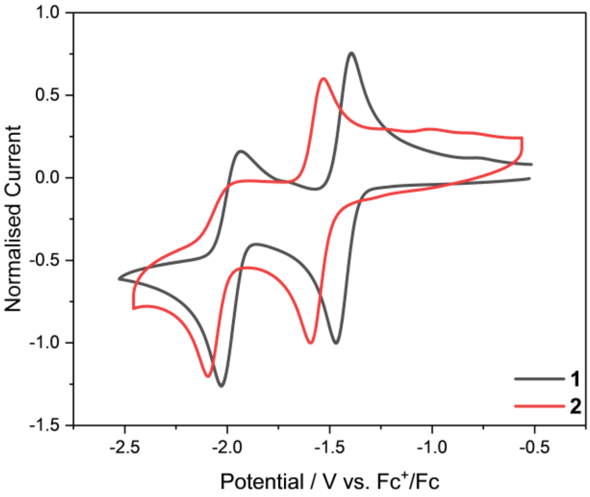

Figure S2. Cyclovoltammetry data for **1** (2 mM) and **2** (0.14 mM) in acetonitrile/$TBAPF_6$. The current is normalized based on the first reduction wave. **1** shows two one electron redox waves at –1.43 V ($\mathbf{1}/\mathbf{1}^{\bullet-}$) and –1.97 V ($\mathbf{1}^{\bullet-}/\mathbf{1}^{2-}$) vs Fc^+/Fc. The first chemical reversible wave shows that $\mathbf{1}^{\bullet-}$ is stable on the voltammetric time scale. The current for the backward scan of the second redox wave is smaller, indicating smaller stability for $\mathbf{1}^{2-}$ leading to follow up chemical reactions.[2] The cyclic voltammogram of **2** largely resembles the one of **1**. The potentials for the redox processes are slightly shifted to more negative values, –1.59 V ($\mathbf{2}/\mathbf{2}^{\bullet-}$) and -2.19 V ($\mathbf{2}^{\bullet-}/\mathbf{2}^{2-}$) vs Fc^+/Fc, in comparison to **1**.[3]

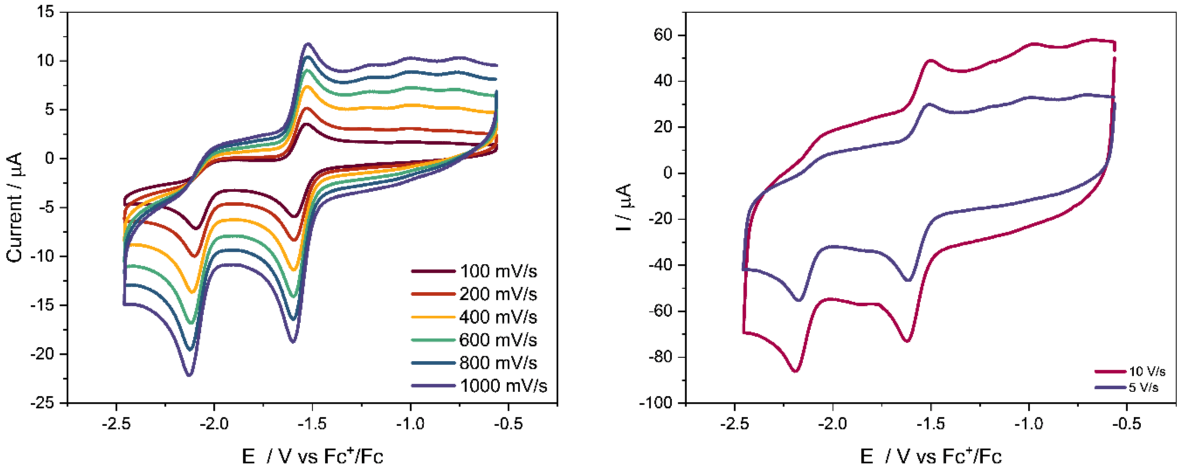

Figure S3. cyclic voltammograms of **2** (0.14 mM) in acetonitrile/0.1M $TBAPF_6$ at different scan rates. The voltammogram shows two redox waves at –1.59 V and –2.03 V vs. Fc^+/Fc. The peak-peak separation for the first process is 65 mV and linear increase of the peak current with the square root of the scan rate suggests an electrochemically reversible one-electron redox process. The two redox processes can be assigned to $\mathbf{2}/\mathbf{2}^{\bullet-}$ (–1.59 V) and $\mathbf{2}^{\bullet-}/\mathbf{2}^{2-}$ (–2.03 V). The second process is chemically irreversible, even at high scan rates.

4 Estimation of the excited state redox potential

The estimation of the excited state reduction potential can be performed using the Rehm-Weller equation (Equation 1) with E_{red} being the reduction potential in the ground state and E^{*}_{red} the reduction potential in the excited state and E_{00} the energy difference between the first vibrational levels of the ground state and lowest excited state.[13] For complexes that do not show emission, E_{00} can be estimated using the red tail of the visible absorption band[14,15], which is around 610 nm (2.03 eV) for complexes **1** and **2**. Calculated energies of the lowest excited triplet states of a similar Cu(I)-4*H*-imidazolato complex showed, that these values might be overestimated by 0.5 eV.[11]

$$E^{*}_{red} = E_{red} + E_{0,0} \quad (1)$$

$$E^{*}_{1/1^{\bullet-}} = E_{1/1^{\bullet-}} + E_{0,0} = -1.43\ \mathrm{V} + 2.03\ \mathrm{V} = 0.60\ \mathrm{V}$$

$$E^{*}_{2/2^{\bullet-}} = E_{2/2^{\bullet-}} + E_{0,0} = -1.59\ \mathrm{V} + 2.03\ \mathrm{V} = 0.44\ \mathrm{V}$$

5 Spectroelectrochemical properties of 1 and 2

Spectroelectrochemical investigations of **1** have been published before.[2] The one-electron reduction product $\mathbf{1}^{\bullet-}$ shows broad absorption features. It has characteristic bands in the red to NIR region (631 nm and 698 nm). This observation agrees with the formation of a 4*H*-imidazolato ligand based radical anion. Scanning over the second reduction wave results in a decrease in absorption in the visible region with a bleach around 530 nm and an increase in the absorption band at 390 nm. Backward scanning after the second reduction wave leads to spectral features of $\mathbf{1}^{\bullet-}$. The features decrease further while scanning with almost no absorption difference, compared to the initial spectrum, at the end point of the measurement.

2 shows very similar spectroelectrochemical behavior. The same characteristic radical absorption bands for $\mathbf{2}^{\bullet-}$ are formed when scanning over the first reduction wave, but their positions are slightly shifted in comparison to $\mathbf{1}^{\bullet-}$ (642 nm and 704 nm). Scanning backwards (not shown) leads to a decrease of the radical absorption features. Almost no absorption differences at the end point of the scan indicate a reversible reduction process.

On scanning over the second reduction wave, the radical absorption features decrease, leaving the features with a bleach minimum at 539 nm and an increased absorption at 374 nm. This can be attributed to the formation of the doubly reduced species $\mathbf{2}^{2-}$ or side products from following chemical reactions. Scanning backwards (not shown), at sufficiently negative potentials to generate the radical, results in an increase in the radical absorption features. Continued scanning to more positive potentials results in a decrease of these features, leaving almost no absorption differences, compared to the initial spectrum, at the end point.

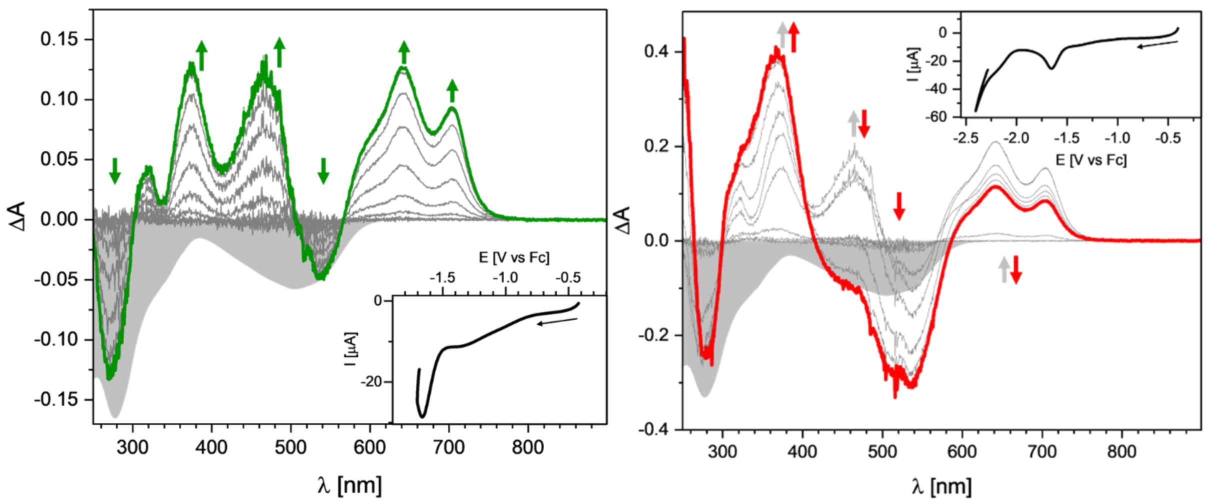

Figure S4: Spectroelectrochemical data for **2** (0.25 mM) in acetonitrile/0.1M $TBAPF_6$. Scanning over the first reduction wave (left) and scanning over first and second reduction wave (right). Spectra were recorded simultaneously with the run of the depicted cyclic voltammogram at 30 mV/s.

In contrast to the cyclic voltammetry experiments, where data were collected at a glassy carbon electrode[3] spectroelectrochemical data for **2** were collected at a honeycomb Pt-working electrode. The different electrode material only caused minor changes in the cyclic voltammogram. In addition to the reversible first reduction process and the second irreversible reduction process, there is an increasing current at the end of the potential window of unknown origin. However, the influence of this process can be considered small since the spectral characteristics during the scan of the second reduction wave did not differ significantly from those of **1**.[2]

6 Photoreduction experiments

6.1 Photoreduction and reoxidation of 1 using DMT (100 eq.) as electron donor and irradiation with different wavelength

The wavelength-dependent photoreduction reactivity of **1** in the presence of excess DMT was studied by in-situ UV-Vis spectroscopy under irradiation with 365, 455, 505, 660 and 970 nm LEDs (Figure S5–Figure S9). Upon 970 nm excitation, expectedly, no photoreduction was observed since **1** does not absorb at this wavelength. In contrast, at wavelengths of 365, 455, and 505 nm, intense photoreduction processes were observed. At these wavelengths, there was a noticeable decrease of the initial visible absorption band, indicating the photoreduction to the colorless two-electron reduced species and a significant conversion was observed after several hours (see Table S. Even at 660 nm, where the tailing LED spectrum has small overlap with the absorption red edge, still 39% conversion was noted after 18 hours of irradiation (

Figure S8). Under these photoreduction conditions and at either irradiation wavelengths the spectral changes are very similar and suggest the formation of the same photoreduction product without noticeable side reactions or an intermediate species. Additionally, the photoreduction processes are reversible upon exposure to atmospheric oxygen.

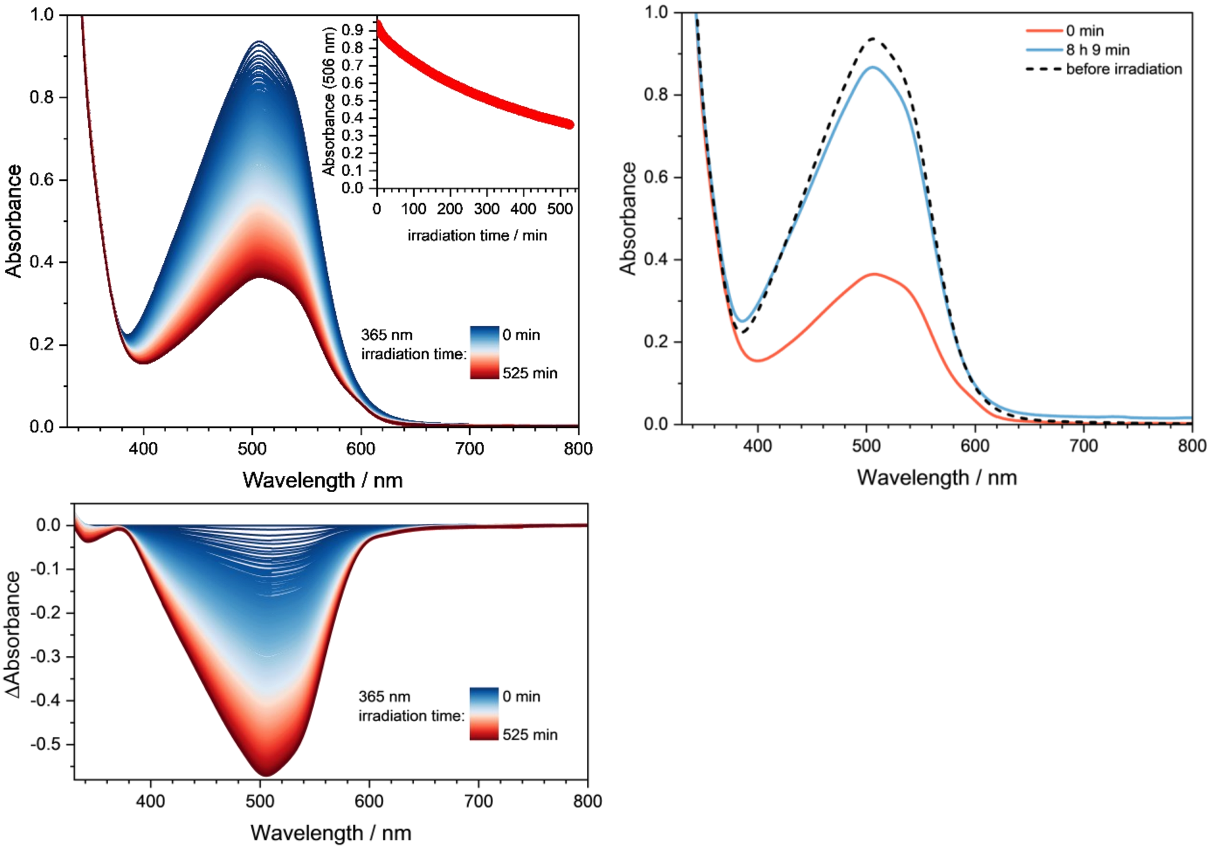

Figure S5. **Photoreduction of 1 (60 µM; 10 mm optical pathlength) with 365 nm LED** irradiation in acetonitrile (upper left) and corresponding absorption difference spectra (lower left) and reoxidation (right). In acetonitrile, **1** exhibits a broad absorption band from 380–660 nm, with a maximum at 506 nm. Upon irradiation, the absorbance at this maximum decreased from 0.934 to 0.362 after 525 min irradiation time, corresponding to a reduction of **1** to 38% of its initial concentration. The band shape remained consistent during this process, indicating no major changes in electronic structure apart from the reduction event. After reoxidation with oxygen the absorbance at the 506 nm maximum increased from 0.362 to 0.867 (93% recovery).

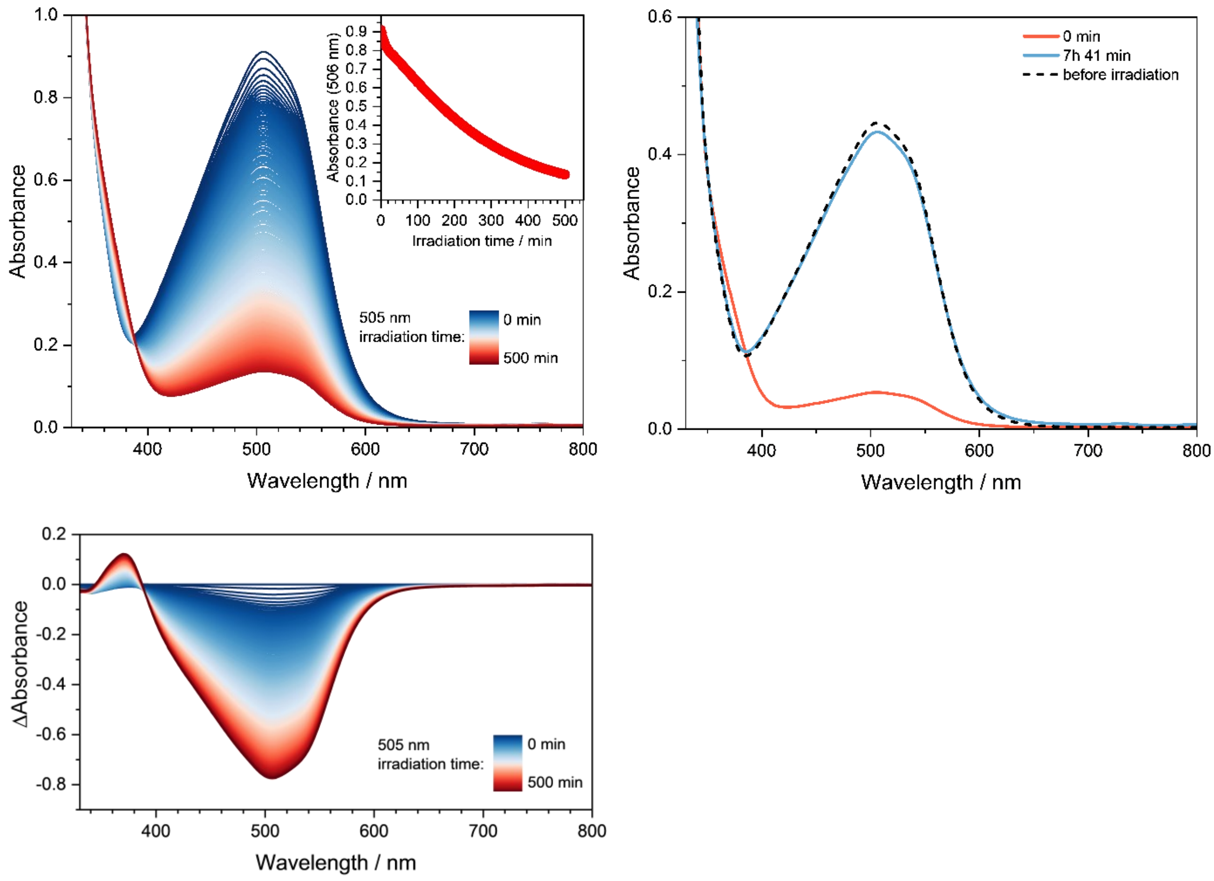

Figure S6. **Photoreduction of 1 (60 µM; 10 mm optical pathlength) with 505 nm LED** irradiation in acetonitrile (upper left) and corresponding absorption difference spectra (lower left) and reoxidation (right). Upon irradiation, the absorbance at the maximum decreased from 0.904 to 0.131 after 500 min irradiation time, corresponding to a reduction of **1** to 14% of its initial concentration. The band shape remained consistent during this process, indicating no major changes in electronic structure apart from the reduction event. The shown reoxidation was carried out after 550 min of irradiation of a solution with smaller initial concentration of **1** (30 µM). After reoxidation with oxygen the absorbance of at the 506 nm maximum increased from 0.054 to 0.433 (97% recovery).

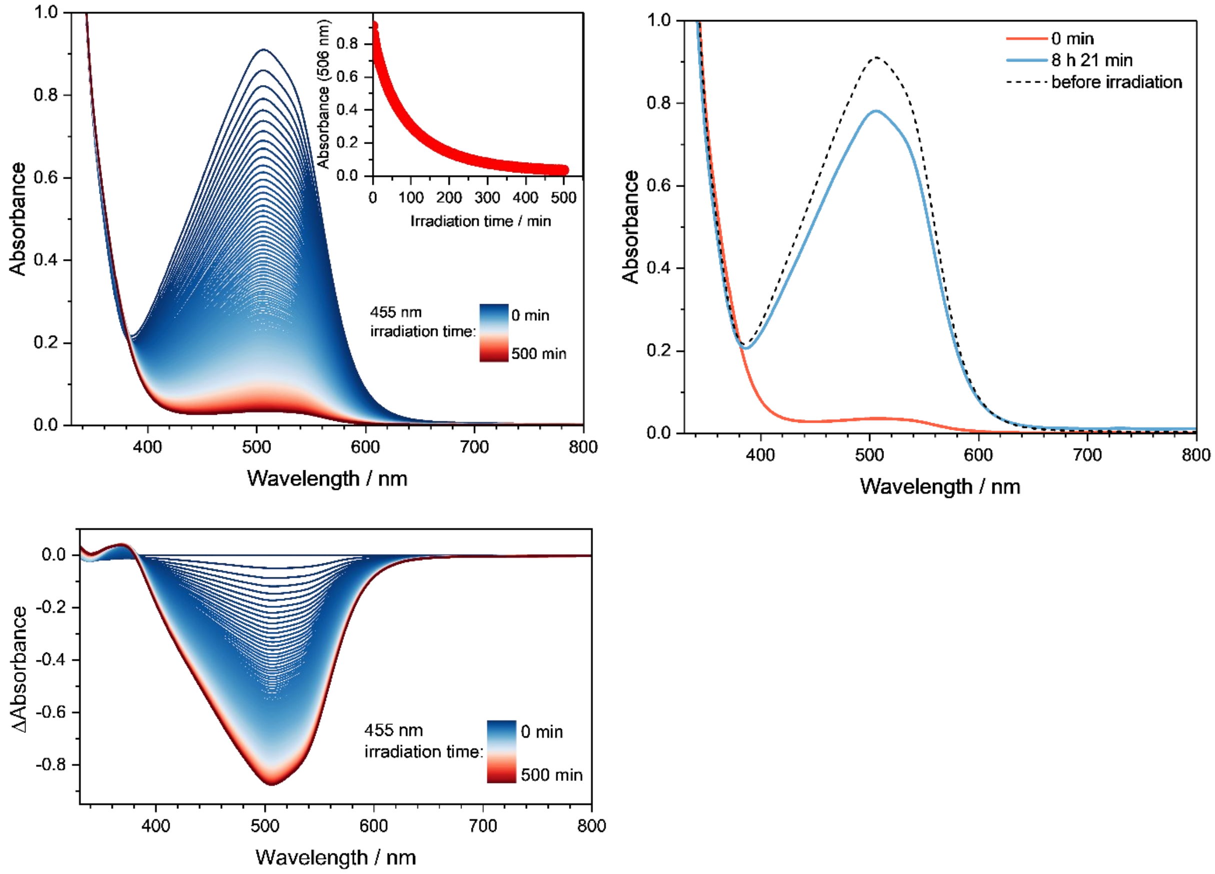

Figure S7. **Photoreduction of 1 (60 µM; 10 mm optical pathlength) with 455 nm LED** irradiation in acetonitrile (upper left) and absorption difference spectra (lower left) and reoxidation (right). Upon irradiation, the absorbance at the maximum decreased from 0.909 to 0.036 after 500 min irradiation time, corresponding to a reduction of **1** to 4% of its initial concentration. The band shape remained consistent during this process, indicating no major changes in electronic structure apart from the reduction event. After reoxidation with oxygen the absorbance of at the 506 nm maximum increased from 0.036 to 0.781 (86% recovery).

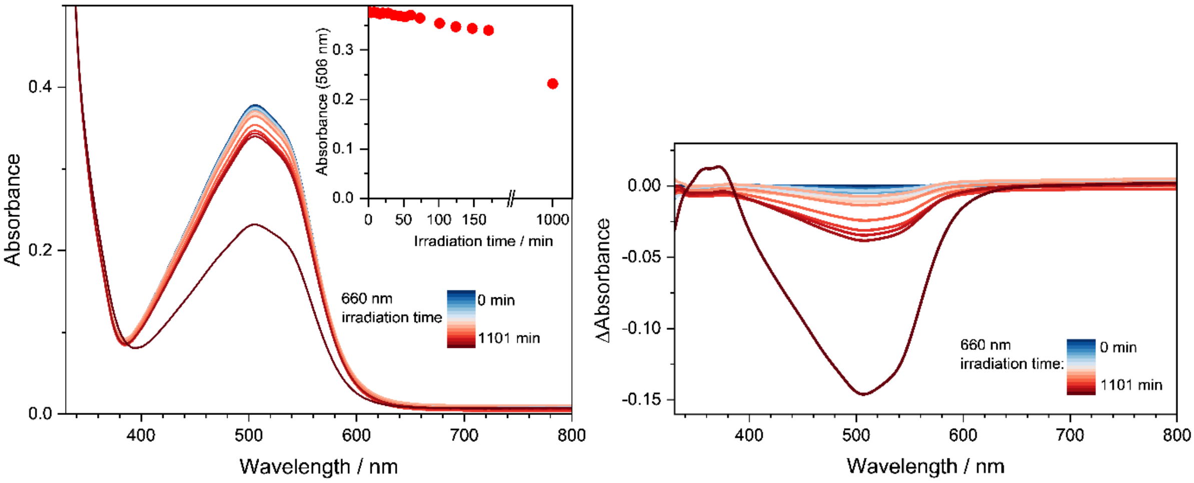

Figure S8: Photoreduction of **1 (240 µM; 1 mm optical pathlength) with 660 nm LED** irradiation in acetonitrile (left) and corresponding absorption difference spectra (right). Upon irradiation, the absorbance at the maximum decreased from 0.378 to 0.232 after 1101 min irradiation time, corresponding to a reduction of **1** to 61% of its initial concentration. The band shape remained consistent during this process, indicating no major changes in electronic structure apart from the reduction event.

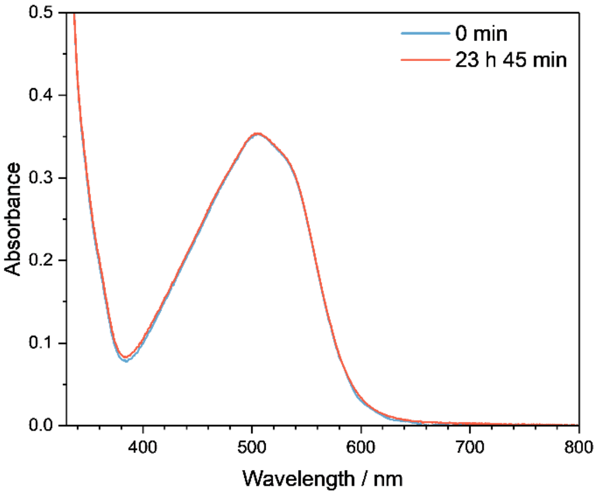

Figure S9: **Photoreduction of 1 (240 µM; 1 mm optical pathlength) with 970 nm LED** irradiation in acetonitrile Upon irradiation no changes of the absorption could be observed even after 23 h 45 min irradiation time.

Table S1: Summary of Photoreduction reoxidation experiments of **1** using 100 eq. of DMT as electron donor in acetonitrile, under irradiation with different wavelengths.

λ_{irr} / nm	t_{irr}	conversion $(1 - A_{irr}/A_{initial})$	recovery $(1 - A_{reox}/A_{initial})$
365	525 min	62%	93%
455	500 min	96%	86%
505	500 min	86%	97%
660	1101 min	39%	-
790	1 d	0%	-

6.2 Photoreduction of 1 in different solvents using DMT (100 eq.) as electron donor under white light irradiation

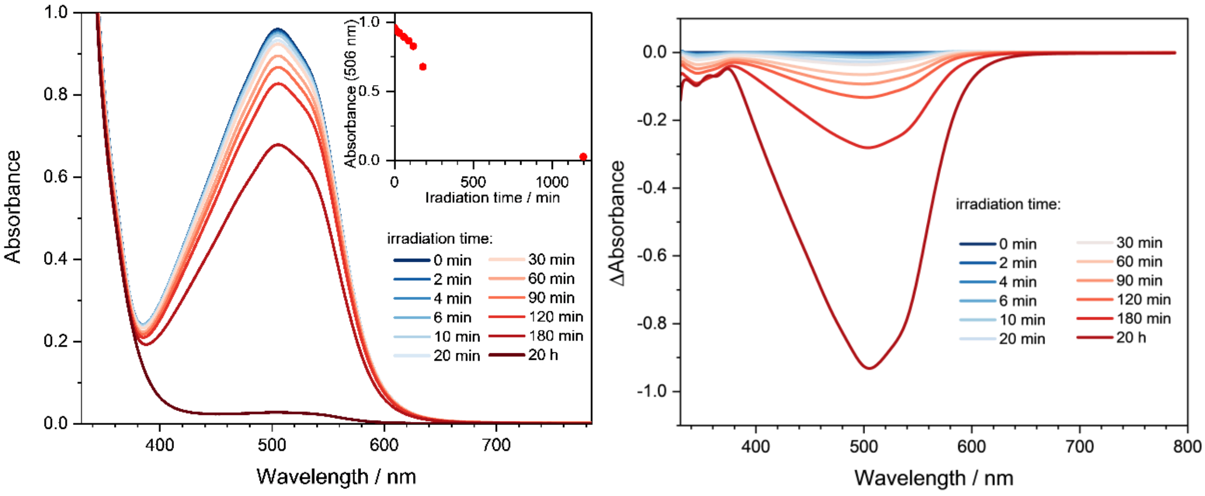

Figure S10. **Photoreduction of 1 (80 µM) in acetonitrile** (left) and corresponding absorption difference spectra (right). During irradiation with **LED white light** (LED 1), the absorbance at this maximum decreased from 0.960 to 0.028 after 20 hours, corresponding to a reduction of **1** to 3% of its initial concentration. The band shape remained unchanged during this process, indicating no major changes in electronic structure apart from the reduction event.

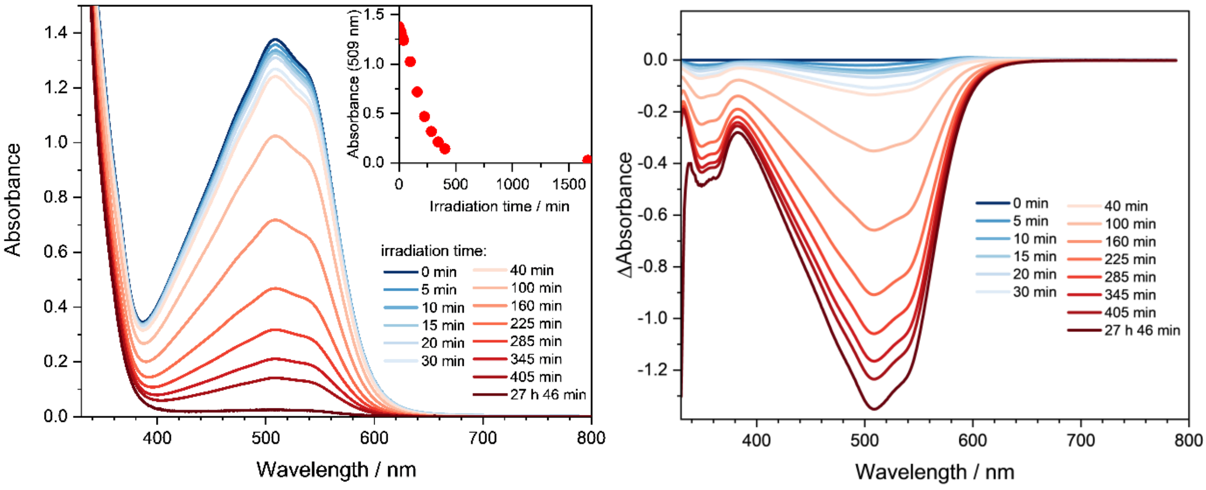

Figure S11. **Photoreduction of 1 (80 µM) in DME** (left) and corresponding absorption difference spectra (right). In DME, **1** displayed a similar absorption profile as in acetonitrile, with the maximum slightly shifted to 509 nm (bathochromic shift of 0.014 eV). Initial absorbance at this maximum was higher than in acetonitrile, indicating solvent-specific effects on the electronic environment (cf. reference 16). After 28 hours of irradiation with **LED white light** (LED 1), the absorbance decreased from 1.378 to 0.010, leaving 1% of the original complex intact. Despite the slower kinetics compared to acetonitrile, the band shape remained unchanged.

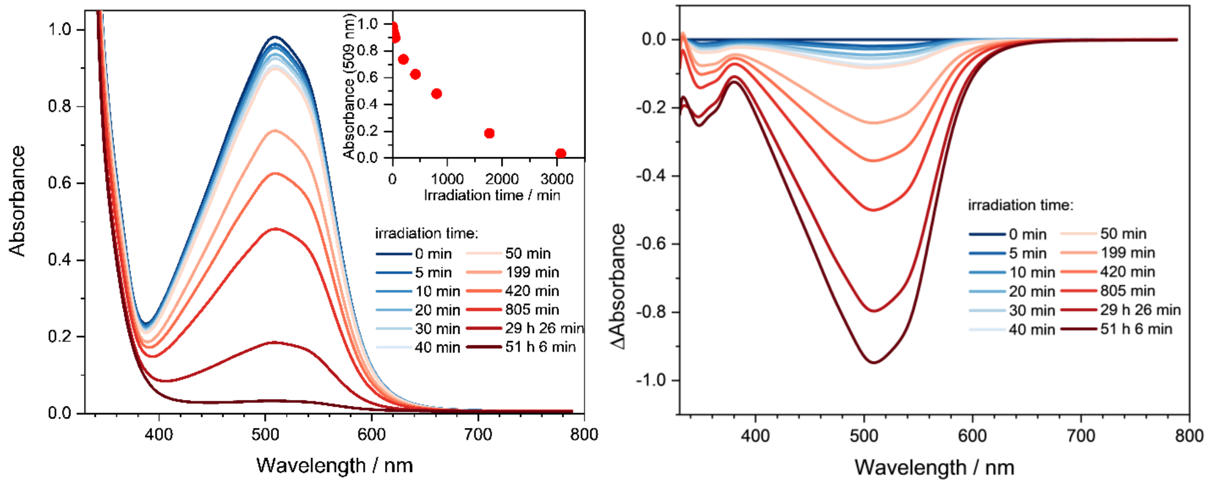

Figure S12. **Photoreduction of 1 (80 µM) in PC** (left) and corresponding absorption difference spectra (right). **1** in PC showed similar initial spectral characteristics in comparison to DME, with a maximum at 509 nm. The reduction process was slower due to PC's higher viscosity in comparison to acetonitrile and DME. After 51 hours of irradiation with **LED white light** (LED 1), the absorbance decreased from 0.981 to 0.033, corresponding to 3% of the initial concentration. Interestingly, prolonged irradiation resulted in a slight band broadening, possibly due to intermolecular interactions or the formation of secondary species.

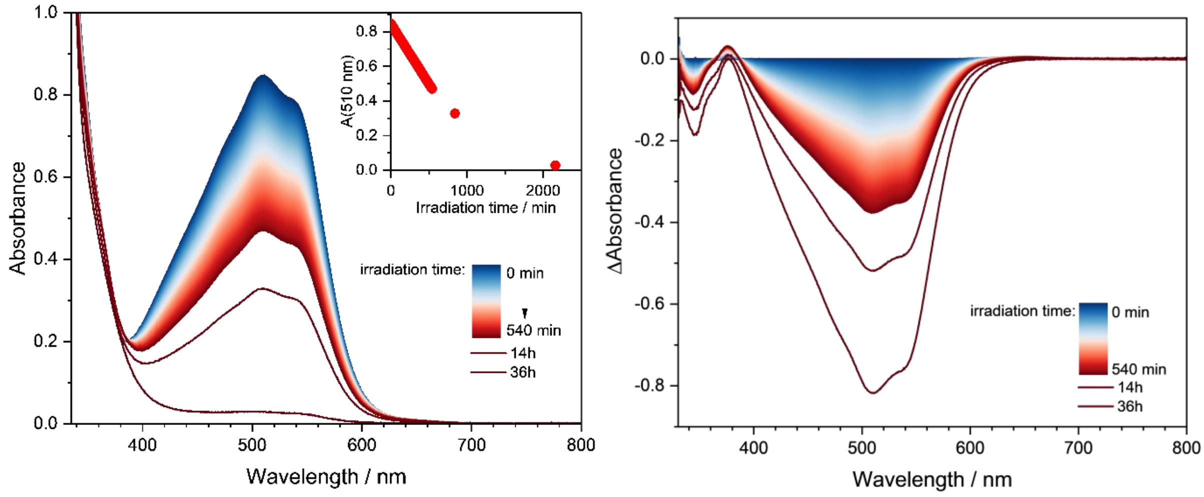

Figure 13 **Photoreduction of 1 (80 µM) in THF** (left) and corresponding absorption difference spectra (right). **1** in THF shows similar initial spectral characteristics with a maximum at 510 nm. Upon irradiation with **LED white light** (LED 2), the absorbance at the maximum decreased from 0.846 to 0.029 after 36 hours, corresponding to a reduction of **1** to 3% of its initial concentration. The band shape remained unchanged during this process.

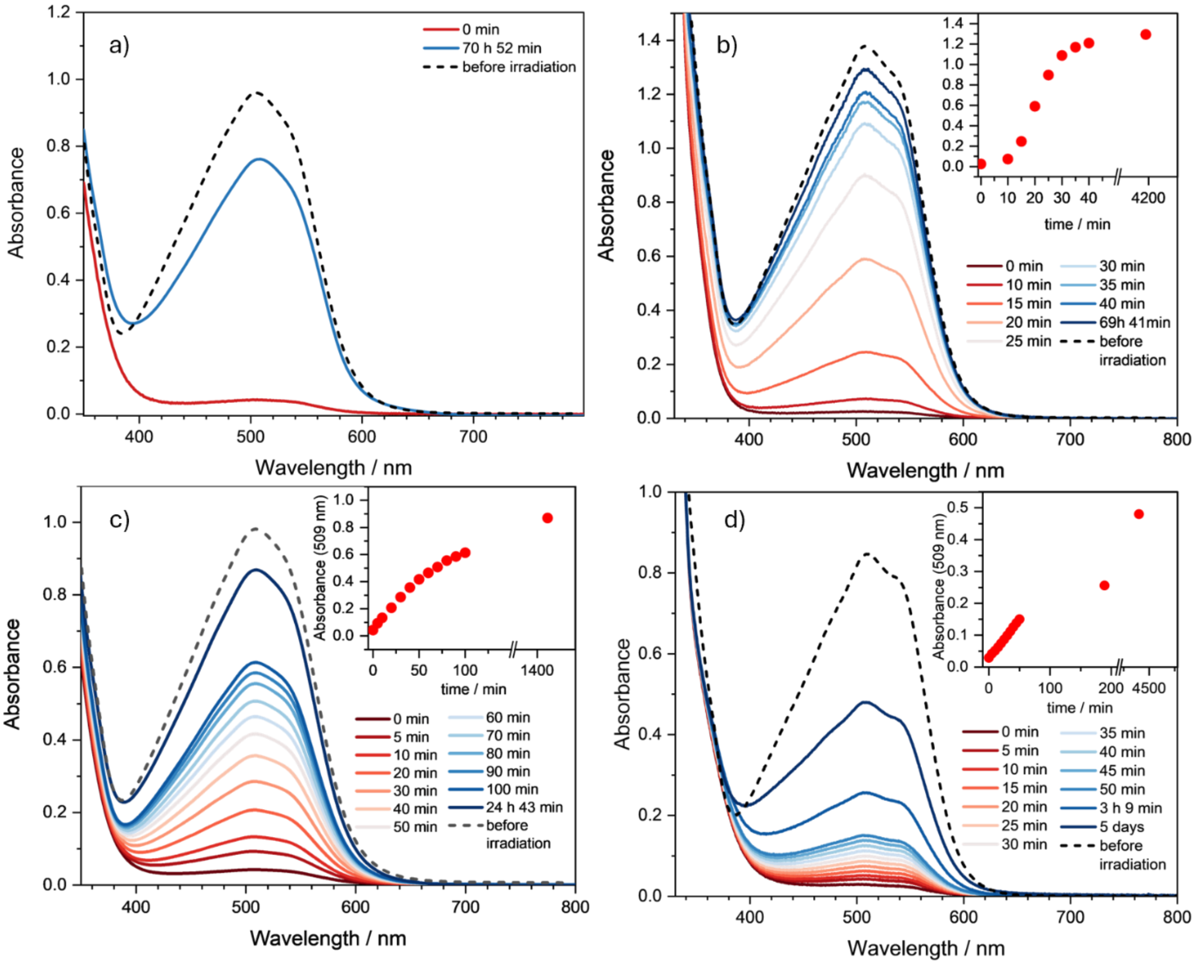

Figure S14. **Reoxidation with oxygen after photoreduction in acetonitrile, DME, PC and THF.** The extent of reoxidation varied across solvents: a) acetonitrile: Absorbance at the 506 nm maximum increased from 0.028 to 0.761 (79% recovery) after 70 hours and 52 min. b) DME: Absorbance at the 509 nm maximum increased from 0.010 to 1.29 (94% recovery) after 69 hours and 41 min. c) PC: Absorbance at the 509 nm maximum increased from 0.033 to 0.869 (89% recovery) after 24 hours and 43 min. d) THF: Absorbance at the 509 nm maximum increased from 0.029 to 0.480 (58% recovery) after 5 days.

Table S2: Summary of Photoreduction reoxidation experiments of **1** using 100 eq. of DMT as electron donor in different solvents under white light irradiation.

solvent	P_{irr} / mWcm^{-2}	t_{irr}	conversion	t_{reox}	recovery
Acetonitrile		20 h	97%	71 h	79%
DME	20	28 h	99%	70 h	94%
PC		51 h	97%	25 h	89%
THF	11	36 h	97%	5 d	57%

6.3 Photoreduction of 1 using different electron donors in acetonitrile

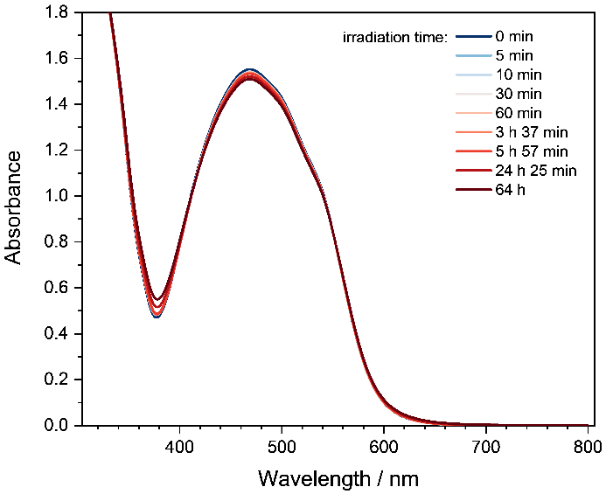

Figure S15. **Photoreduction of 1 (80 µM) with ferrocene (100 eq.) as electron donor in acetonitrile.** Even after 64 hours under **LED white light** (LED 1) irradiation only minor absorbance changes occurred.

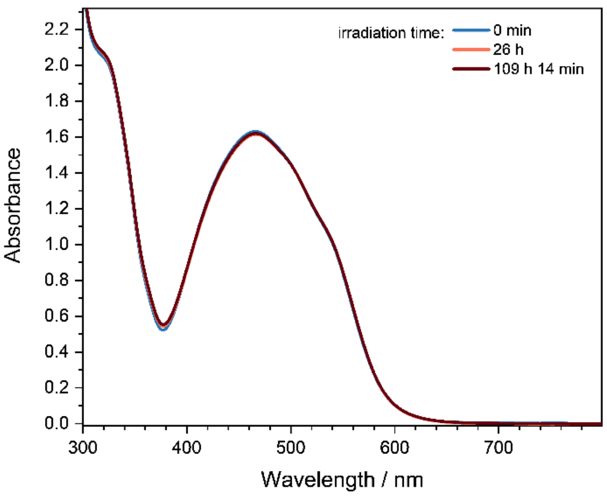

Figure S16. **Photoreduction of 1 (80 µM) with ferrocene (100 eq.) as electron donor in presence of $TBAPF_6$ in acetonitrile.** Even after 109 hours under **LED white light** (LED 1) irradiation only minor absorbance changes occurred.

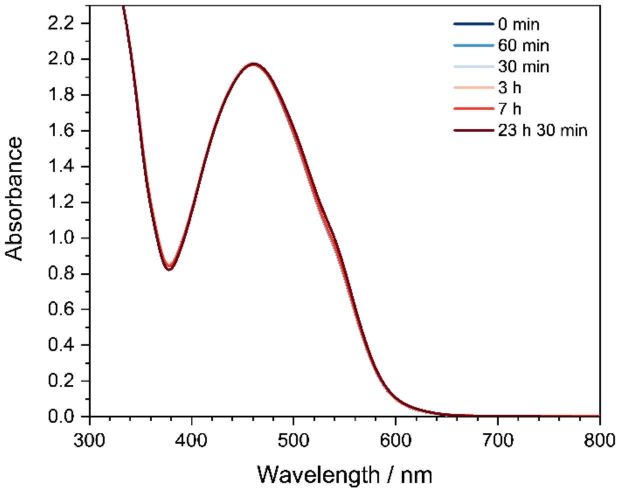

Figure S17. **Photoreduction of 1 (80 µmol) with ferrocene (100 eq.) as electron donor in presence of NH_4PF_6 in acetonitrile.** After an irradiation time of 7 hours with **LED white light** (LED 1) the sample was heated to from 297 K to 313 K. Even after 23 hours of white light irradiation only minor changes of the absorbance occurred.

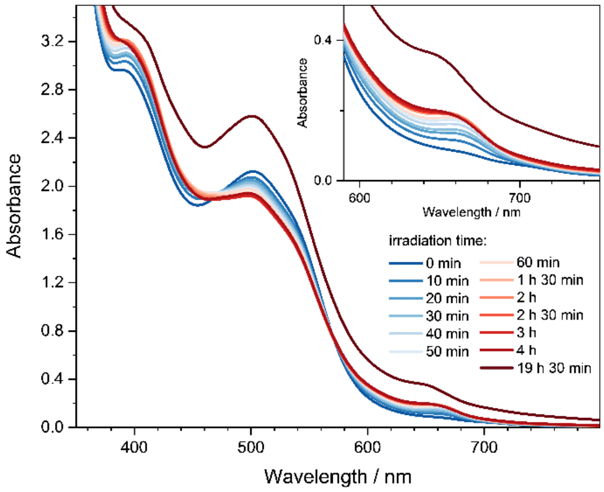

Figure S18. **Photoreduction of 1 (80 µM) with cobaltocene (100 eq.) as electron donor in acetonitrile.** The spectral changes upon **LED white light** (LED 1) irradiation could not be assigned to the one electron-reduced or two electron reduced species and indicate the occurrence of side reactions.

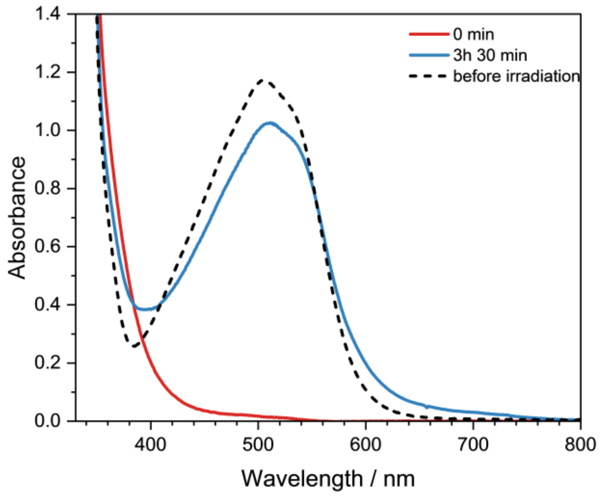

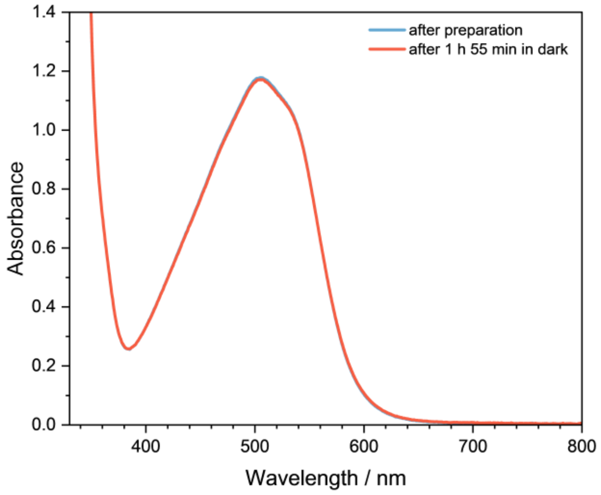

Figure S19. **Photoreaction of 1 (80 µM) with BIH (100 eq.) as electron donor and reoxidation in acetonitrile (see Figure 2, main text).** After reoxidation with oxygen the absorption maximum increased from 0.015 to 1.021 (87% recovery) and the maximum shifted to 511 nm while the spectrum is broadened in the NIR region between 600 nm and 780 nm. Additionally, absorbance increased around 400 nm, probably due to formation of side products (upper panel). To exclude ground state reactions of **1** with BIH the solution was kept in the dark for 2 hours prior to irradiation. Small spectral changes are observable due to exposure to room light while placing the sample in the spectrometer (lower panel).

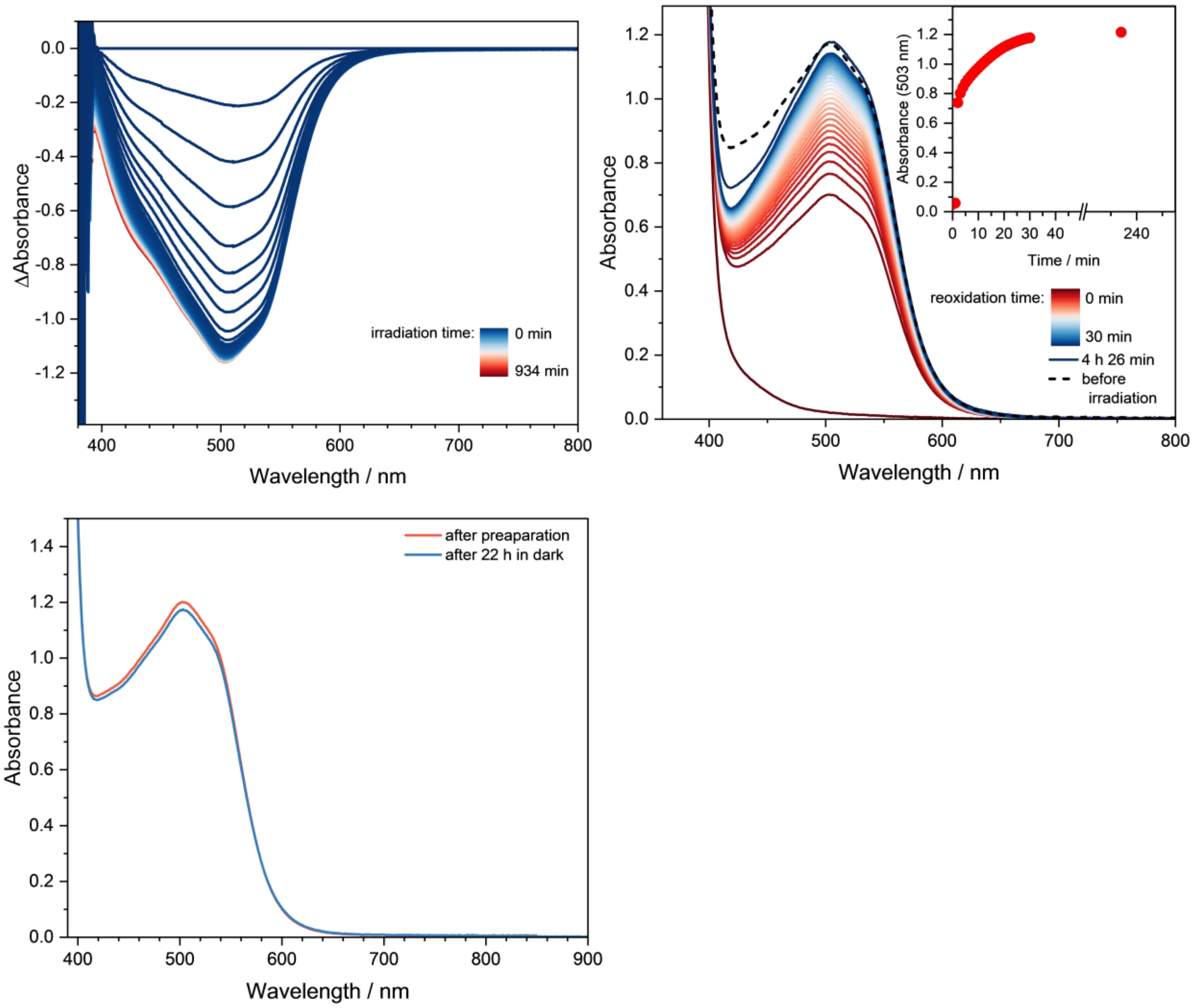

Figure S20. **Photoreaction of 1 (80 µM) with BNAH (100 eq.) as electron donor and reoxidation in acetonitrile (see Figure 2, main text).** Differential absorption spectra of the photoreduction (upper left). After reoxidation with oxygen Absorption at the maximum increased from 0.014 to 1.172 (100 % recovery) (upper right). To exclude ground state reactions of **1** with BNAH the solution was kept in the dark for 22 hours prior to irradiation with only minor observable spectral changes, probably due to exposure to room light while placed in the spectrometer (lower left).

6.4 Photoreduction of 2 using different electron donors in acetonitrile

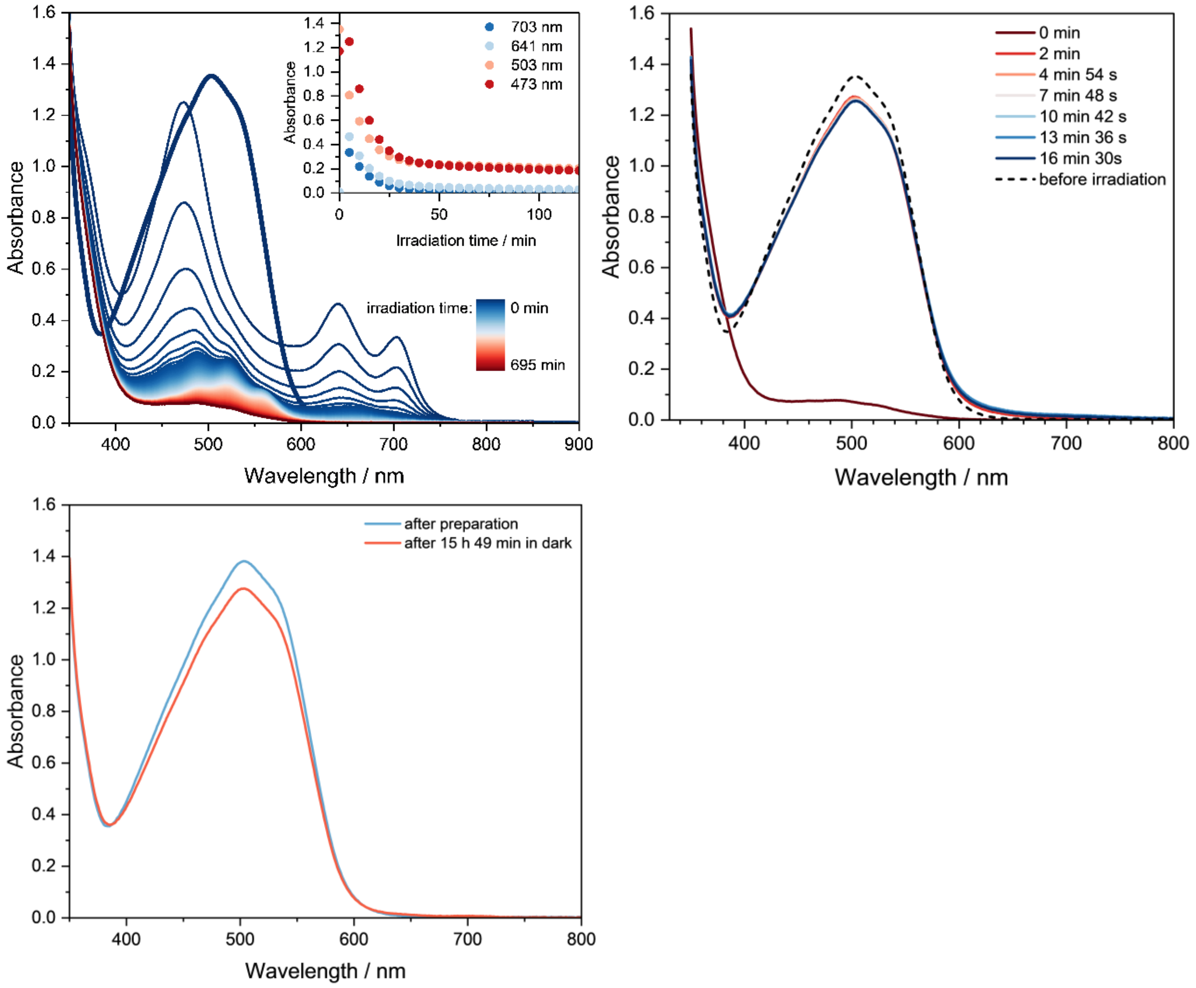

Figure S21. **Photoreaction of 2 (80 µM) with BIH (100 eq.) as electron donor (upper left) and reoxidation (upper right) in acetonitrile**. Upon **LED white light** (LED 2) irradiation, the absorbance at the maximum decreased from 1.352 to 0.070 after 695 min irradiation time, corresponding to a reduction of **2** to 5% of its initial concentration. During photoreduction the spectral changes are similar to the photoreduction of **1** with BIH under same reaction conditions, with new maxima forming at 473 nm, 641 nm and 703 nm. While the new features decreased with ongoing irradiation a structured band between 450 nm and 600 nm appeared with maxima at 490 nm and 520 nm and two shoulders at 558 nm and 460 nm. With ongoing irradiation, the absorbance of these new features decreased as well, to form the colorless reduction product. After reoxidation with oxygen Absorption at the maximum increased from 0.070 to 1.256 (93% recovery). After reoxidation, the visible absorption band is slightly broadened between 600 and 700 nm. To exclude ground state reactions of **2** with BIH an equally prepared solution was kept in the dark for 16 hours prior to irradiation with considerably smaller observable spectral changes than for the irradiation experiment, probably due to exposure to room light while placed in the spectrometer (lower left).

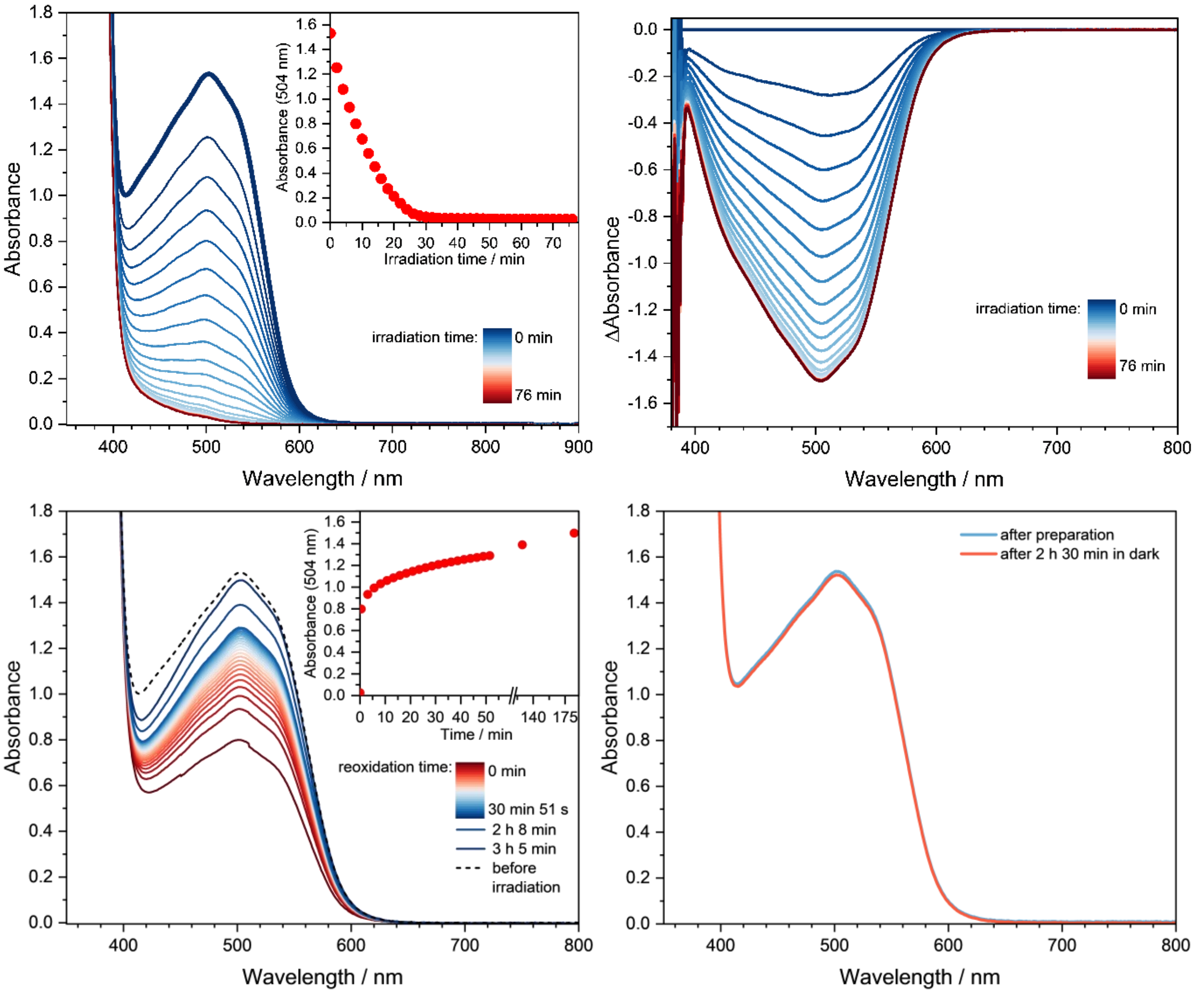

Figure S22: **Photoreaction of 2 (80 µM) with BNAH (100 eq.) as electron donor (upper left) and reoxidation (lower left) in acetonitrile.** The absorption spectrum is slightly changed due to the presence of BNAH in solution (broadening of the absorption band between 400 nm and 500 nm. Upon **LED white light** (LED 2) irradiation, the absorbance at the maximum decreased from 1.531 to 0.026 after 76 min irradiation time, corresponding to a reduction of **2** to 1.7% of its initial concentration (corresponding differential spectra upper right) After reoxidation with oxygen Absorption at the maximum increased from 0.026 to 1.497 (98 % recovery). To exclude ground state reactions of **2** with BNAH an identical sample was kept in the dark for 2.5 hours with only minor observable spectral changes, probably due to exposure to room light while placed in the spectrometer (lower left).

Table S3: Summary of Photoreduction reoxidation experiments of **1** and **2** using 100 eq. of either BIH or BNAH as electron donor in acetonitrile under white light irradiation with 11 mW cm^{-2}.

Complex	electron donor	t_{irr} / min	conversion	recovery
**1**	BIH (100 eq.)	710	99%	87%
	BNAH (100 eq.)	934	99%	100%
**2**	BIH (100 eq.)	695	95%	93%
	BNAH (100 eq.)	76	98%	98%

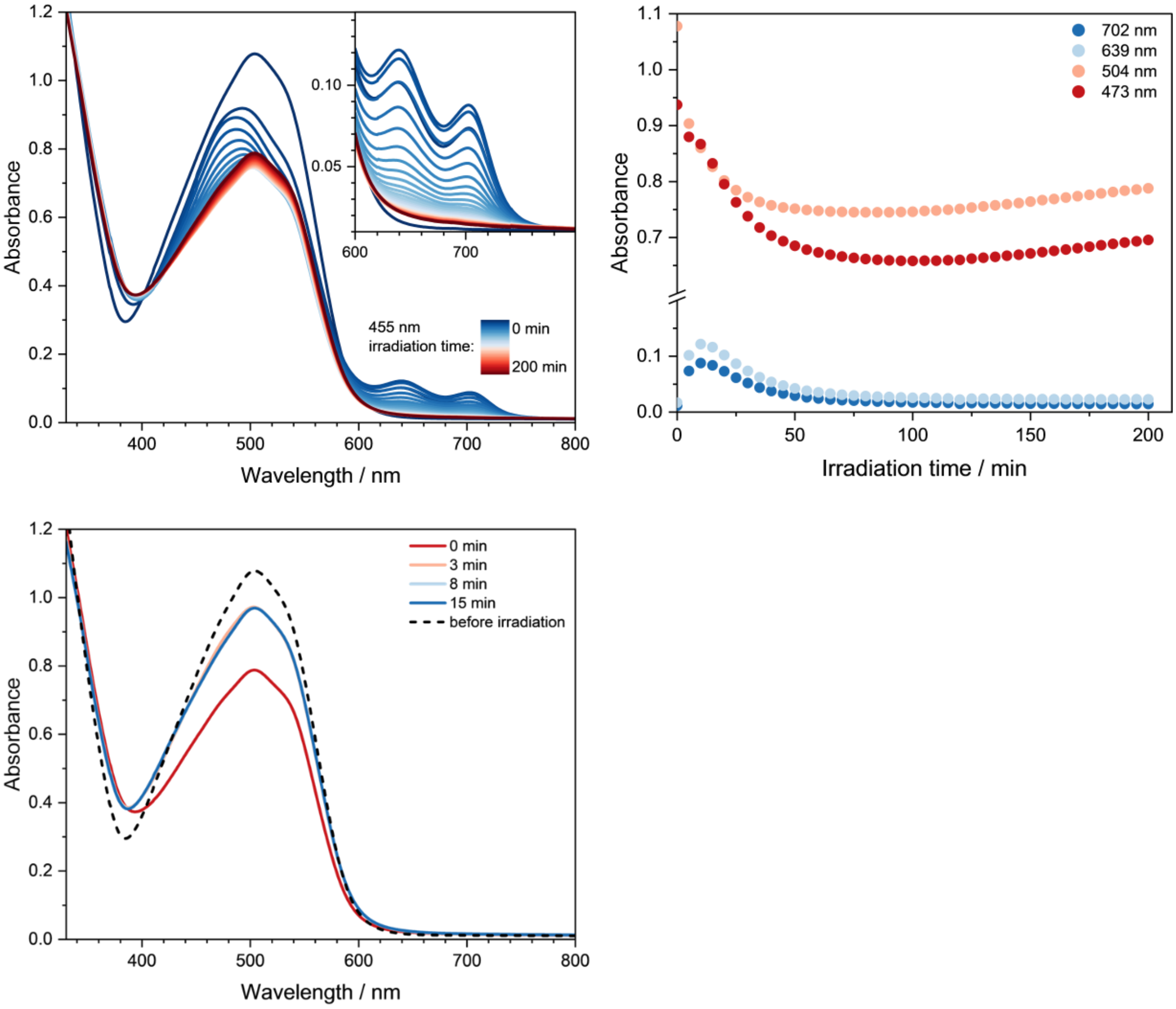

Figure S23. **Photoreaction of 2 (64.5 µM) with BIH (1 eq.) as electron donor in acetonitrile.** Upon irradiation with **455 nm LED** light source in presence of equimolar amount of BIH, the absorbance at the maximum decreased from 1.078 to 0.745 after 85 min irradiation time, corresponding to a reduction of **2** to 69% of its initial concentration (upper left). During photoreduction the spectral changes are similar to the photoreduction of **2** with 100 eq. BIH under white light irradiation: while the maximum is shifted to shorter wavelengths two additional bands appear at 640 nm and 704 nm. After 85 min irradiation time the visible absorption spectrum equals the spectrum of **2** with slightly broadening between 600 nm and 700 nm. With ongoing irradiation, the absorption at the 504 nm maximum increases to 0.788. After reoxidation with oxygen Absorption at the maximum increased from 0.788 to 0.969 (90 % recovery, lower left).

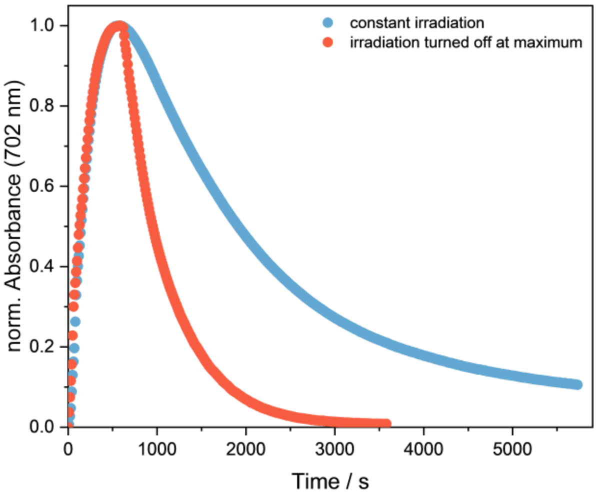

Figure S24. **Absorption-time-profile for the radical intermediate in the photoreduction of 2 (60 µM) with BIH (1 eq.) in acetonitrile**. Normalized absorption time profile of the 702 nm band under constant irradiation and when the light source was switched off at maximum intermediate concentration (left). The absorption decreases much faster after the light source was switched off, because no new intermediate was formed, and the intermediate reacts in a light-independent reaction.

7 Single-crystal X-Ray structural analysis of 1 and the photoreduction product $^{exo}H_2$**2**

The single-crystal X-ray intensity data for **1** and $^{exo}H_2$**2** were collected on a Bruker-Nonius KappaCCD diffractometer with a Mo-Kα microfocus source. The crystal structure was solved with SHELXT-2018/3[17] and refined by full matrix least-squares methods on F^2 with SHELXL-2018/3[18], using the Olex2 environment.[19] Multi-scan absorption correction was applied to the intensity data.[20] The H atoms attached to C atoms were geometrically fixed, whereas the N-bound H atoms were located in the difference Fourier map. Restrains on the corresponding N-H bond lengths were applied (DFIX command in ShelXL).[18]

Table S4: Crystal data and refinement details for compound $^{exo}H_2$**2**.

	$^{exo}H_2$**2**	**1**
CCDC deposition Nr.	2498825	2502609
Empirical formula	$C_{66}H_{55}CuF_6N_4O_2P_2$	$C_{67}H_{49}CuF_{12}N_4O_2P_2$
Formula weight	1175.62	1295.58
Temperature/K	120	133(2)
Crystal system	triclinic	triclinic
Space group	$P\overline{1}$	$P\overline{1}$
a/Å	13.4983(9)	12.1828(2)
b/Å	14.2512(9)	14.7546(2)
c/Å	15.1299(9)	18.8200(3)
α/°	90.871(3)	111.9860(10)
β/°	91.049(3)	96.1870(10)
γ/°	106.242(3)	96.0540(10)
Volume/Å^3	2793.2(3)	3079.38(8)
Z	2	2
ρ_{calc}/g cm^{-3}	1.398	1.397
μ/mm^{-1}	0.519	0.491
F(000)	1216.0	1324.0
Crystal size/mm^3	0.19 × 0.12 × 0.09	0.12 × 0.10 × 0.09
2Θ range for data collection/°	3.674 to 63.476	4.446 to 54.970
Index ranges	$-18 \leq h \leq 19$, $-20 \leq k \leq 20$, $-20 \leq l \leq 21$	$-15 \leq h \leq 15$, $-19 \leq k \leq 19$, $-24 \leq l \leq 24$
Reflections collected	34722	37616
Independent reflections	16277 [R_{int} = 0.0279, R_{sigma} = 0.0555]	13984 [R_{int} = 0.0321, R_{sigma} = 0.0451]
Data/restraints/parameters	16277/2/740	13984/36/788
Goodness-of-fit on F^2	1.027	1.025
Final R values [I>=2σ (I)]	R_1 = 0.0440, wR_2 = 0.0931	R_1 = 0.0591, wR_2 = 0.1378
Final R values [all data]	R_1 = 0.0729, wR_2 = 0.1052	R_1 = 0.0730, wR_2 = 0.1474
Largest diff. peak/hole/e Å^{-3}	1.04/-0.59	1.57/-0.95

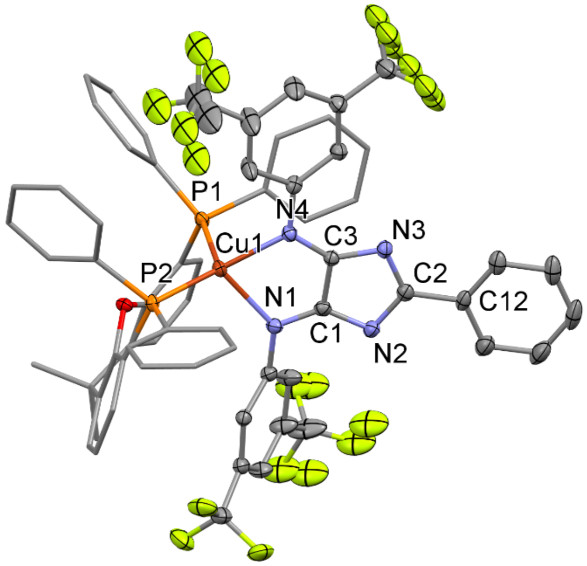

Figure S25. Molecular structure of **1** determined by single crystal X-ray diffraction. Ellipsoids are either drawn at the 50% probability level or were omitted for the xanthphos ligand. H atoms and solvent molecules have been omitted for clarity.

Table S5. Comparison of structural characteristics of **1** and **2**. The crystal structure of **2** has been published before.[3] The dihedral angle (DHA) is defined as the angle between the P–Cu–P and N–Cu–N planes.

	**1**	**2**
Cu–N / Å	2.078(2), 2.135(2)	2.098(2), 2.112(2)
Cu–P / Å	2.2178(7), 2.2837(7)	2.2417(5), 2.2868 (5)
N–Cu–N / °	82.40(9)	82.23(6)
P–Cu–P / °	119.64(3)	116.63(2)
DHA / °	88.49(7)	83.40(3)

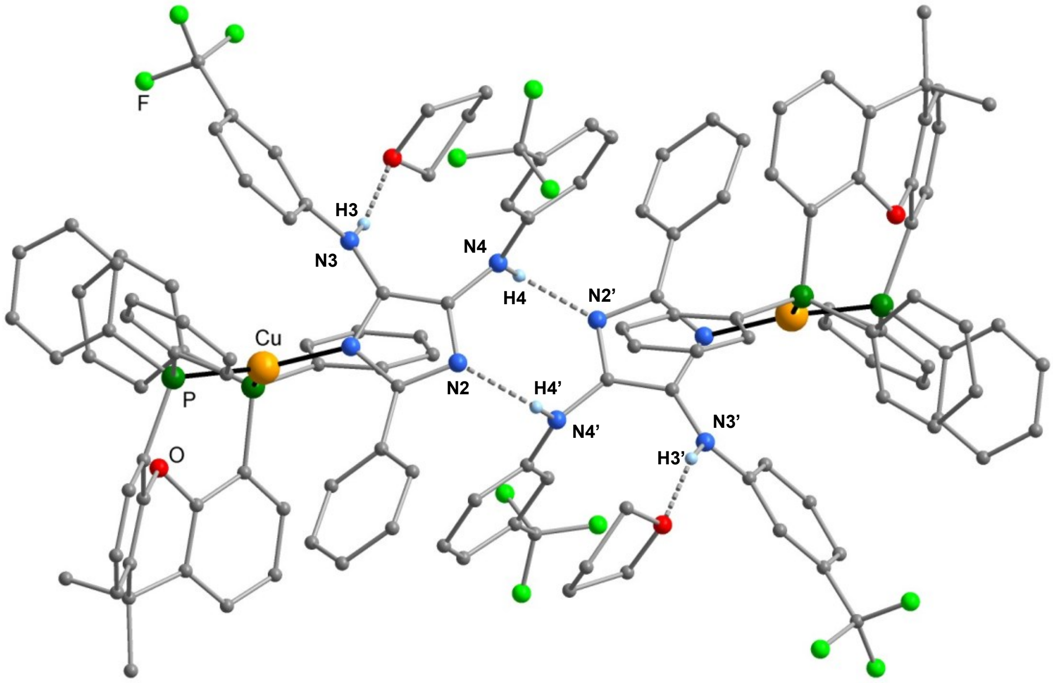

Figure S26. Molecular structure of $^{exo}H_2$**2** determined by single crystal X-ray diffraction showing the formation of hydrogen-bonded dimers. Hydrogen atoms attached to C- atoms are omitted for clarity.

8 NMR spectroscopic investigations

8.1 NMR spectroscopic following of the photoreduction of 2 with BIH

2 (3.94 mg, 0.0036 mmol) and BIH (1.77 mg, 0.0076 mg, 2.2 eq.) where dissolved in THF-d8 under nitrogen atmosphere. The solution in the NMR tube was irradiated *ex situ* with a 455 nm LED. ^{1}H-NMR spectra were collected after the indicated irradiation times. After irradiation the NMR tube was opened to check the reversibility of the photoreduction. A smaller amount of BIH was used in the NMR experiment than in the UV-Vis irradiation experiments to enable the observation of aromatic signals without pronounced BIH signal overlap.

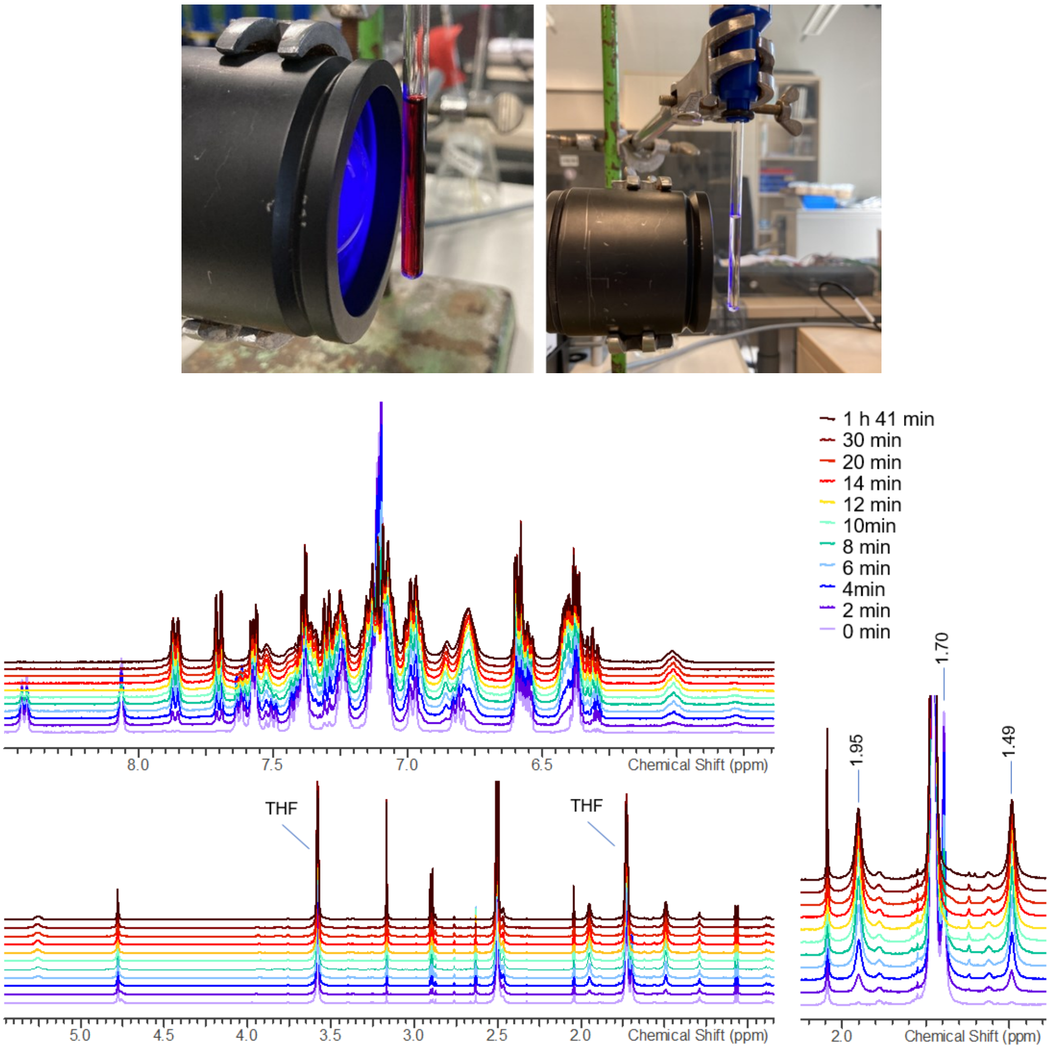

Figure S27. Photo of the experimental setup for NMR irradiation experiment. At the beginning of the irradiation the sample appears deep red (upper left), after an irradiation time of 1 h 41 min the sample appears colorless (upper right). ^{1}H-NMR spectra (THF-d8, 400 MHz, 297 K) of the reaction solution of **2** and BIH, were taken during photoreaction after certain irradiation times with 455 nm LED light as indicated in the legend (middle). The NMR spectra a dissected into the aromatic region (middle) and aliphatic region (lower left), and a magnification of the development of two xantphos methyl group resonances (1.95 and 1.49 ppm; lower right). With increasing irradiation times, the signals for **2** decreases while signals for the reaction products increase without the formation of observable intermediates. The radical intermediate cannot be observed in NMR spectroscopy; however, its presence was visible from the green color of the reaction solution after initial irradiation (not depicted).

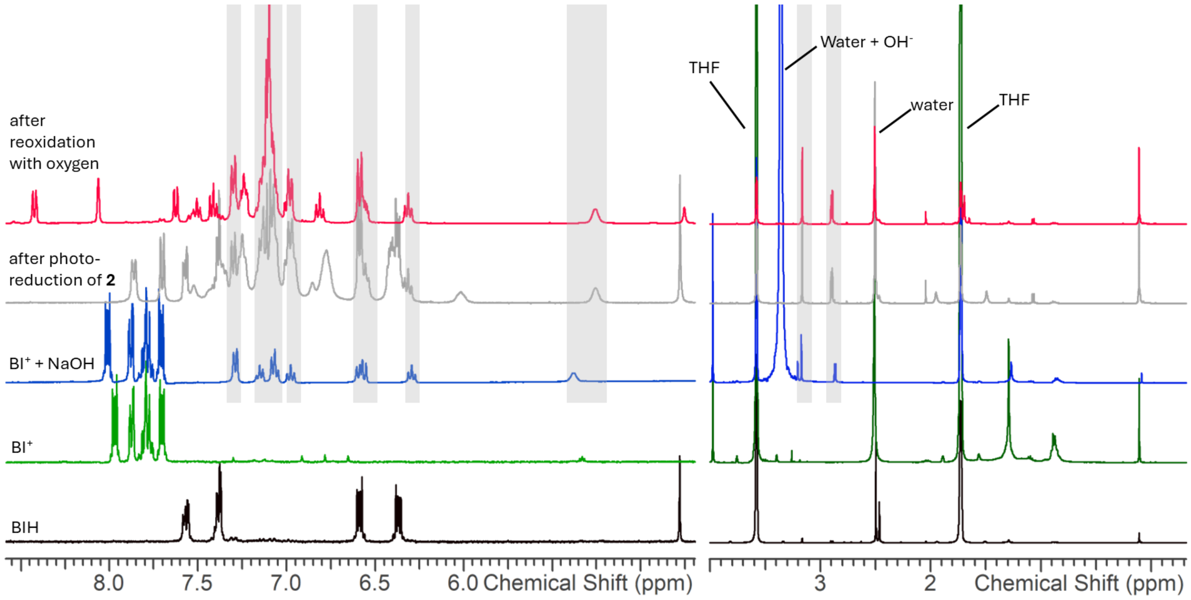

Figure S28. Comparison of the ^{1}H NMR spectra of BIH (300 MHz, THF-d8, 297 K), BI^+ (400 MHz, THF-d8, 297 K), BI^+ + NaOH (400 MHz, THF-d8, 297 K), the reaction solution of **2** after photoreduction with BIH (400 MHz, THF-d8, 297 K) and the reaction solution after reoxidation (400 MHz, THF-d8, 297 K). After photoreduction the signals for **2** disappeared and the spectrum consists of signals for the reduction product $^{exo}H_2$**2** and the BIH oxidation product. After reoxidation of the reaction solution with oxygen, signals for $^{exo}H_2$**2** disappeared and signals for **2** can be found in the spectrum. Signals for the BIH oxidation product increased after reoxidation and are marked in grey. The number and position of the BIH oxidation product do not fit to the formation of BI^+. Presumably BI^+ is involved in a follow-up reaction leading to the final oxidation product. BI^+ reacts in presence of OH^- to *N*-methyl-*N*-(2-(methylamino)phenyl)benzamide (BIox) (see Figure S29), as confirmed by comparison of the ^{1}H-NMR signals.[21] The presence of OH^- in the reaction solution can be explained by residual water, which acts as proton source in the photoreduction.

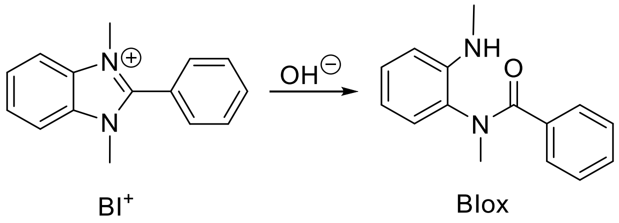

Figure S29. Reaction scheme for formation of BIox from BI^+.

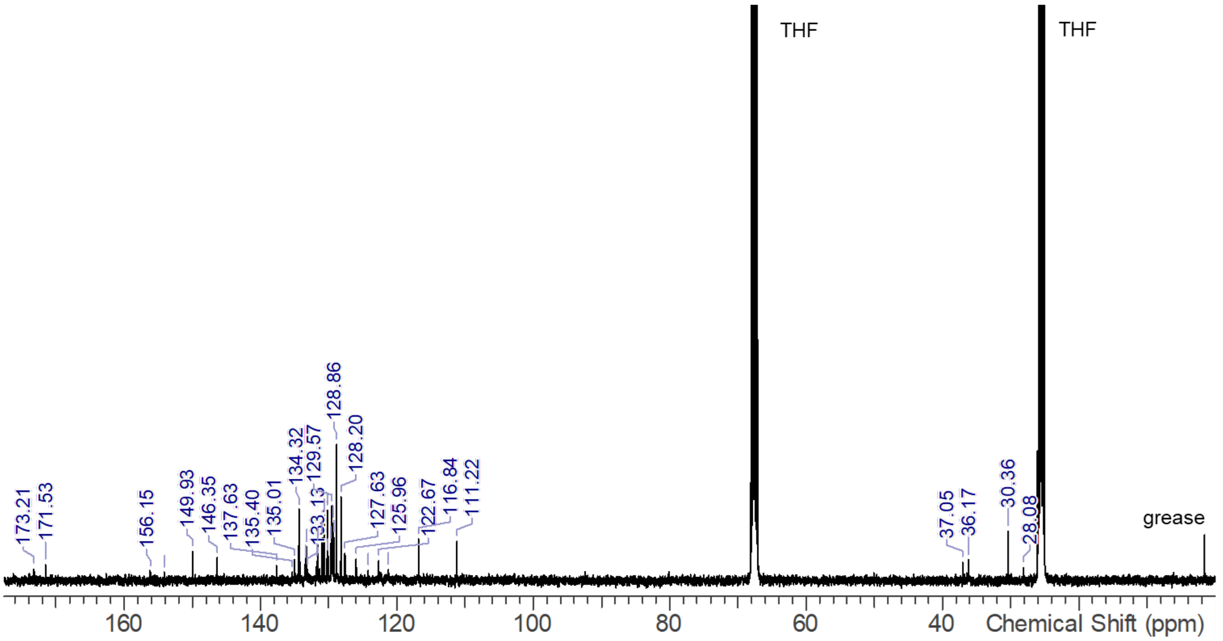

Figure S30. ^{13}C-NMR spectrum (THF-d8, 100 MHz, 297 K) of the reaction solution of **2** and BIH after photoreduction and reoxidation. The spectrum shows signals of **2**, BIH and the BIH oxidation product BIox.

8.2 NMR spectroscopic characterization of the isolated photo-reduction product $^{exo}H_2$**2**

2 (9.72 mg, 0,0088 mmol) was dissolved in 1.2 mL THF. In a separate vial BIH (40 mg, 0.18 mmol, 20.5 eq.) was dissolved in 2 mL THF and transferred to the **2** solution. The vial was positioned directly at the glass window of the glovebox and irradiated with white LED light (LED 2). When the color was completely changed from deep red to pale yellow the solution was layered with cyclohexane. After a few weeks pale yellow crystals formed.

The reaction solution was decanted with a syringe. The crystals were washed three times with ca. 100 µL cyclohexane and dried in vacuo. The crystals were transferred to a vial and dissolved in THF-d8 and then transferred to a young tube prior to NMR spectroscopy measurement.

1D-NMR spectroscopy

For assignment of the signals see main text Figure 4. Please note that the signals are divided into spin systems and labelled based on the 2D NMR spectra. However, it is not possible to precisely assign the signal sets to the spatial arrangement of the relevant aryl rings.

^{1}H-NMR (400 MHz, THF-d8, 297 K) δ/ppm = 1.49 (s, 3H, X8 or X8‘) 1.95 (s, 3H, X8 or X8‘) 6.00 - 6.07 (m, 1H, C5H$^{N\text{-}Ar‘}$) 6.31 (br s, 1H, NH$^{N\text{-}Ar‘}$) 6.35 - 6.46 (m, 5H, C2H^{X}, C3H$^{2\text{-}Ph}$, C6H$^{N\text{-}Ar‘}$) 6.55 (m, 2H, C4H$^{2\text{-}Ph}$, C4H$^{N\text{-}Ar‘}$) 6.71 - 6.81 (m, 5H, C4H$^{N\text{-}Ar}$, C2H$^{XPh‘}$) 6.83 (br s, 1H, NH$^{N\text{-}Ar}$) 6.91 - 7.03 (m, 5H, C2H$^{N\text{-}Ar‘}$, C3H$^{XPh‘}$) 7.03 - 7.18 (m, 9H, C5H$^{N\text{-}Ar}$, C3H^{X}, C2H$^{X\text{-}Ph}$, C4H$^{X\text{-}Ph‘}$) 7.25 (br t, *J*=7.31 Hz, 4H, C3H$^{X\text{-}Ph}$) 7.32 - 7.38 (m, 2H, C4H$^{X\text{-}Ph}$) 7.44 (br d, *J*=7.89 Hz, 1H,

C6H^{N-Ar}) 7.54 (br s, 1H, C2H^{N-Ar}) 7.70 (d, *J*=7.02 Hz, 2H, C4H^{X}) 7.87 (d, *J*=7.31 Hz, 2H, C2H^{2-Ph}).

^{13}**C-NMR** (101 MHz, THF-d8, 297 K) δ/ppm = 25.2 (C8^{X} or C8$^{X'}$) 32.23 (C8^{X} or C8$^{X'}$) 36.86 (C7^{X}) 111.14 (C2$^{N-Ar'}$) 111.28 (C2^{N-Ar}) 112.78 (C4^{N-Ar}) 113.45 (C4$^{N-Ar'}$) 116.78 (C6$^{N-Ar'}$) 117.97 (C6^{N-Ar}) 121.23 (C1^{X}) 123.97 (not assigned) 125.03 (C4^{2-Ph}) 125.33 (C2^{2-Ph}) 126.02 (C3^{X}) 128.11 (C4^{X}) 128.31 (C3^{2-Ph}) 129.20 (C1$^{X-Ph'}$) 129.36 (C3$^{XPh'}$) 129.77 (C3^{XPh}) 130.38 (C4$^{XPh'}$) 130.61 (C5$^{N-Ar'}$) 131.15 (C4^{XPh}) 132.59 (C2^{X}) 132.84 (C1^{XPh}) 133.54 (C2$^{XPh'}$) 134.68 (C5^{X}) 135.34 (C2^{XPh}) 138.05 (C1^{2-Ph}) 145.83 (C2^{Im}) 149.10 (C1^{N-Ar}) 150.83 (C1$^{N-Ar'}$) 155.81 (C6^{X})

^{19}**F-NMR** (376 MHz, THF-*d8,* 297 K) δ/ppm = –63.01, –63.30

^{31}**P-NMR** (162 MHz, THF-*d8,* 297 K) δ/ppm = –17.63

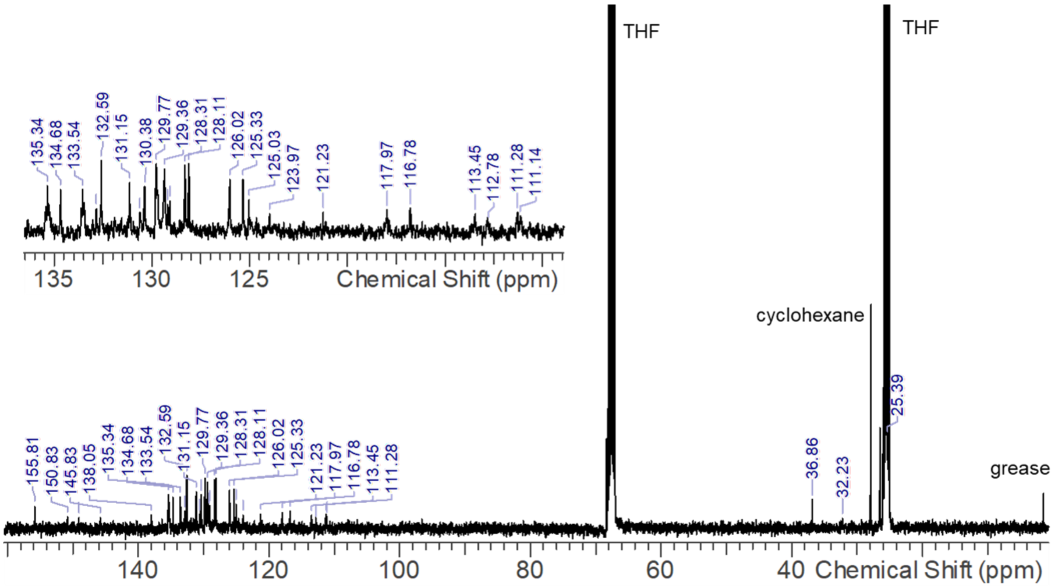

Figure S31. ^{13}C-NMR spectrum (THF-d8, 100 MHz, 297 K) of ^{exo}H$_2$**2**

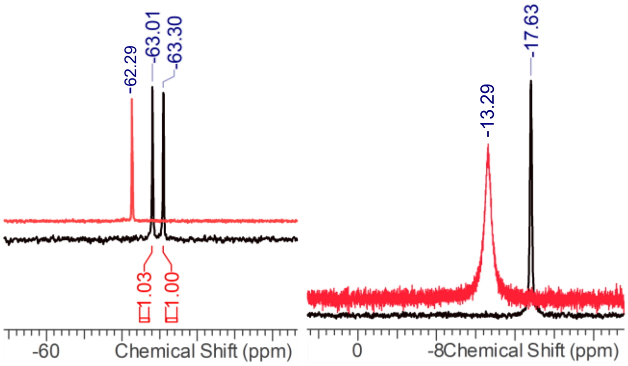

Figure S32. Comparison between ^{19}F-NMR spectra (THF-d8, 376 MHz, 297 K, left) and ^{31}P-NMR spectra (THF-d8, 162 MHz, 297 K, right) of **2** (red,) ^{exo}H$_2$**2** (black).

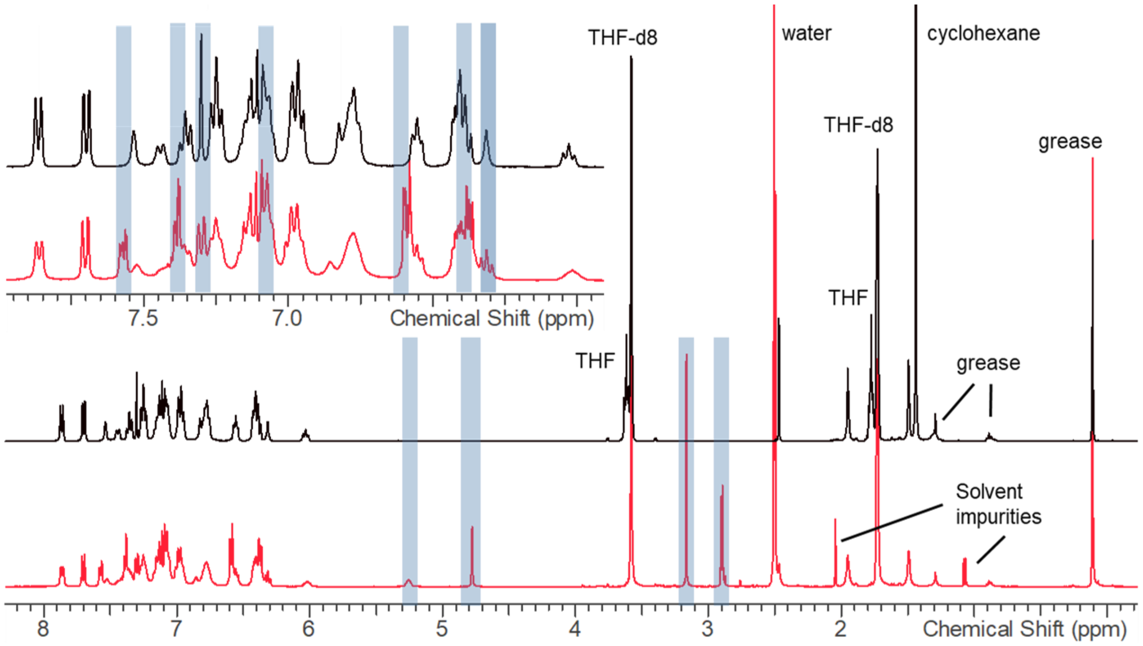

Figure S33. Comparison between the ^{1}H-NMR spectrum of $^{exo}H_2$**2** (black, THF-d8, 400 MHz, 297 K) and the reaction solution of **2** in presence of BIH after the photoreduction (red, THF-d8, 400 MHz, 297 K) with signals from BIH and the BIox marked in grey. Except for a broadening of the signals and minor shifts, the spectrum of the reaction solution after photoreduction resembles the spectrum of the isolated reduction product.

8.2.1 2D-NMR spectroscopy of $^{exo}H_2$**2**

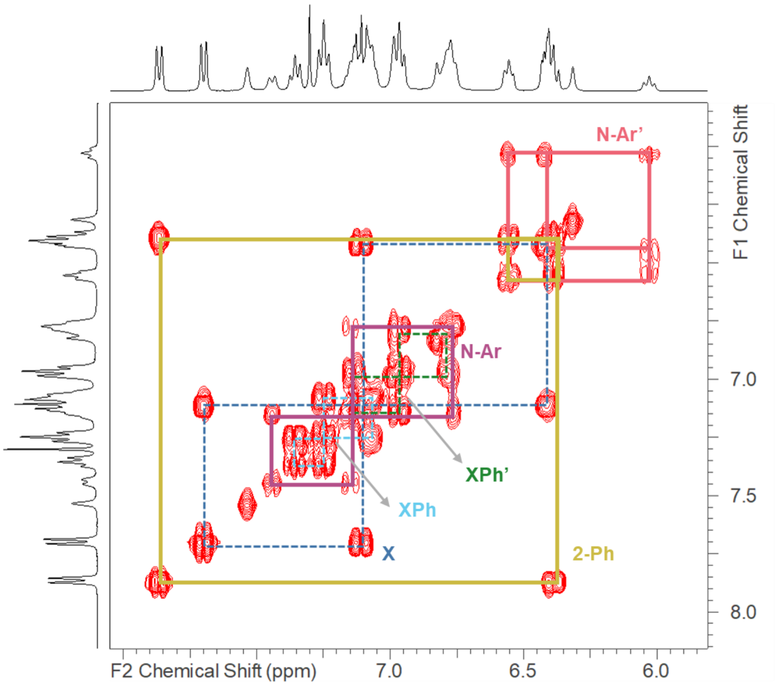

Figure S34. ^{1}H-^{1}H-COSY NMR spectrum (THF-d8, 400 MHz, 400 MHz, 297K) of $^{exo}H_2$**2** with assignment to the respective spin systems, for color code compare main text figure 4.

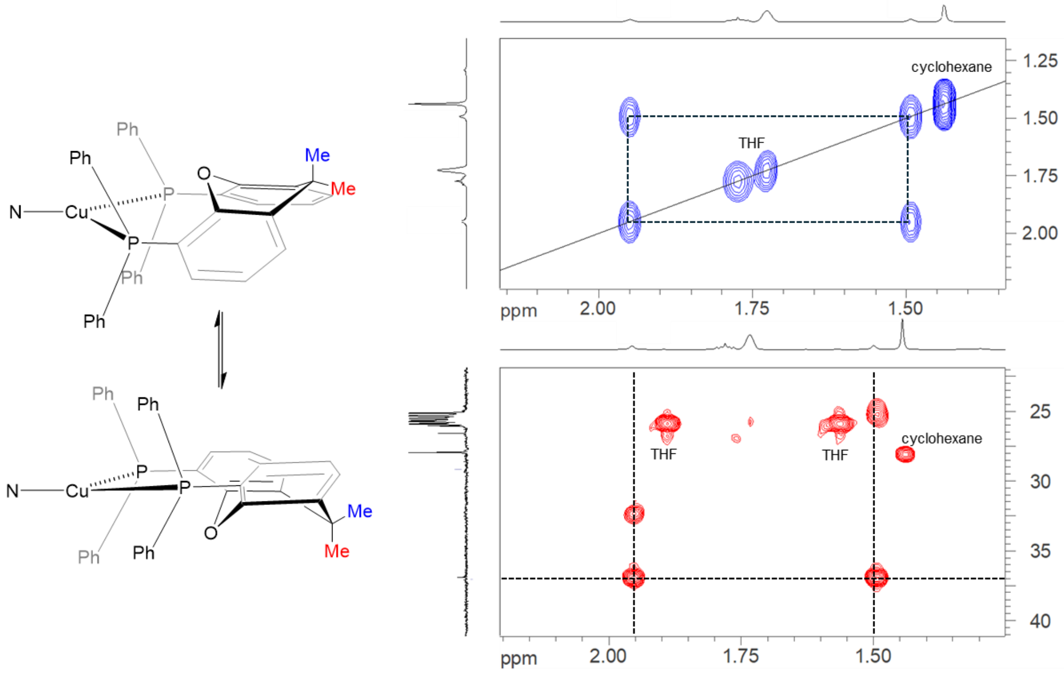

Figure S35: Schematic depiction of the xanthen boat inversion leading to chemical exchange between the axial and equatorial methyl groups. The corresponding cross peaks are shown in the depicted 2D-NMR spectra of $^{exo}H_2$**2**. Top: ^{1}H-^{1}H-EXSY-NMR spectrum (THF-d8, 400 MHz, 400 MHz, 297 K). Bottom: ^{1}H-^{13}C-HMBC-NMR spectrum (THF-d8, 400 MHz, 100 MHz, 297 K).

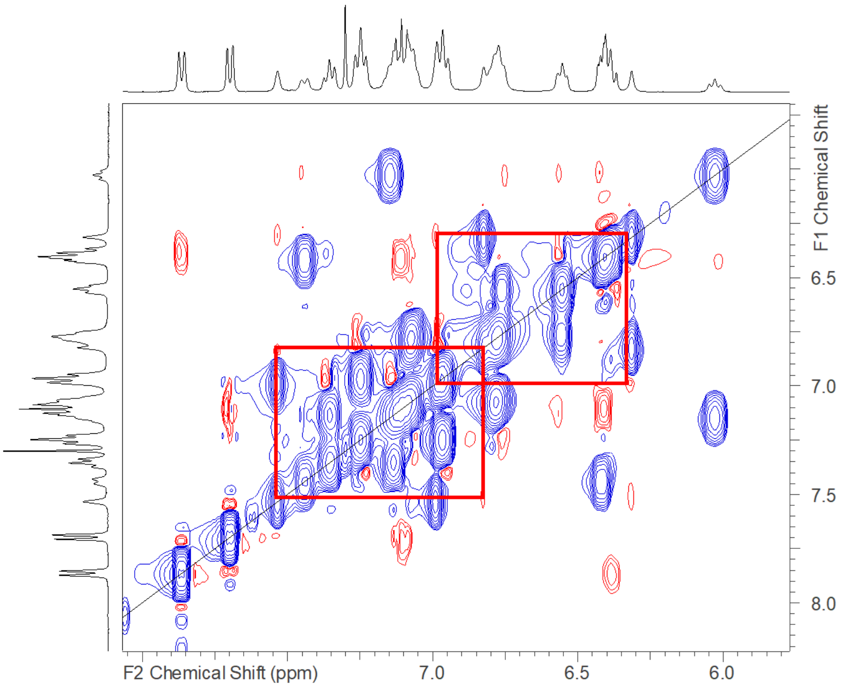

Figure S36: Aromatic region of the ^{1}H-^{1}H-EXSY-NMR spectrum (THF-d8,400 MHz, 400 MHz, 297 K) of $^{exo}H_2$**2**. Weak NOESY peaks are shown in red. NOE cross signals between N-H protons and N-Ar2/N-Ar'2 are marked in red.

8.2.2 Variable temperature NMR-spectroscopy of $^{exo}H_2$2

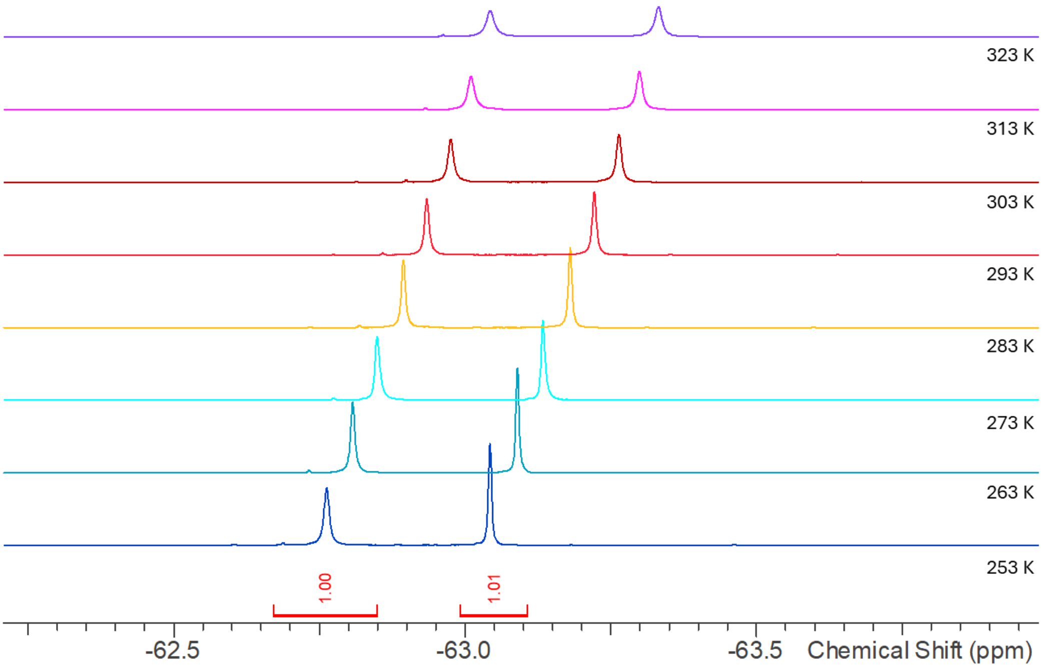

Figure S37. Temperature-dependent ^{19}F-NMR-spectra (THF-d8, 500 MHz) of $^{exo}H_2$**2**. Integral values are shown for the spectrum at 253 K.

8.2.3 ^{1}H-DOSY NMR spectroscopy of ^{exo}H$_2$**2**

The ^{1}H-DOSY-NMR measurements resulted in a diffusion coefficient of $5.88 \cdot 10^{-10}$ m^2 s^{-1}. The relationship between the molar mass and the diffusion coefficient was used to distinguish between a dimer and a monomer in solution. The molar mass of the reduced complex in solution was estimated using the external calibration method with normalized diffusion coefficients.[22] Here the molar mass was predicted using different models for the shape of the molecule in its solvent environment. Regardless of the assumed shape of the diffusing unit, the results are more consistent with the molar mass of a monomeric species of the reduced complex (Table S6).

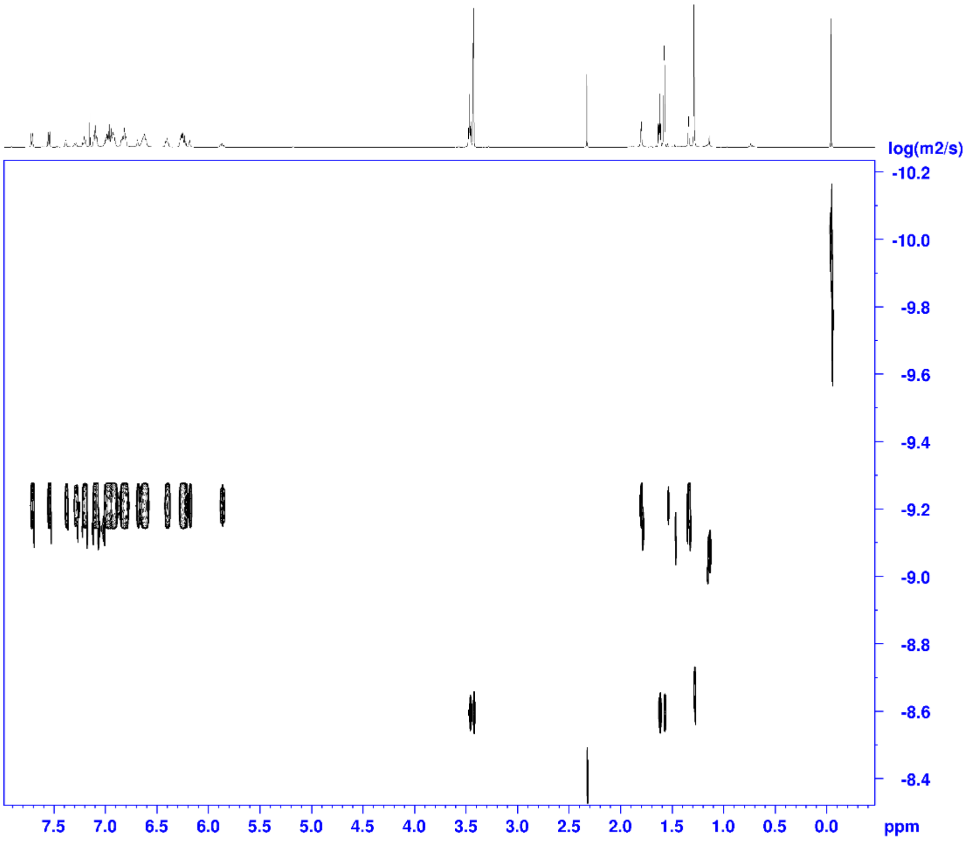

Figure S38. ^{1}H-DOSY NMR spectrum (THF-d8) of ^{exo}H$_2$**2**

Table S6. The predicted molar weights using the external calibration method[22] for different shapes of the diffusion molecule. The molar weight of the monomeric species is 1103.57 g·mol^{-1}.

**Model**	**Molar weight prediction [g·mol^{-1}]**
Highly compact spheres (in THF)	1112
Dissipated Spheres + Ellipsoids (in THF)	872
Expanded discs	676
Merged (in THF)	839

9 Spectral characteristics of LEDs used in the irradiation experiments

White light LED powers were determined with a thermopile sensor LM-10 HTD (Coherent). Powers for LEDs which were used in the irradiation experiments were determined while the LEDs were equally connected to the irradiation setup as in the respective experiments. The power detected in this measurement arrangement equals the total power hitting the cuvette during irradiation experiments. The output spectrum of the white light LED 1 was determined with a fiber-coupled diode array spectrometer (Avantes AvaSpec) and corrected for the dark current. Output spectra for other LEDs were taken from the respective Thorlabs data sheets.

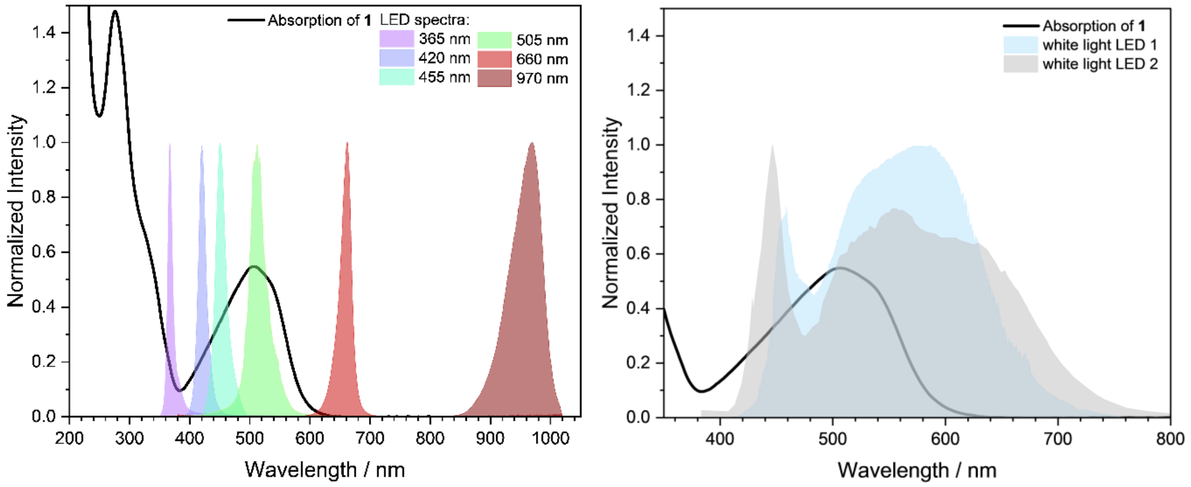

Figure S39. Normalized intensities of the mounted LEDs with nominal wavelengths at 365 nm, 455 nm, 505 nm, 660 nm and 970 nm (left). Normalized intensities of the white LED 1 and white light LED 2 (right). For comparison LED-Spectra are shown in front of the scaled absorption spectrum of **1.**

10 Fs-transient absorption spectroscopy of 2 and radical intermediate

In order to investigate the reduction process of **2*** to $\mathbf{2}^{\bullet-}$ and to determine the lifetime of the latter, transient absorption (TA) spectra of **2** were recorded with and without the addition of the electron donor BIH. In order to minimize the accumulation of $\mathbf{2}^{\bullet-}$ in the measurement spot the cuvette was moved in the x-y plane. Taking into consideration the diameter of the laser beam, (ca. 600 µm) and the relatively slow (70 µm/s) movement of the cuvette, it can be assumed that both **2** and $\mathbf{2}^{\bullet-}$ were present in solution upon excitation. The ratio of both species is thus dependent on the number of BIH equivalents used in the respective measurement. Even though this makes an accurate determination of the electron transfer rate impossible, the excited state lifetime of $\mathbf{2}^{\bullet-}$ can be extracted from the obtained data as we will show in the following. Figure S40 a-c shows the TA spectra obtained with zero, two and thirty equivalents of BIH.

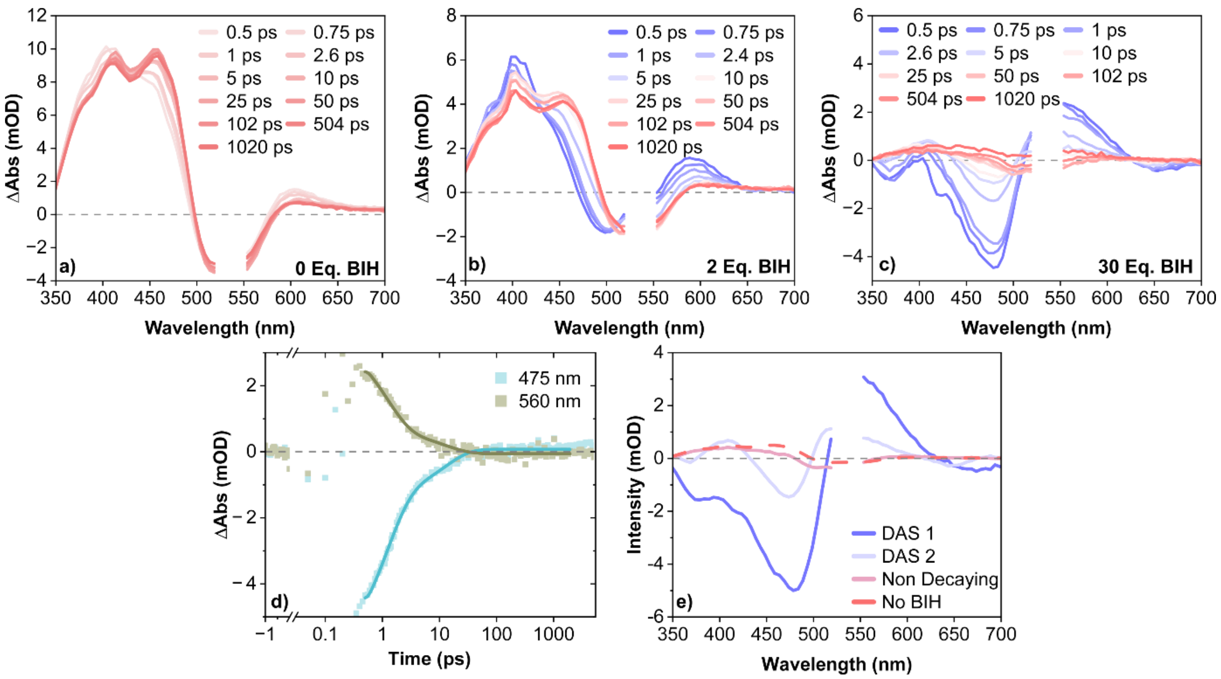

Figure S40. Transient absorption spectra of **2** in acetonitrile under inert conditions and at room temperature. While a) reflects the photophysics of the isolated complex, the spectra in b) and c) were obtained after the addition of 2 and 30 eq. of BIH, respectively. The transient kinetics of the latter are shown in d) for the wavelengths 475 and 560 nm. Global analysis of this data set with a combination of two exponential decay components and one non decaying component yielded the decay-associated spectra (DAS) presented in e) on which the (scaled) long-lived DAS of a) is superimposed. During this evaluation the temporal region between -1 and 0.45 ps was excluded from the fit due to strong contributions from the coherent artifact. In all cases excitation was performed at 532 nm and the wavelength region 525-555 nm was omitted due to strong pump scattering. In all cases 1 mm cuvettes were used. a), b) and c) are also presented in the main text, Figure 8.

If no electron donor is present in the solution, the transient spectra show comparable evolution to that previously observed for structurally similar Cu(I) complexes.[11] At all times the spectra are dominated by a broad and intense excited-state absorption (ESA) extending from 350 to 495 nm and another, less intense one extending from 585 to 700 nm. The wavelength region in between shows ground-state bleach that coincides with the ground state absorption spectrum (see Figure S1). Upon closer inspection, the former ESA band consists of two separate peaks at 404 and at 459 nm, which decrease and increase in intensity, respectively, as the pump-probe delay increases. This was previously associated with the planarization process of the complex upon excitation.[3,11] Further, more subtle changes in the form of spectral shifts are observed both at very short and at longer delay times. These were previously attributed to flattening and internal conversion processes, respectively. At very long (> 1 ns) delay times, part of the ground state recovery process is seen. By applying a parallel model with four separate components we stick to our previous analysis of the TA data recorded for similar complexes and obtain the fit constants displayed in Table S7. These are, overall, in good agreement with our previous results to which the reader is referred to for a more detailed discussion of the involved processes.[11,23]

When BIH is added to the solution prior to excitation, the resulting TA spectra change significantly: When two equivalents of the electron donor are added, good agreement with the previously discussed spectra is observed for long delay times (approximately >50 ps). However, at shorter pump-probe delays, two additional ESA bands can be observed: one at

585 nm and one that partially overlaps with the ESA features of the non-reduced complex at 400 nm. Additionally, a GSB feature is observed at 500 nm for short delay times. Increasing the concentration of BIH to 30 equivalents makes these spectral signatures even more prominent, with the relevant ESA features shifting to 550 and 405 nm, and the GSB signature appearing at 480 nm. It is also striking that the majority of spectral signatures have now decayed on a timescale of tens of ps (see Figure S40 d). Nonetheless, this measurement also contains some slowly decaying components (>1 ns). Upon closer inspection, it becomes clear that the long-lived signal contributions are in good agreement with the signatures observed when no BIH is added to the solution.

Based on these results, we associate the long-lived signatures in the measurement with 30 eq. BIH to **2** that has been optically excited by the pump pulse but did not get reduced by BIH after excitation. However, due to the high concentration of electron donor, the fraction of **2*** is small. This is also reflected in the relative intensity of these slowly decaying TA signatures. We account for this residual population of non-reduced complex by adding a non-decaying component to our model (*vide infra*). To validate this approach, we compare the decay associated spectrum (DAS) obtained for this component with the (scaled) long-lived DAS obtained from the data set of **2** without BIH in Figure S40 a) and find good agreement between the two of them (see Figure S40 e). We assume, that the majority of **2*** reacts with BIH to form $\mathbf{2}^{\bullet-}$. Considering the absorption spectrum of $\mathbf{2}^{\bullet-}$, its optically excitation by the pump light (532 nm) cannot be prevented. Comparison of the GSB features with the absorption spectrum of $\mathbf{2}^{\bullet-}$ further supports the assignment of these features to $\mathbf{2}^{\bullet-}$*. Under the assumption that, within our model, the TA signatures at early times are mostly dominated by contributions from $\mathbf{2}^{\bullet-}$*, it is possible to isolate the photophysics of this species. For an accurate fit of these kinetics, two monoexponentially decaying components are further needed in our model (Figure S40) and the associated time constants obtained by the global fitting of the data amount to 1 ps and 13 ps, respectively (see Table S7).

Considering the absorption spectrum of the radical (see Figure S4 and Figure S21), it is likely that excitation at 532 nm is first followed by vibrational relaxation processes that correspond to the first time constant. Under this assumption, the lifetime of $\mathbf{2}^{\bullet-}$* is given by the second time constant, which is 13 ps. This is much smaller than the lifetime of **2***. The respective DAS obtained from the global fit[24] also point in this direction as they show a pronounced blue shift between DAS 1 and DAS 2 (see Figure S40 e).

As mentioned above, this method unfortunately does not allow us to extract the involved electron transfer time between **2** and BIH as the formed radical $\mathbf{2}^{\bullet-}$ accumulates in solution over time. In a future study we plan on repeating these experiments in a flow-through cell in order to understand whether the electron transfer step is the result of a preassociated **2**/BIH dimer or if it occurs at the diffusion limit of the two reactants.

Table S7. Fitted time constants for **2** in acetonitrile under inert conditions without and with the addition of BIH. In the former case the standard deviations are obtained from averaging four separate measurements.

	$\tau_{Flattening}$ (ps)	$\tau_{Planarization}$ (ps)	τ_{IC} (ps)	$\tau_{GS\ Recovery}$ (ns)
**2***	0.8±0.02	8±2	102±13	44±19
	$\tau_{Vibr.\ Relax.}$ (ps)	$\tau_{GS\ Recovery}$ (ps)	Non decaying	
**2**$^{\bullet-}$*	1	13	yes	

11 DFT calculations

11.1 Computational details

The quantum chemical calculations were performed using the Gaussian 16 program[25]. Firstly, the equilibrium geometries of four conformers of **2** were obtained within its singlet ground state by means of density functional theory (DFT) using the correlation-exchange functional B3LYP.[26,27] The def2SVP basis set was used for all elements.[28] A vibrational analysis was performed to verify that the obtained geometries represent minima on the potential energy surface. On the basis of the most stable conformer, the geometries at various redox and protonation states were optimized at the same level of theory, whereas frequency calculations were performed to confirm the nature of the local minimum geometries.
Secondly, the excited-state properties, namely vertical transition energies (ΔE), oscillator strengths (*f*), and the character of electronic transitions, were calculated at time-dependent density functional theory (TD-DFT) level of theory. Therefore, the same basis set and functional as for the ground state DFT simulations was used. In general, the lowest 150 singlet-singlet or triplet-triplet transitions were calculated within the respective multiplicity of the electronic ground state (that is singlet-singlet or doublet-doublet transitions). The solvent effects for acetonitrile (ε=35.69, n=1.344) were considered in the predictions of the properties of the ground state using the electron density variant of the integral equation formalism of the polarizable continuum model.[29,30] The non-equilibrium solvation model was employed in the computation of the excited-state properties. All calculations were performed including Grimme D3 dispersion correction with Becke-Johnson damping.[31]

11.2 Neutral complex

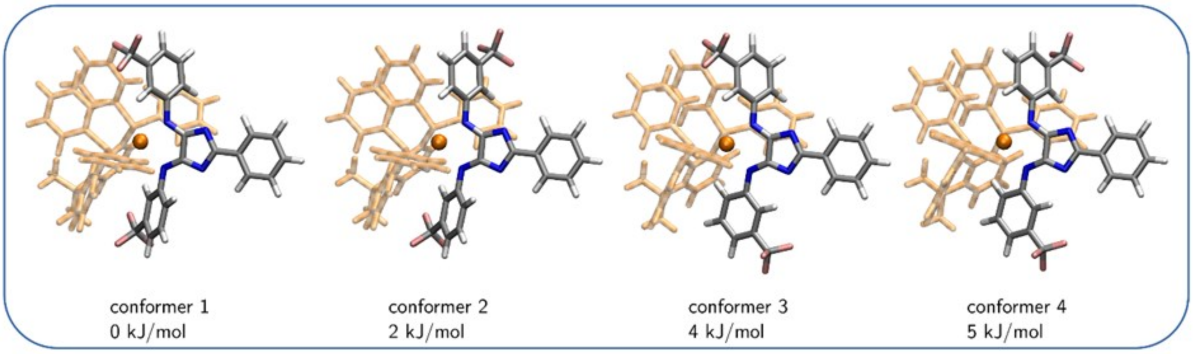

Figure S41. Overview of the local minimum structures of four conformers of the neutral complex (**2**) and their relative energies with respect to the minimum energy conformer (conformer 1).

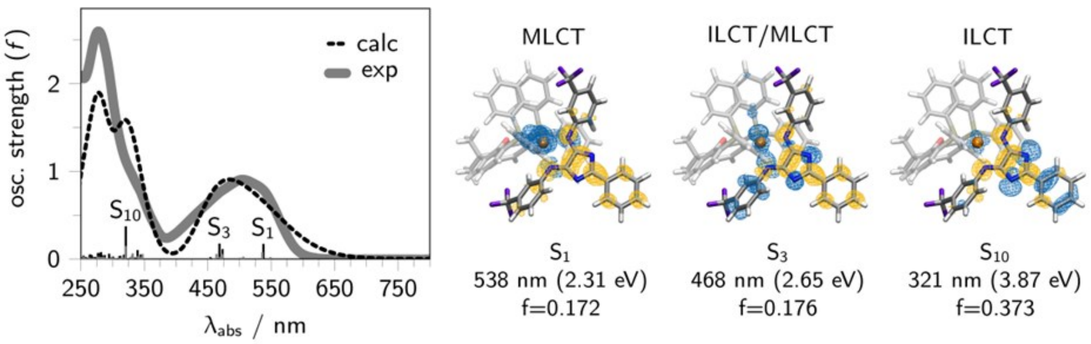

Figure S42. Simulated absorption spectrum of the neutral complex **2** in acetonitrile, incorporating the Boltzmann-weighted contributions of the four conformers (see Figure S41). Selected charge density differences for the most stable conformer (conformer 1) are displayed, with excitation depicted as transitions from blue to yellow. The scaled experimental absorption spectrum measured in acetonitrile solution is overlaid as a thick line for comparison.

11.3 Singly-reduced complex

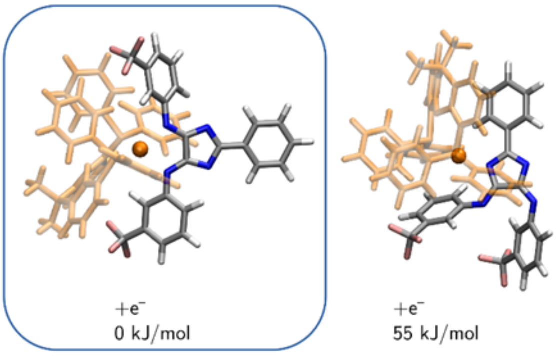

Figure S43. Local minimum geometries of the singly-reduced species of complex **2** in tetrahedral (blue box) and trigonal coordination environments. The tetrahedral geometry is favored by 55 kJ/mol relative to the trigonal form.

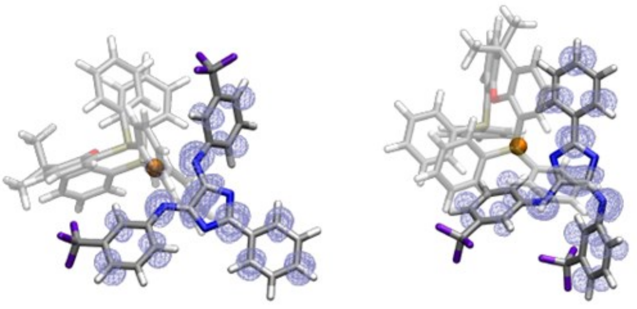

Figure S44. Spin density distributions in the singly-reduced complex of **2** in tetrahedral (left) and trigonal (right) coordination geometry.

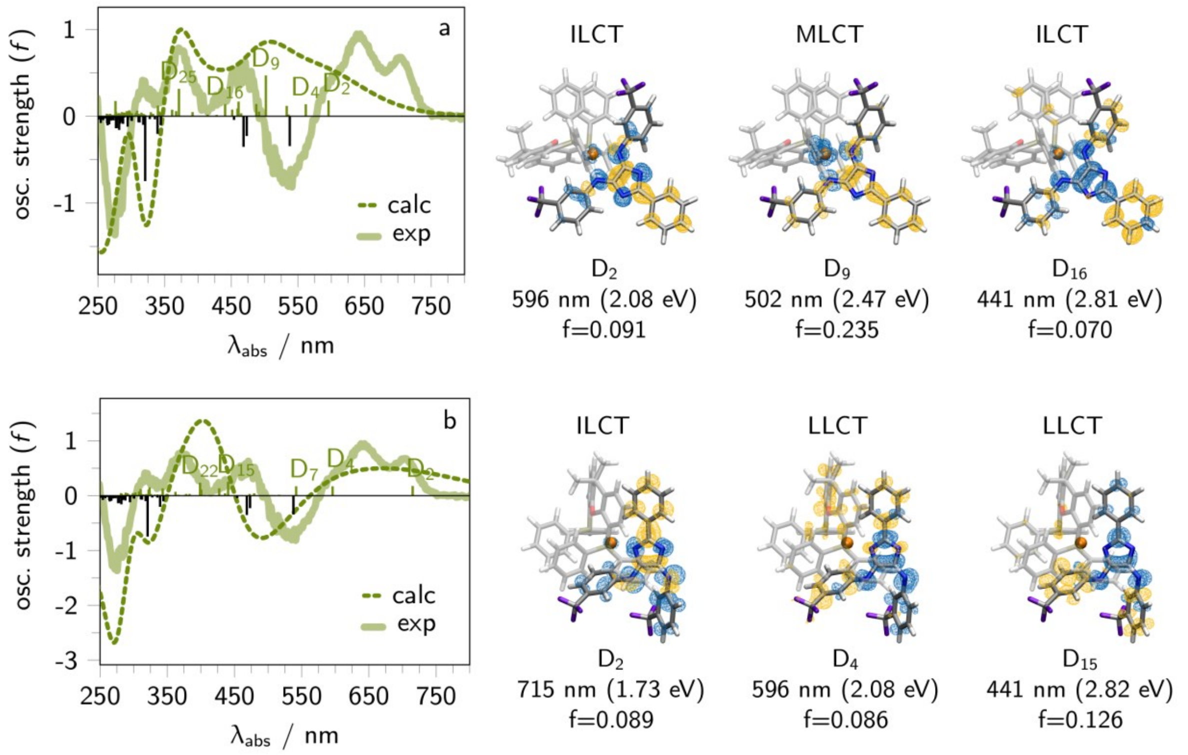

Figure S45. Simulated difference absorption spectra of the singly-reduced complex of **2** (positive signal region) in tetrahedral (a) and trigonal (b) coordination sphere with respect to the neutral complex (negative signal region, based on the most stable neutral conformer) in acetonitrile. For selected transitions the charge density differences are shown (as indicated by the state number), with excitation transitions illustrated from blue to yellow. The scaled experimental difference absorption spectrum, measured *via* spectroelectrochemistry in acetonitrile solution, is shown as a thick line for comparison.

The spin density of $\mathbf{2}^{\bullet-}$ is delocalised on the 4*H*-imidazolato ligand in agreement with the experimental findings and independent of the coordination mode. For the tetrahedral $\mathbf{2}^{\bullet-}$, the characteristic red absorption bands at about 600 and 750 nm are computationally less pronounced but can be assigned to the D_2 state. This state is ILCT in character, localizing electron density from the 4*H*-imidazolate core to the 2-aryl moiety. The transitions at 455 nm and 375 nm are attributed to ILCT (D_{16}, D_{25}) and MLCT (Cu→4*H*-imidazolate, D_9) processes. The negative peak at 550 nm in the experimental spectrum is not exactly reproduced by the computational data. Here, the calculated transitions D_4 and D_7 are of mixed MLCT/ILCT character. The MLCT state is comparable to the MLCT of **2** but blue-shifted and the intensity is likely overestimated.

11.4 Singly-reduced, singly-protonated complex

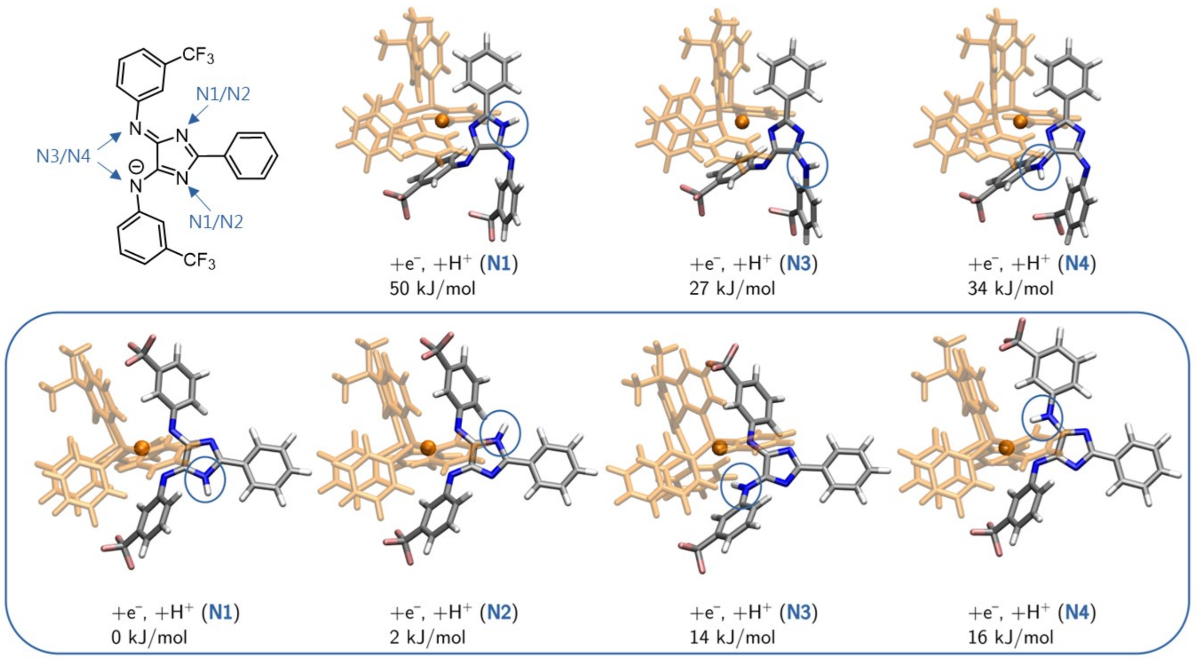

Figure S46. Visualization of the local minimum geometries of the singly-reduced and singly-protonated forms of copper complex **2**. Protonation was evaluated at each nitrogen atom of the 4*H*-imidazolato ligand (**N1**–**N4**, see top left). The top and bottom rows display the corresponding equilibrium structures in trigonal- and tetrahedral coordination geometries, respectively. Relative energies with respect to the global minimum (tetrahedral geometry, protonation at **N1**) are shown beneath each structure.

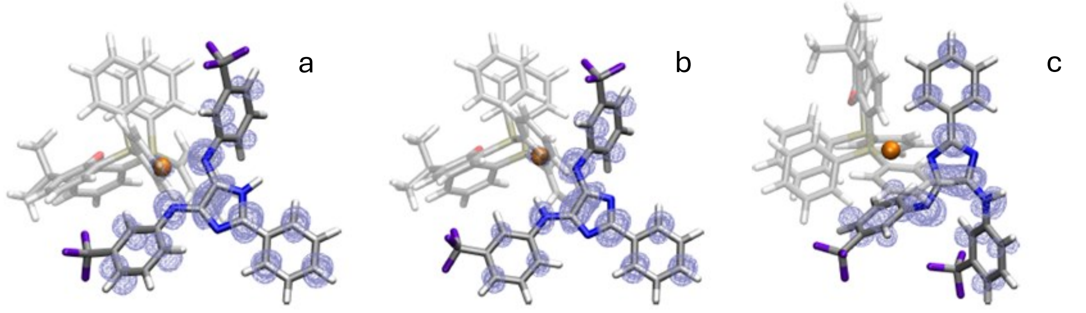

Figure S47. Spin density distributions of different isomers of the singly-reduced and singly-protonated complex of **2,** where protonation occurred at the central ring (a, protonation at **N1**) of the imidazolato ligand or at the exo-cyclic nitrogen atoms of the imidazolato ligand (b and c, protonation at **N3**) in tetrahedral (a and b) or trigonal (c) coordination geometry.

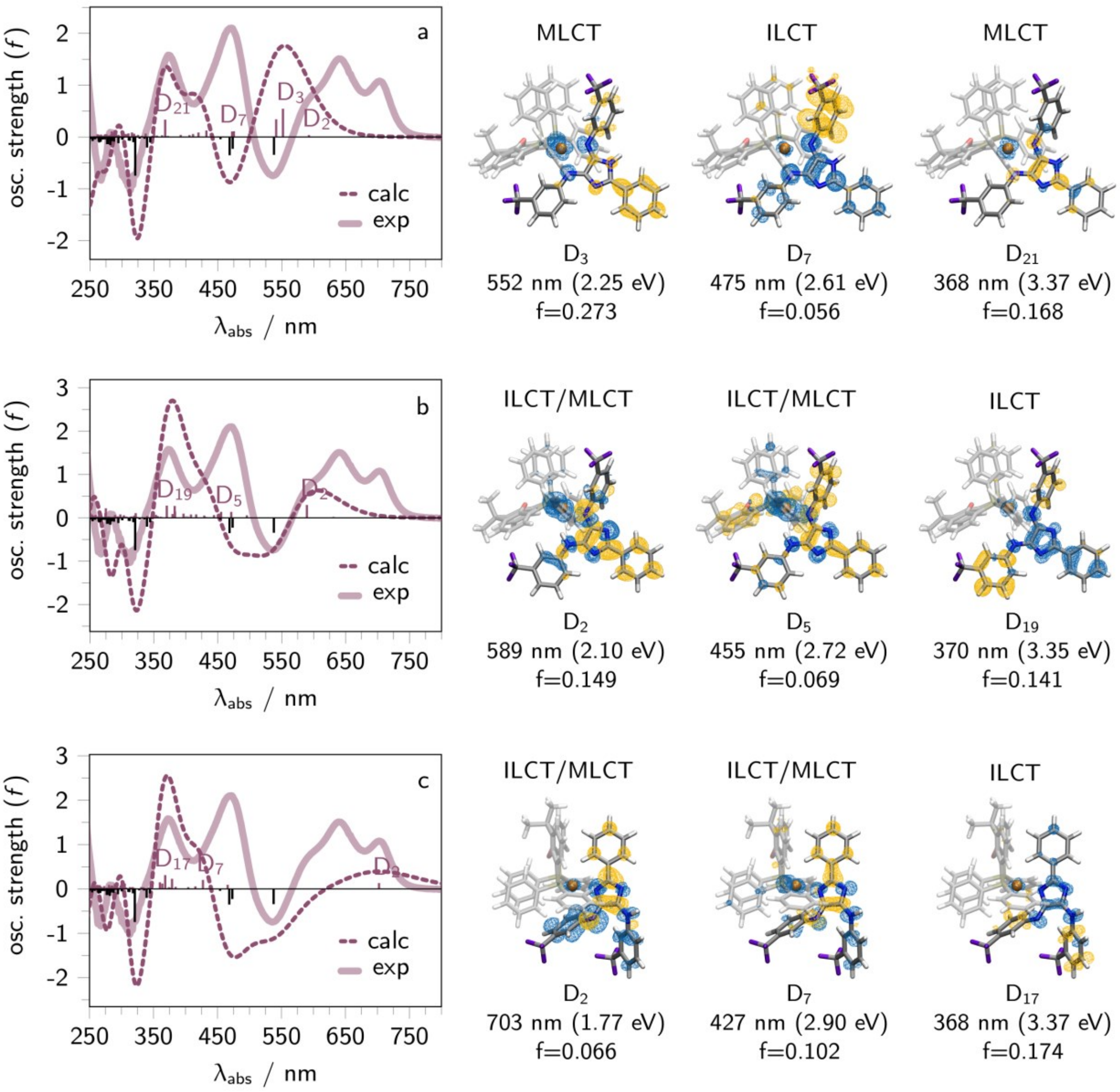

Figure S48. Simulated difference absorption spectra in acetonitrile comparing the singly-reduced and singly-protonated complexes of **2** (positive signal region) with tetrahedral coordination sphere and protonation at **N1** (a) and **N4** (b) or trigonal coordination sphere and protonation at **N3** (c) with the neutral complex (negative signal region, based on the most stable neutral conformer). Selected charge density differences for the singly-reduced and singly-protonated complexes are shown, with excitation transitions illustrated from blue to yellow. The scaled experimental difference absorption spectrum, taken from the reaction solution in acetonitrile after initial irradiation, is shown as a thick line for comparison.

The spin density of the singly-reduced singly-protonated H**2**$^{\bullet}$ is delocalized on the 4*H*-imidazolato ligand and independent of the coordination mode or protonation site (see Figure S46). The prominent experimental peak at 455 nm is not reproduced by the calculations but weak bands are predicted in this region in the tetrahedral coordination mode (*e.g.* D_5 and D_7; *cf.* Figure S48 a and b), while practically no optical transitions are predicted in this spectral region for the trigonal coordination (see Figure S48 c). The experimental peak at 355 nm is reproduced for both coordination modes and can be assigned to MLCT transitions from the copper center to the 4*H*-imidazolato ligand (Z=4, **N1/2**) or ILCT transitions from the 4*H*-imidazolate core to the *N*-aryl rings (Z=4, **N3/4** and Z=3, **N3/4**).

11.5 Doubly-reduced, singly-protonated complex

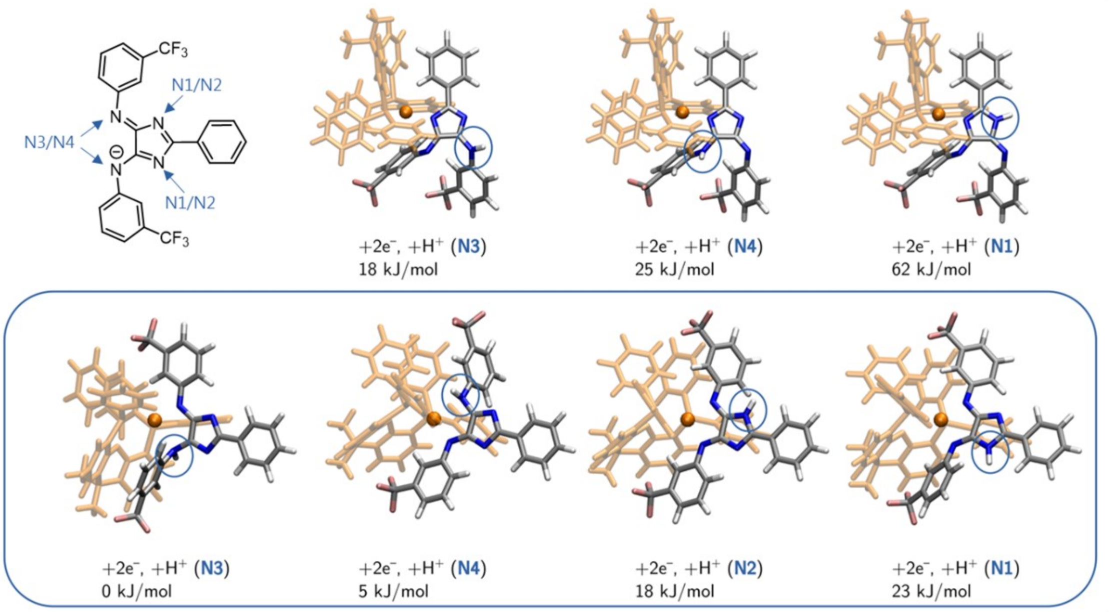

Figure S49. Visualization of the local minimum geometries of the doubly-reduced and singly-protonated forms of copper complex **2**. Protonation was evaluated at each nitrogen atom of the reduced 4*H*-imidazolato ligand (**N1–N4**, see top left). The top and bottom rows display the corresponding equilibrium structures in trigonal- and tetrahedral coordination geometries, respectively. Relative energies with respect to the global minimum (tetrahedral geometry, protonation at **N3**) are shown below each structure.

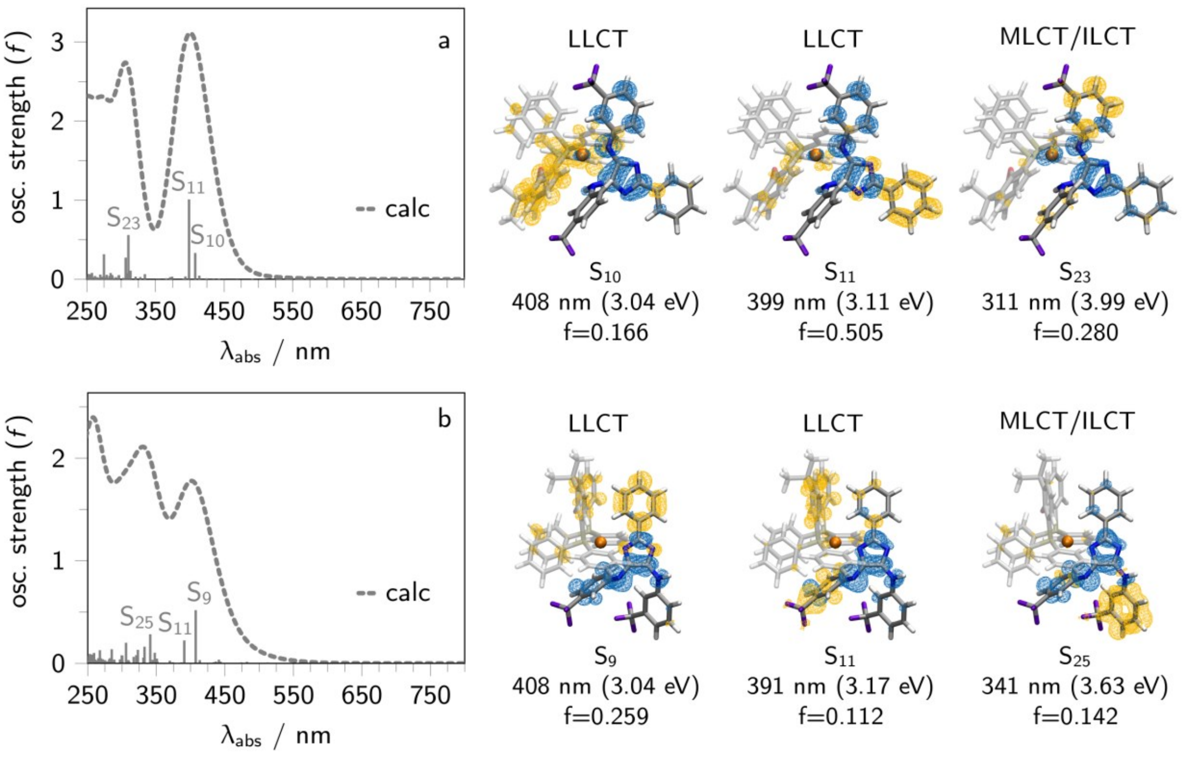

Figure S50. Simulated absorption spectra of the most stable conformers of the doubly-reduced and singly-protonated complex with tetrahedral (a) and trigonal (b) coordination geometry in acetonitrile. Selected charge density differences are presented, where excitation goes from blue to yellow.

11.6 Doubly-reduced, doubly-protonated complex

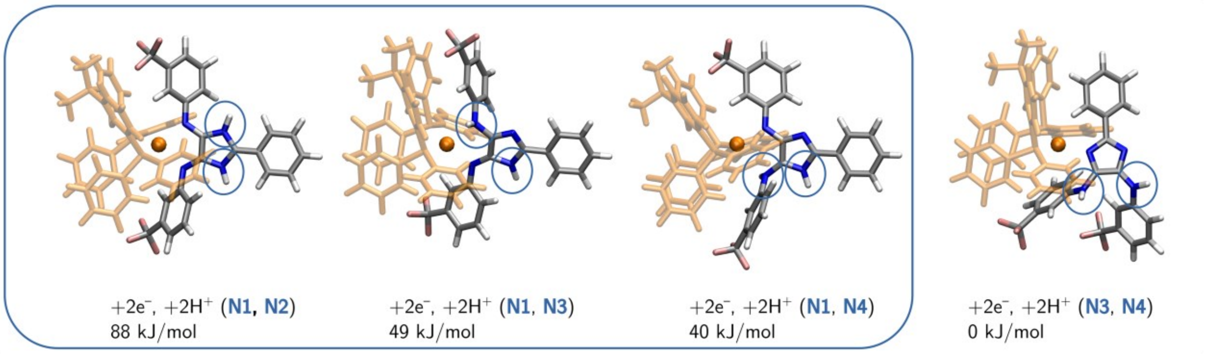

Figure S51. Visualization of the local minimum geometries of the doubly-reduced and singly-protonated forms of copper complex **2**. In the tetrahedral coordination geometry (blue box), protonation was performed at **N2**, **N3** and **N4** in addition to protonation at position **N1**, which was identified as minimum energy conformer, for the doubly-reduced and singly-protonated species. In trigonal coordination geometry, protonation was studied at **N4** and **N3**, whereas protonation at the latter position was found to form the minimum energy conformer for the trigonal doubly-reduced and singly-protonated species (see Figure S48). Relative energies with respect to the global minimum geometry (trigonal coordination sphere, protonation at **N3** and **N4**) are shown below each structure.

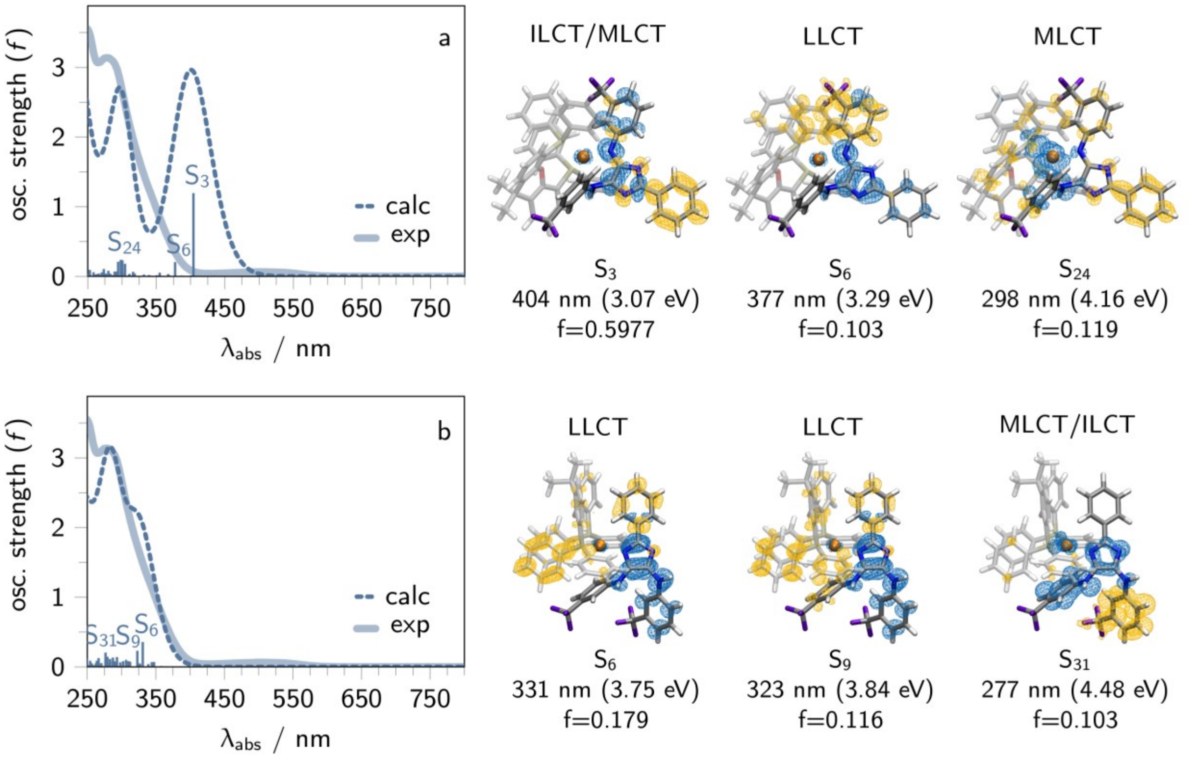

Figure S52. Simulated absorption spectra of the doubly-reduced and doubly-protonated complex in tetragonal (a) and trigonal (b) coordination sphere in acetonitrile. For selected bright transitions, the charge density differences are shown, with excitation illustrated from blue to yellow. The scaled experimental absorption spectrum, measured in acetonitrile solution, is shown as a thick line for comparison.

11.7 Calculated pK_a values and redox potentials

Table S8. Computed pK_a values for protonation of the singly-reduced and the doubly-reduced, singly-protonated complex with tetrahedral (Z=4) or trigonal planar (Z=3) coordination geometry.

Z	base (A^-)	acid (HA)	pK_a
3	$\mathbf{2}^{\bullet-}$	H**2**$^{\bullet}$ (**N3**)	6.3
3	$\mathbf{2}^{\bullet-}$	H**2**$^{\bullet}$ (**N4**)	5.2
3	$\mathbf{2}^{\bullet-}$	H**2**$^{\bullet}$ (**N1**)	2.5
4	$\mathbf{2}^{\bullet-}$	H**2**$^{\bullet}$ (**N1**)	11.0
4	$\mathbf{2}^{\bullet-}$	H**2**$^{\bullet}$ (**N2**)	10.6
4	$\mathbf{2}^{\bullet-}$	H**2**$^{\bullet}$ (**N3**)	8.2
4	$\mathbf{2}^{\bullet-}$	H**2**$^{\bullet}$ (**N4**)	7.6
3	H**2**$^-$ (**N3**)	H_2**2** (**N3, N4**)	22.2
4	H**2**$^-$ (**N1**)	H_2**2** (**N1, N4**)	14.8
4	H**2**$^-$ (**N1**)	H_2**2** (**N1, N3**)	13.4
4	H**2**$^-$ (**N1**)	H_2**2** (**N1, N2**)	6.6

Table S9. Computed reduction potentials ($E^0(A/A^{\bullet-})$) of the neutral and the singly-reduced and singly-protonated complex with tetrahedral (Z=4) coordination geometry.

Z	reducing agent (A)	Oxidizing agent ($A^{\bullet-}$)	E^0 ($A/A^{\bullet-}$)
4	**2**	$\mathbf{2}^{\bullet-}$	0.39 V
4	H**2**$^{\bullet}$ (**N1**)	H**2**$^-$ (**N1**)	0.40 V
4	H**2**$^{\bullet}$ (**N2**)	H**2**$^-$ (**N2**)	1.51 V
4	H**2**$^{\bullet}$ (**N3**)	H**2**$^-$ (**N3**)	1.59 V
4	H**2**$^{\bullet}$ (**N4**)	H**2**$^-$ (**N4**)	1.46 V

12 References

(1) Williams, D. B. G.; Lawton, M. Drying of organic solvents: quantitative evaluation of the efficiency of several desiccants. *Journal of Organic Chemistry* **2010**, *75* (24), 8351–8354. DOI: 10.1021/jo101589h. Published Online: Oct. 14, 2010.

(2) Schulz, M.; Hagmeyer, N.; Wehmeyer, F.; Lowe, G.; Rosenkranz, M.; Seidler, B.; Popov, A.; Streb, C.; Vos, J. G.; Dietzek, B. Photoinduced Charge Accumulation and Prolonged Multielectron Storage for the Separation of Light and Dark Reaction. *Journal of the American Chemical Society* **2020**, *142* (37), 15722–15728. DOI: 10.1021/jacs.0c03779. Published Online: Sep. 3, 2020.

(3) Seidler, B.; Tran, J. H.; Thomisch, L.; Vashistha, N.; Görls, H.; Liebing, P.; Schulz, M.; Dietzek-Ivanšić, B. Neutral, Heteroleptic [Cu(I)(PPh_3)$_2$(4*H*-imidazolato)] Complexes: Ligand Exchange Reactivity, Redox Properties, Excited-State Dynamics. *Chemistry* **2023**, *29* (25), e202203262. DOI: 10.1002/chem.202203262. Published Online: Mar. 27, 2023.

(4) Yang, D.; Fokas, D.; Li, J.; Yu, L.; Baldino, C. A Versatile Method for the Synthesis of Benzimidazoles from *o*-Nitroanilines and Aldehydes in One Step via a Reductive Cyclization. *Synthesis* **2005**, *2005* (01), 47–56. DOI: 10.1055/s-2004-834926.

(5) Jerschow, A.; Müller, N. 3D Diffusion-Ordered TOCSY for Slowly Diffusing Molecules. *Journal of Magnetic Resonance, Series A* **1996**, *123* (2), 222–225. DOI: 10.1006/jmra.1996.0241.

(6) Jerschow, A.; Müller, N. Suppression of Convection Artifacts in Stimulated-Echo Diffusion Experiments. Double-Stimulated-Echo Experiments. *Journal of Magnetic Resonance* **1997**, *125* (2), 372–375. DOI: 10.1006/jmre.1997.1123.

(7) Nair, S. S.; Bysewski, O. A.; Klosterhalfen, N.; Sittig, M.; Winter, A.; Schubert, U. S.; Dietzek-Ivanšić, B. Intramolecular Energy Transfer Competing with Light-Driven Intermolecular Proton Transfer in an Iron(II)-NHC Complex? A Query into the Role of Photobasic Ligands and MLCT States. *ACS Omega* **2024**, *9* (11), 13427–13439. DOI: 10.1021/acsomega.3c06196. Published Online: Mar. 7, 2024.

(8) Barthelmes, K.; Kübel, J.; Winter, A.; Wächtler, M.; Friebe, C.; Dietzek, B.; Schubert, U. S. New ruthenium bis(terpyridine) methanofullerene and pyrrolidinofullerene complexes: synthesis and electrochemical and photophysical properties. *Inorganic Chemistry* **2015**, *54* (7), 3159–3171. DOI: 10.1021/ic502431x. Published Online: Mar. 12, 2015.

(9) Siebert, R.; Akimov, D.; Schmitt, M.; Winter, A.; Schubert, U. S.; Dietzek, B.; Popp, J. Spectroscopic investigation of the ultrafast photoinduced dynamics in pi-conjugated terpyridines. *ChemPhysChem* **2009**, *10* (6), 910–919. DOI: 10.1002/cphc.200800847.

(10) Tran, J. H.; Traber, P.; Seidler, B.; Görls, H.; Gräfe, S.; Schulz, M. Ligand-Induced Donor State Destabilisation - A New Route to Panchromatically Absorbing Cu(I) Complexes. *Chemistry* **2022**, *28* (25), e202200121. DOI: 10.1002/chem.202200121. Published Online: Mar. 25, 2022.

(11) Seidler, B.; Sittig, M.; Zens, C.; Tran, J. H.; Müller, C.; Zhang, Y.; Schneider, K. R. A.; Görls, H.; Schubert, A.; Gräfe, S.; Schulz, M.; Dietzek, B. Modulating the Excited-State Decay Pathways of Cu(I) 4*H*-Imidazolate Complexes by Excitation Wavelength and Ligand Backbone. *The Journal of Physical Chemistry. B* **2021**, *125* (41), 11498–11511. DOI: 10.1021/acs.jpcb.1c06902. Published Online: Oct. 7, 2021.

(12) Schulz, M.; Dröge, F.; Herrmann-Westendorf, F.; Schindler, J.; Görls, H.; Presselt, M. Neutral, heteroleptic copper(I)-4*H*-imidazolate complexes: synthesis and characterization of their structural, spectral and redox properties. *Dalton Transactions* **2016**, *45* (11), 4835–4842. DOI: 10.1039/c5dt04435a. Published Online: Feb. 12, 2016.

(13) Vlcek, A. A.; Dodsworth, E. S.; Pietro, W. J.; Lever, A. B. P. Excited State Redox Potentials of Ruthenium Diimine Complexes; Correlations with Ground State Redox Potentials and Ligand Parameters. *Inorganic Chemistry* **1995**, *34* (7), 1906–1913. DOI: 10.1021/ic00111a043.

(14) Balzani, V.; Ceroni, P.; Juris, A. *Photochemistry and Photophysics*: *Concepts, Research, Applications*; John Wiley & Sons, 2014.

(15) Buzzetti, L.; Crisenza, G. E. M.; Melchiorre, P. Mechanistische Studien in der Photokatalyse. *Angewandte Chemie* **2019**, *131* (12), 3768–3786. DOI: 10.1002/ange.201809984.

(16) Müller, C.; Schulz, M.; Obst, M.; Zedler, L.; Gräfe, S.; Kupfer, S.; Dietzek, B. Role of MLCT States in the Franck-Condon Region of Neutral, Heteroleptic Cu(I)-4*H*-imidazolate Complexes: A Spectroscopic and Theoretical Study. *The Journal of Physical Chemistry. A* **2020**, *124* (33), 6607–6616. DOI: 10.1021/acs.jpca.0c04351. Published Online: Aug. 11, 2020.

(17) Sheldrick, G. M. SHELXT - integrated space-group and crystal-structure determination. *Acta Crystallographica. Section A, Foundations and Advances* **2015**, *71* (Pt 1), 3–8. DOI: 10.1107/S2053273314026370. Published Online: Jan. 1, 2015.

(18) Sheldrick, G. M. Crystal structure refinement with SHELXL. *Acta Crystallographica. Section C, Structural Chemistry* **2015**, *71* (Pt 1), 3–8. DOI: 10.1107/S2053229614024218. Published Online: Jan. 1, 2015.

(19) Dolomanov, O. V.; Bourhis, L. J.; Gildea, R. J.; Howard, J. A. K.; Puschmann, H. OLEX2 : a complete structure solution, refinement and analysis program. *Journal of Applied Crystallography* **2009**, *42* (2), 339–341. DOI: 10.1107/S0021889808042726.

(20) *Bruker AXS 2001, Apex4 and SADABS, Bruker AXS Inc., Madison, Wisconsin, USA*.

(21) Bai, Y.; Li, C.; Sun, W.; Zhao, G.; Shi, Z. Synthesis of *N*-methyl-*N*-[2-(methylamino)phenyl]carboxamide derivatives. *Huaxue Shiji* **2008**, *30* (6), 409–411.

(22) Neufeld, R.; Stalke, D. Accurate molecular weight determination of small molecules via DOSY-NMR by using external calibration curves with normalized diffusion coefficients. *Chemical Science* **2015**, *6* (6), 3354–3364. DOI: 10.1039/c5sc00670h. Published Online: Mar. 19, 2015.

(23) Schulz, M.; Reichardt, C.; Müller, C.; Schneider, K. R. A.; Holste, J.; Dietzek, B. Excited State Properties of Heteroleptic Cu(I) 4*H*-Imidazolate Complexes. *Inorganic Chemistry* **2017**, *56* (21), 12978–12986. DOI: 10.1021/acs.inorgchem.7b01680.

(24) Müller, C.; Pascher, T.; Eriksson, A.; Chabera, P.; Uhlig, J. KiMoPack: A python Package for Kinetic Modeling of the Chemical Mechanism. *The Journal of Physical Chemistry. A* **2022**, *126* (25), 4087–4099. DOI: 10.1021/acs.jpca.2c00907. Published Online: Jun. 14, 2022.

(25) Frisch, M. J.; Trucks, G. W.; Schlegel, H. B.; Scuseria, G. E.; Robb, M. A.; Cheeseman, J. R.; Scalmani, G.; Barone, V.; Petersson, G. A.; Nakatsuji, H.; Li, X.; Caricato, M.; Marenich, A. V.; Bloino, J.; Janesko, B. G.; Gomperts, R.; Mennucci, B.; Hratchian, H. P.; Ortiz, J. V.; Izmaylov, A. F.; Sonnenberg, J. L.; Williams-Young, D.; Ding, F.; Lipparini, F.; Egidi, F.; Goings, J.; Peng, B.; Petrone, A.; Henderson, T.; Ranasinghe, D.; Zakrzewski, V. G.; Gao, J.;

Rega, N.; Zheng, G.; Liang, W.; Hada, M.; Ehara, M.; Toyota, K.; Fukuda, R.; Hasegawa, J.; Ishida, M.; Nakajima, T.; Honda, Y.; Kitao, O.; Nakai, H.; Vreven, T.; Throssell, K.; Montgomery, J. A., Jr.; Peralta, J. E.; Ogliaro, F.; Bearpark, M. J.; Heyd, J. J.; Brothers, E. N.; Kudin, K. N.; Staroverov, V. N.; Keith, T. A.; Kobayashi, R.; Normand, J.; Raghavachari, K.; Rendell, A. P.; Burant, J. C.; Iyengar, S. S.; Tomasi, J.; Cossi, M.; Millam, J. M.; Klene, M.; Adamo, C.; Cammi, R.; Ochterski, J. W.; Martin, R. L.; Morokuma, K.; Farkas, O.; Foresman, J. B.; Fox, D. J. *Gaussian 16, Revision C.01*; Gaussian, Inc., Wallingford CT, 2016.

(26) Becke, A. D. Density-functional thermochemistry. III. The role of exact exchange. *The Journal of Chemical Physics* **1993**, *98* (7), 5648–5652. DOI: 10.1063/1.464913.

(27) Lee, C.; Yang, W.; Parr, R. G. Development of the Colle-Salvetti correlation-energy formula into a functional of the electron density. *Physical Review. B, Condensed Matter* **1988**, *37* (2), 785–789. DOI: 10.1103/physrevb.37.785.

(28) Weigend, F.; Ahlrichs, R. Balanced basis sets of split valence, triple zeta valence and quadruple zeta valence quality for H to Rn: Design and assessment of accuracy. *Physical Chemistry Chemical Physics* **2005**, *7* (18), 3297–3305. DOI: 10.1039/b508541a. Published Online: Aug. 4, 2005.

(29) Marenich, A. V.; Cramer, C. J.; Truhlar, D. G. Universal solvation model based on solute electron density and on a continuum model of the solvent defined by the bulk dielectric constant and atomic surface tensions. *The Journal of Physical Chemistry. B* **2009**, *113* (18), 6378–6396. DOI: 10.1021/jp810292n.

(30) Mennucci, B.; Cappelli, C.; Guido, C. A.; Cammi, R.; Tomasi, J. Structures and properties of electronically excited chromophores in solution from the polarizable continuum model coupled to the time-dependent density functional theory. *The Journal of Physical Chemistry. A* **2009**, *113* (13), 3009–3020. DOI: 10.1021/jp8094853.

(31) Grimme, S.; Ehrlich, S.; Goerigk, L. Effect of the damping function in dispersion corrected density functional theory. *Journal of Computational Chemistry* **2011**, *32* (7), 1456–1465. DOI: 10.1002/jcc.21759. Published Online: Mar. 1, 2011.
